# Supplementary material for: Non-Categorical Analyses Identify Rotenone-Induced ‘Parkinsonian’ Rats Benefiting from Nano-Emulsified Punicic Acid (Nano-PSO) in a Phenotypically Diverse Population: Implications for Translational Neurodegenerative Therapies
Source: Int J Mol Sci. 2024 Nov 25;25(23):12635. doi: 10.3390/ijms252312635 (PMC11640963; doi:10.3390/ijms252312635)
Supplement: Supplementary file 1 [file ijms-25-12635-s001.zip › Patent US20220339101A1.pdf]

US 20220339101A1

(19) United States

(12) Patent Application Publication

(10) Pub. No.: US 2022/0339101 A1

GABIZON et al.

(43) Pub. Date: Oct. 27, 2022

(54) **POMEGRANATE OIL FOR PREVENTING AND TREATING NEURODEGENERATIVE DISEASES**

(71) Applicants: **HADASIT MEDICAL RESEARCH SERVICES AND DEVELOPMENT LTD.**, Jerusalem (IL); **YISSUM RESEARCH DEVELOPMENT COMPANY OF THE HEBREW UNIVERSITY OF JERUSALEM LTD.**, Jerusalem (IL)

(72) Inventors: **Ruth GABIZON**, Jerusalem (IL); **Haim Ovadia**, Jerusalem (IL); **Oded Abramsky**, Jerusalem (IL); **Shlomo Magdassi**, Jerusalem (IL); **Liraz Larush**, Jerusalem (IL)

(73) Assignees: **HADASIT MEDICAL RESEARCH SERVICES AND DEVELOPMENT**, Jerusalem (IL); **YISSUM RESEARCH DEVELOPMENT COMPANY OF THE HEBREW UNIVERSITY OF JERUSALEM LTD.**, Jerusalem (IL)

(21) Appl. No.: **17/852,803**

(22) Filed: **Jun. 29, 2022**

Related U.S. Application Data

(63) Continuation of application No. 16/184,079, filed on Nov. 8, 2018, which is a continuation of application No. 14/523,408, filed on Oct. 24, 2014, now Pat. No. 10,154,961, which is a continuation of application No. PCT/IL2013/050353, filed on Apr. 24, 2013.

(60) Provisional application No. 61/684,760, filed on Aug. 19, 2012, provisional application No. 61/637,866, filed on Apr. 25, 2012.

Publication Classification

(51) **Int. Cl.**

A61K 9/107	(2006.01)
A23L 33/115	(2006.01)
A61K 47/14	(2006.01)
A61K 47/24	(2006.01)
A61K 47/26	(2006.01)
A61K 47/44	(2006.01)
A61K 31/202	(2006.01)
A61K 36/185	(2006.01)
A61K 45/06	(2006.01)
A61K 47/10	(2006.01)
A61K 47/22	(2006.01)

(52) **U.S. Cl.**

CPC ***A61K 9/107*** (2013.01); ***A23L 33/115*** (2016.08); ***A61K 47/14*** (2013.01); ***A61K 47/24*** (2013.01); ***A61K 47/26*** (2013.01); ***A61K 9/1075*** (2013.01); ***A61K 47/44*** (2013.01); ***A61K 31/202*** (2013.01); ***A61K 36/185*** (2013.01); ***A61K 45/06*** (2013.01); ***A61K 47/10*** (2013.01); ***A61K 47/22*** (2013.01)

(57) **ABSTRACT**

The present invention relates to the use of pomegranate oil and fractions thereof for preventing and treating neurodegenarative diseases. Particularly, the present invention relates to emulsions of the pomegranate oil or fractions thereof for the prevention and treatment of brain diseases, including Creutzfeldt-Jacob disease (CJD) and multiple sclerosis (MS).

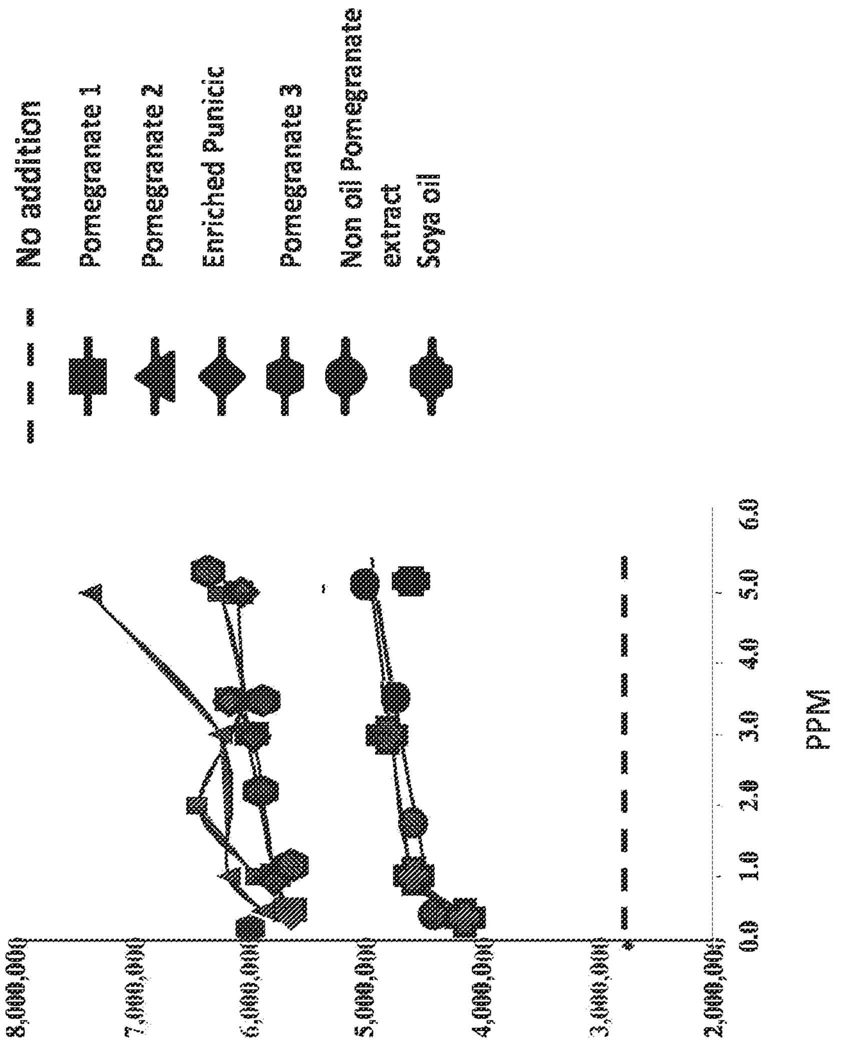
No addition
Pomegranate 1
Pomegranate 2
Enriched Punicic
Pomegranate 3
Non oil Pomegranate extract
Soya oil
8,000,000
7,000,000
6,000,000
5,000,000
4,000,000
3,000,000
2,000,000
0.0
1.0
2.0
3.0
4.0
5.0
6.0
PPM

FIG. 1

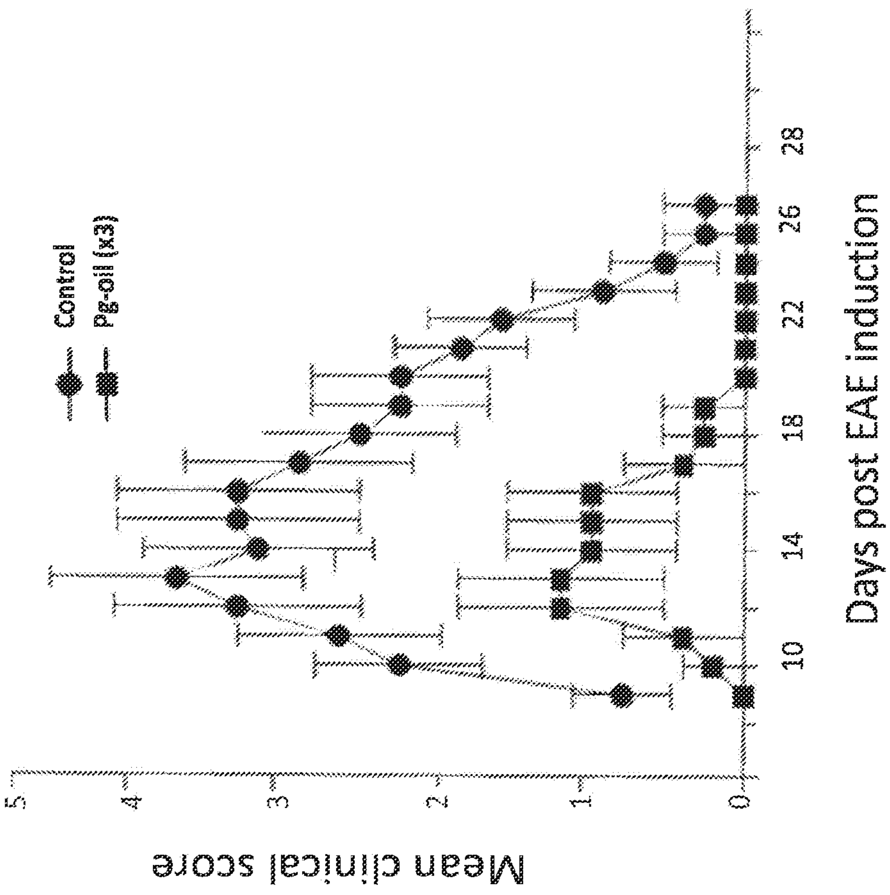
Control
Pg-oil (x3)
Mean clinical score
Days post EAE induction
0
1
2
3
4
5
10
14
18
22
26
28

FIG. 2B

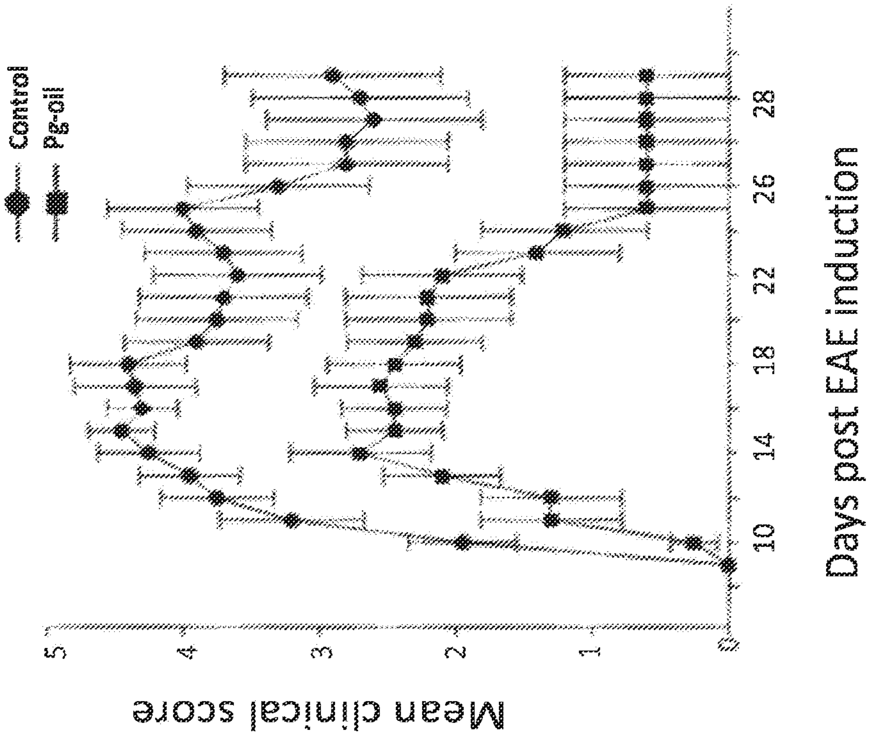
Control
Pg-oil
Mean clinical score
Days post EAE induction
0
1
2
3
4
5
10
14
18
22
26
28

FIG. 2A

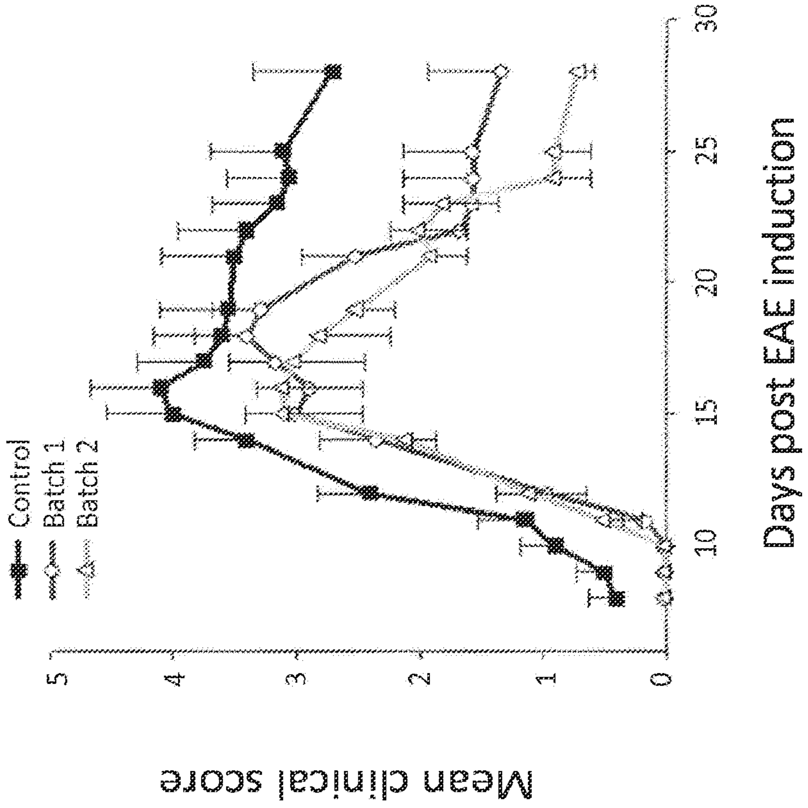
Control
Batch 1
Batch 2
Mean clinical score
0
1
2
3
4
5
10
15
20
25
30
Days post EAE induction

FIG. 3

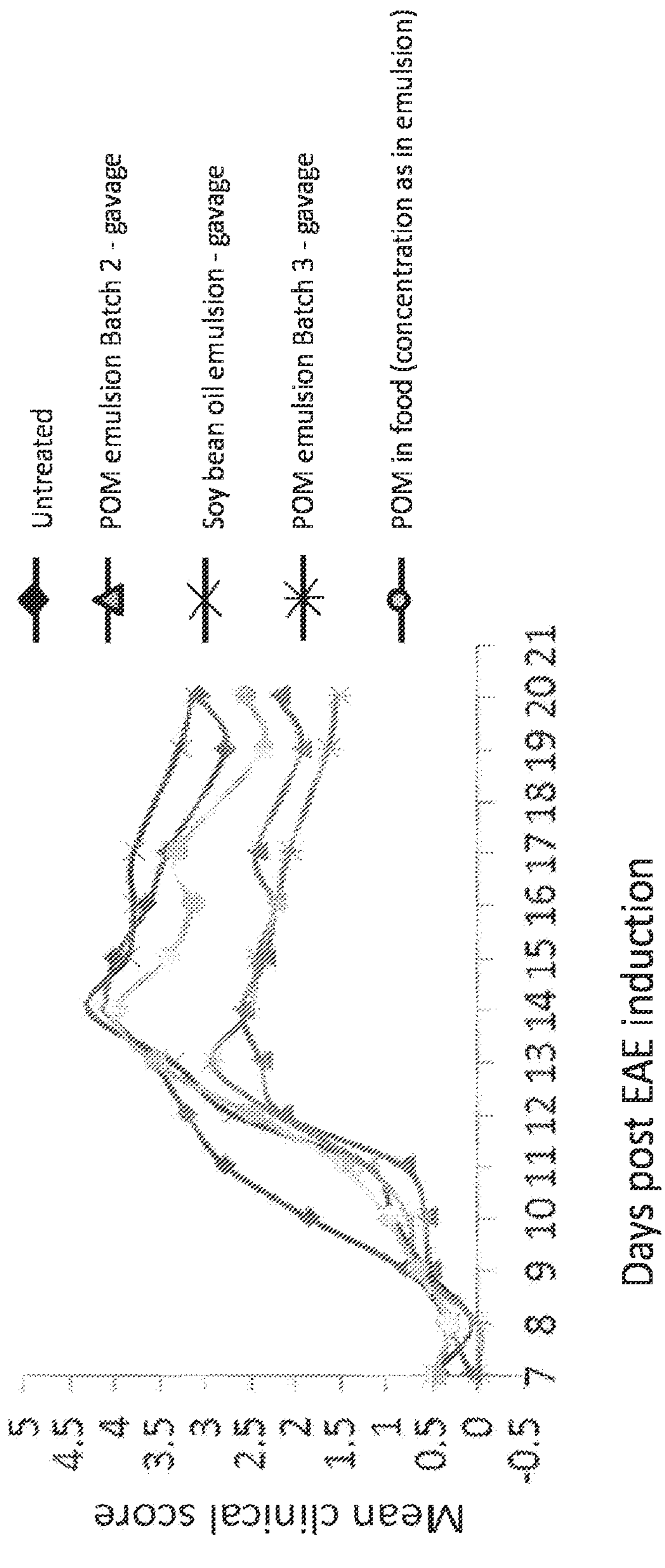
Untreated
POM emulsion Batch 2 - gavage
Soy bean oil emulsion - gavage
POM emulsion Batch 3 - gavage
POM in food (concentration as in emulsion)
Mean clinical score
5
4.5
4
3.5
3
2.5
2
1.5
1
0.5
0
-0.5
7 8 9 10 11 12 13 14 15 16 17 18 19 20 21
Days post EAE induction

FIG. 4

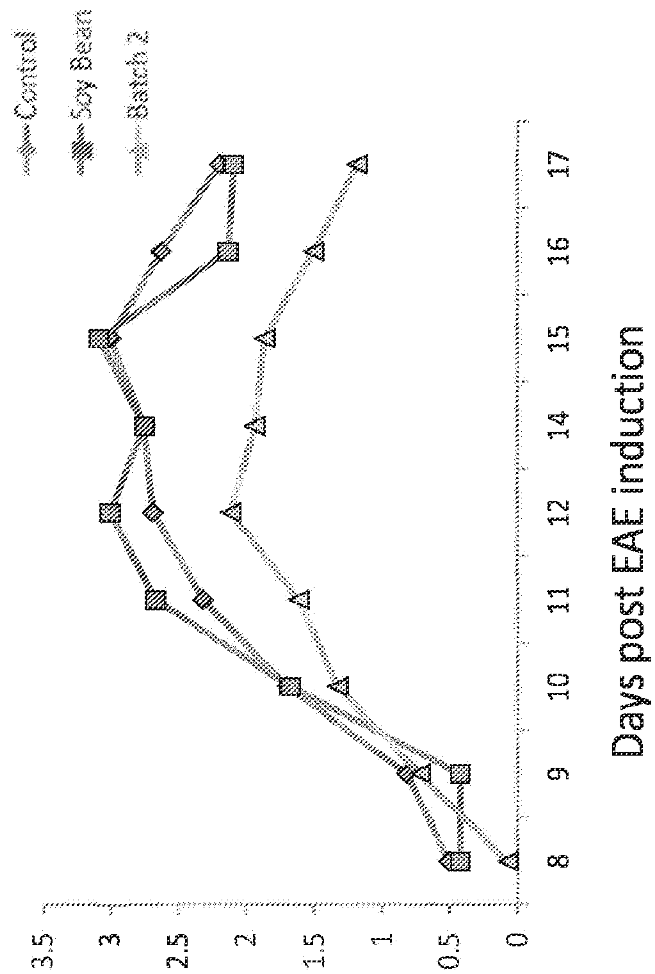
Mean clinical score
0
0.5
1
1.5
2
2.5
3
3.5
8
9
10
11
12
14
15
16
17
Days post EAE induction
Control
Soy Bean
Batch 2

FIG. 5

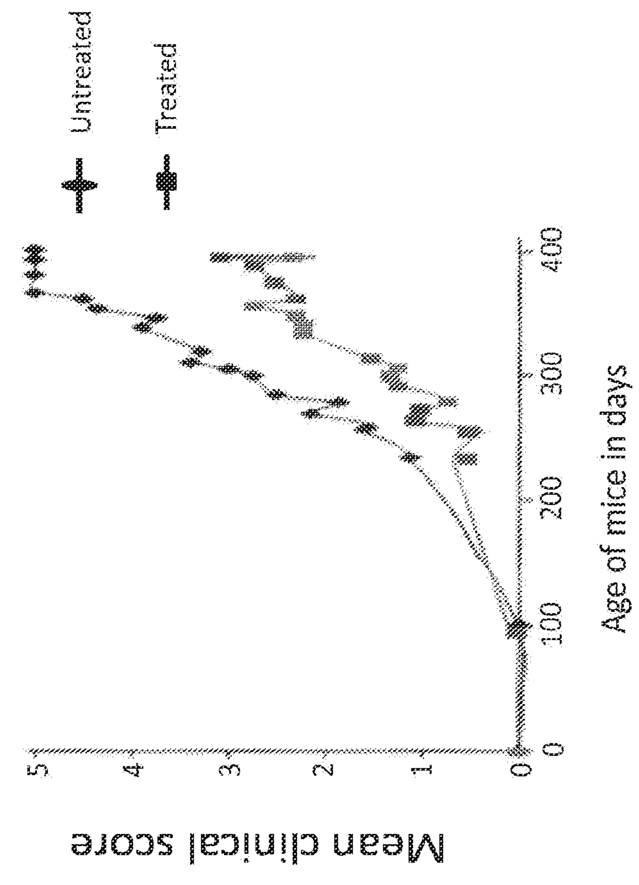

FIG. 6A

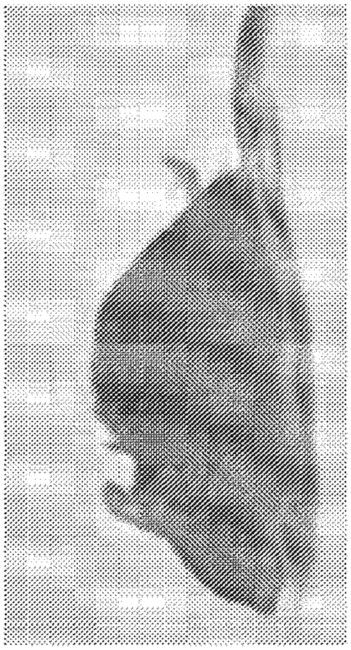

10 months old TgMHu2ME199K mouse

FIG. 6B

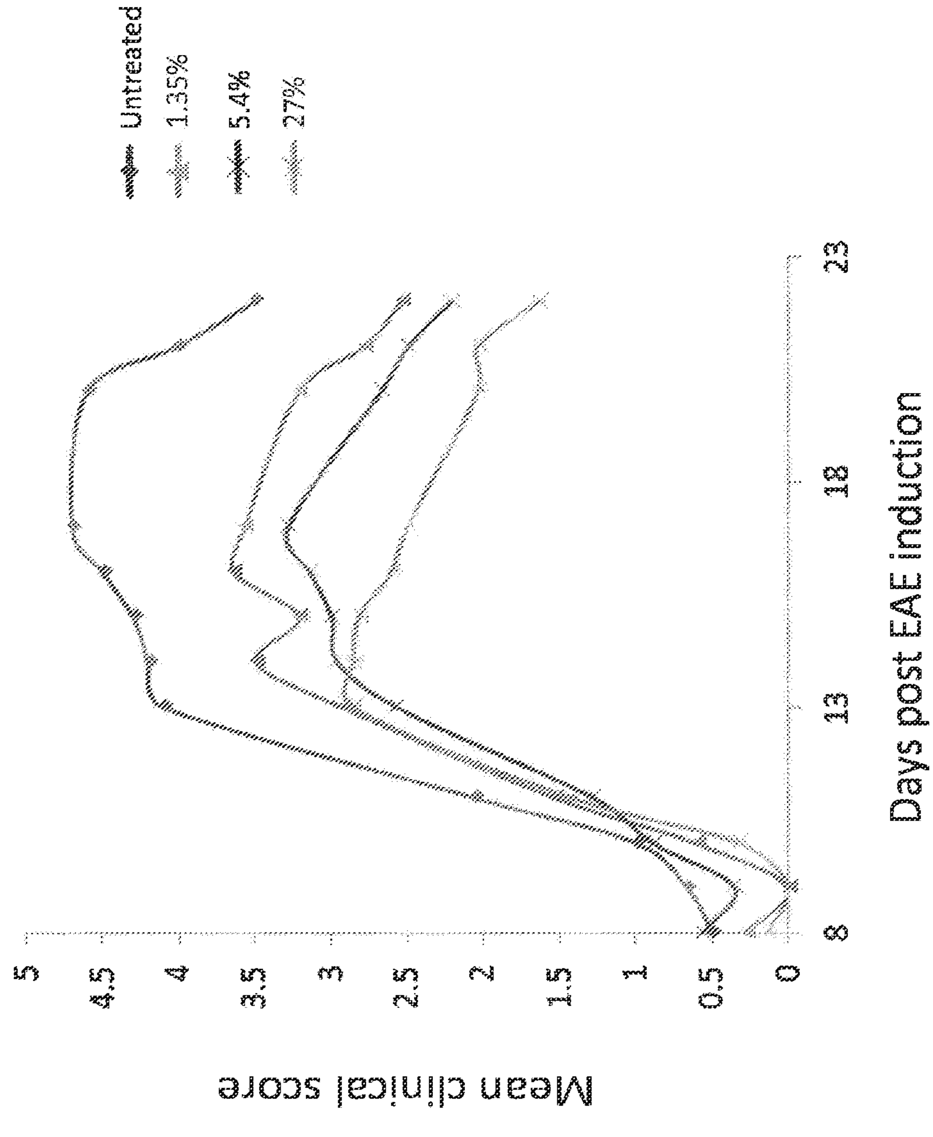
Mean clinical score
0
0.5
1
1.5
2
2.5
3
3.5
4
4.5
5
8
13
18
23
Days post EAE induction
Untreated
1.35%
5.4%
27%

FIG. 7

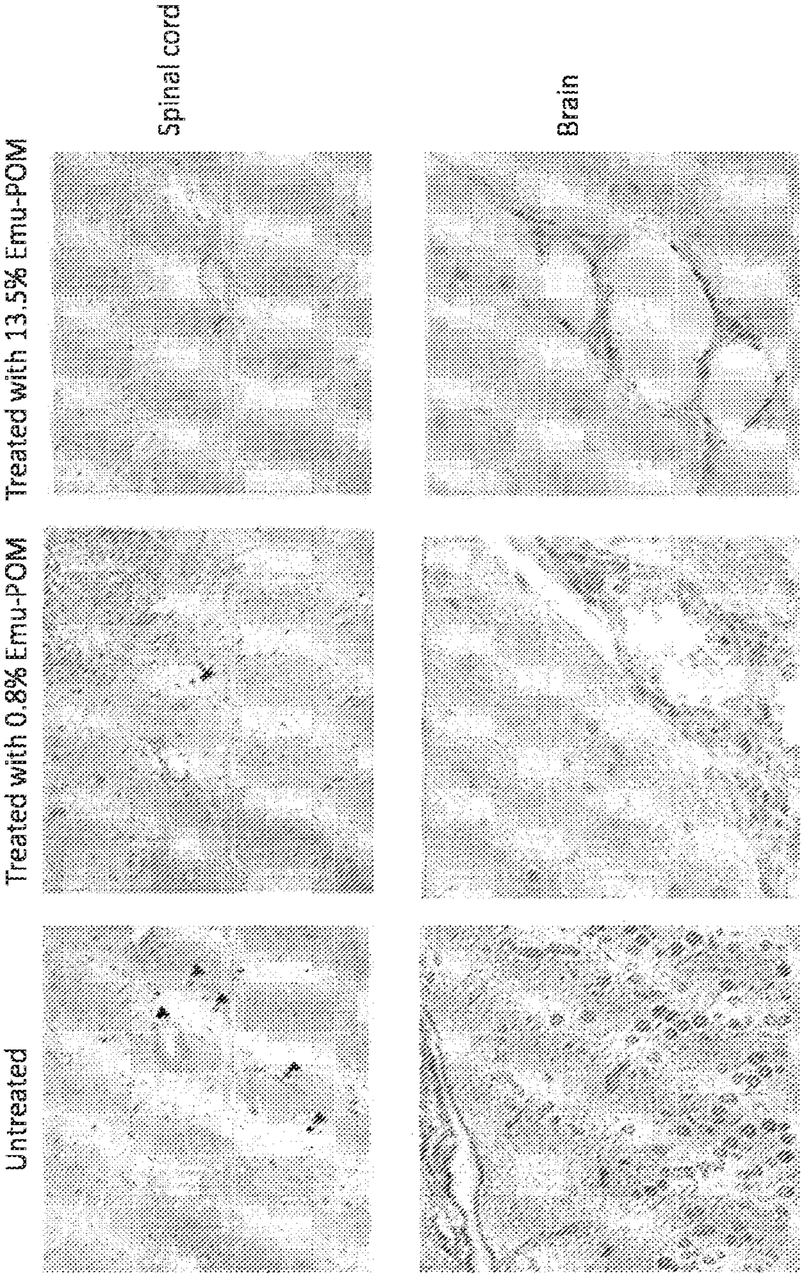
Untreated
Treated with 0.8% Emu-POM
Treated with 13.5% Emu-POM
Spinal cord
Brain

FIG. 8

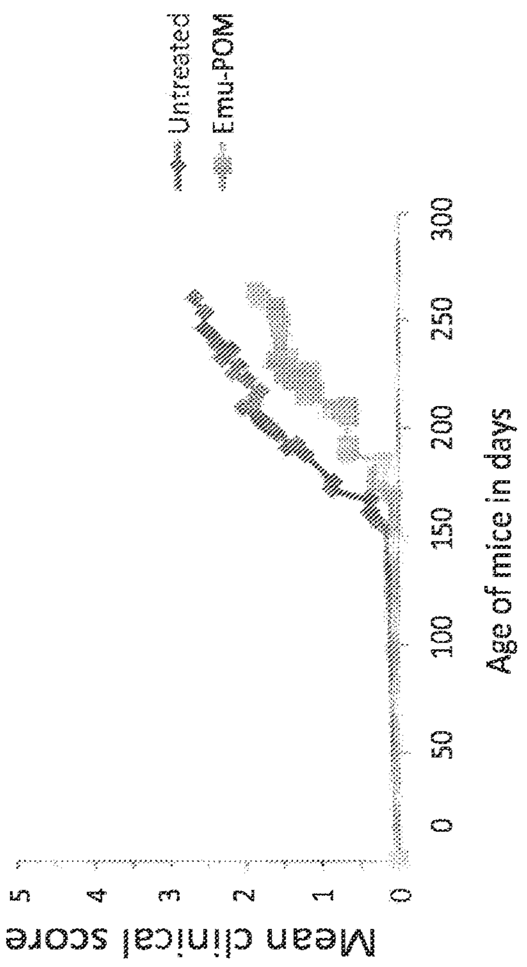
Mean clinical score
0
1
2
3
4
5
0
50
100
150
200
250
300
Age of mice in days
Untreated
Emu-POM

FIG. 9A

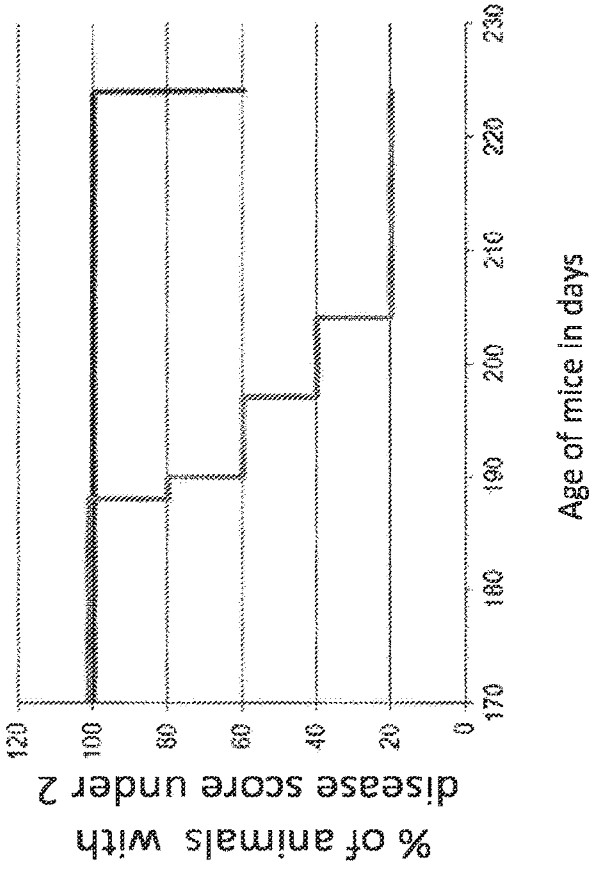
% of animals with disease score under 2
0
20
40
60
80
100
120
170
180
190
200
210
220
230
Age of mice in days

FIG. 9B

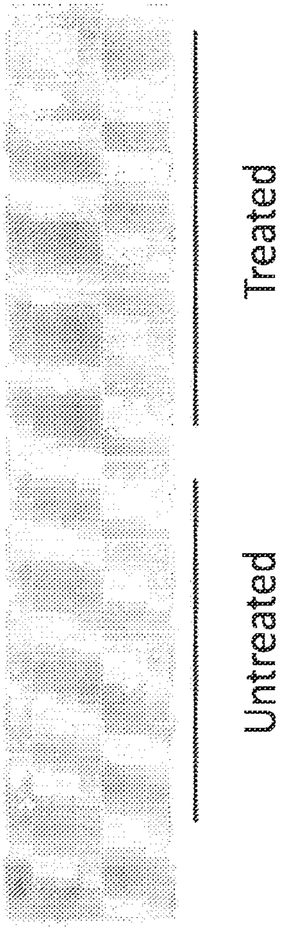
Untreated
Treated

FIG. 9C

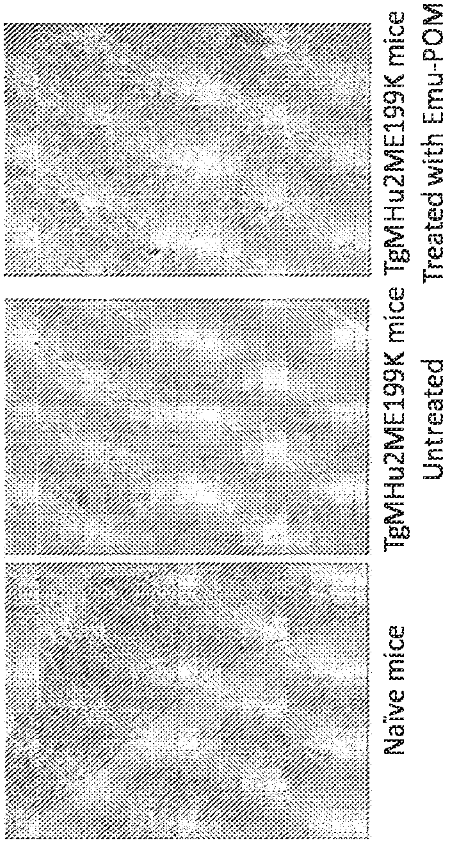
Naïve mice
TgMHu2ME199K mice
Untreated
TgMHu2ME199K mice
Treated with Emu-POM

FIG. 9D

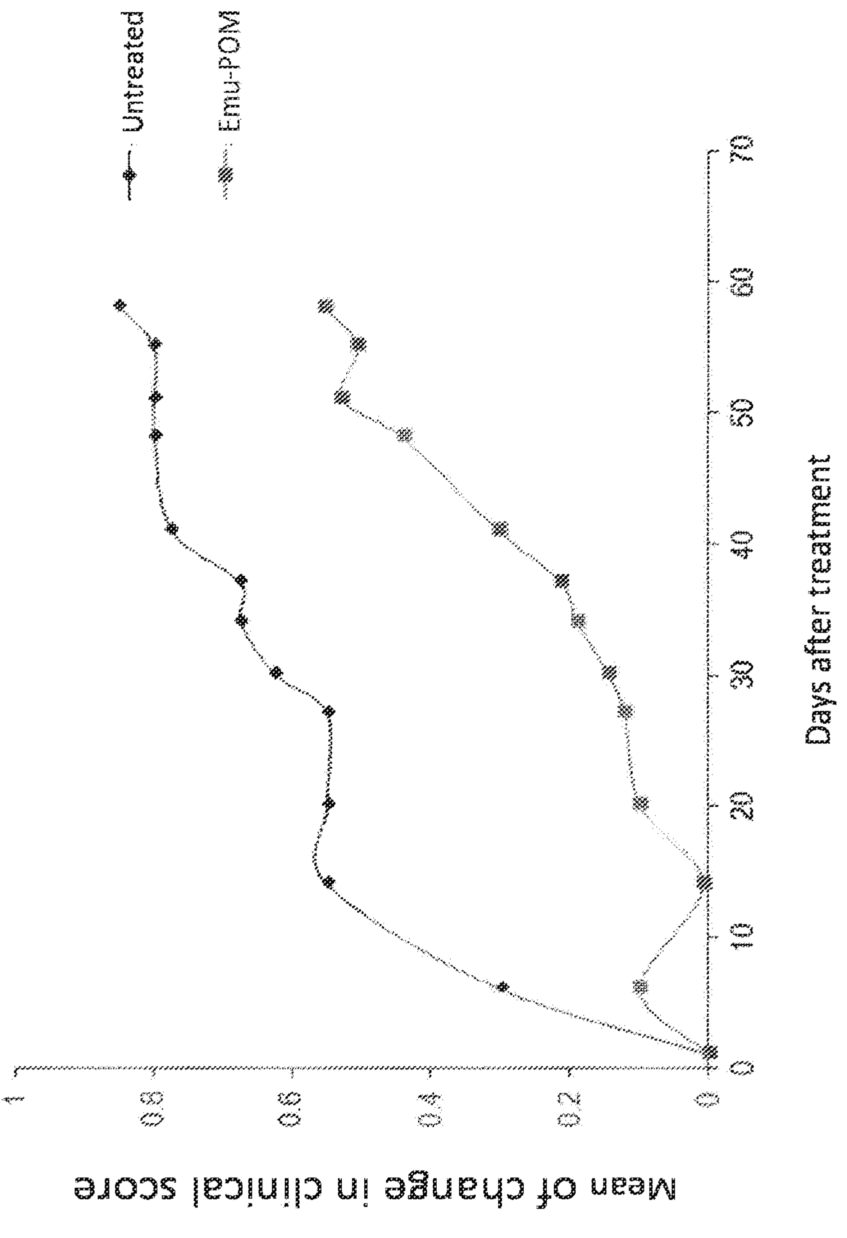
Untreated
Emu-POM
Mean of change in clinical score
0
0.2
0.4
0.6
0.8
1
0
10
20
30
40
50
60
70
Days after treatment

FIG. 10A

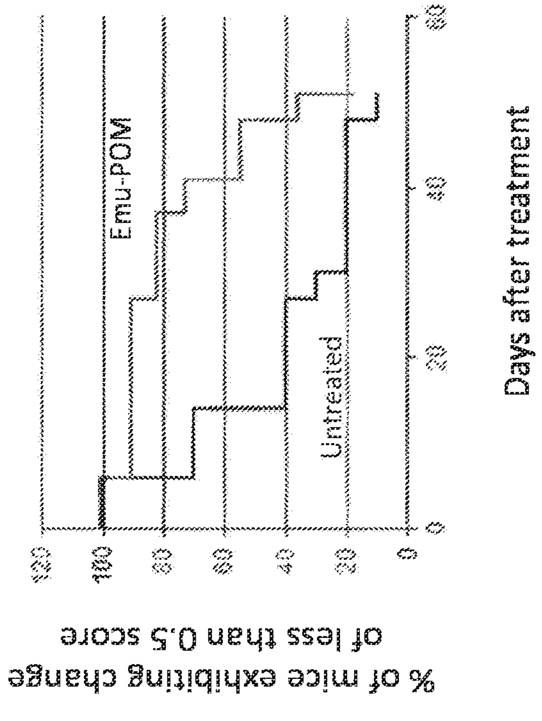
Emu-POM
Untreated
Days after treatment
% of mice exhibiting change of less than 0.5 score
0
20
40
60
80
100
120

FIG. 10B

POMEGRANATE OIL FOR PREVENTING AND TREATING NEURODEGENERATIVE DISEASES

FIELD OF THE INVENTION

[0001] The present invention relates to the use of pomegranate oil and fractions thereof for preventing and treating neurodegenerative diseases. Particularly, the present invention relates to emulsions comprising pomegranate oil or its major component punicic acid and use thereof for the prevention and treatment of brain diseases.

BACKGROUND OF THE INVENTION

[0002] Neurodegenerative disorders, particularly late onset brain disorders diseases affect an increasing number of individuals in the aging society of developed countries. Alzheimer's disease (AD) and Parkinson disease are the most prevalent diseases, but Creutzfeldt-Jacob disease (CJD). ALS and others are also increasingly diagnosed. Subjects suffering from multiple sclerosis (MS), a brain disease affecting many millions, also develop over the years features of neurodegenerative conditions.

[0003] In addition to individual features of brain diseases, such as demyelination in multiple sclerosis or misfolding and aggregation of designated proteins such as $PrPs^{Sc}$ in prion diseases (CJD in humans), there are several common denominators in all these conditions in the form of sensitivity to oxidative stress (Butterfield D A and Kanski J. 2001. Mech Ageing Dev 122:945-962). It was shown recently that brain lipids in the MS lesions are oxidized (Haider L et al., 2001. Brain 134:1914-1924), and this may also be the case for other neurodegenerative conditions (Uttara B et al., 2009. Curr Neuropharmacol 7:65-74). In MS, oxidation of lipids in the myclin sheet may be an important factor in the demyelination process, since changes in the lipid layer may result in the decompression and subsequent release of Myelin basic protein and other components from their membrane location. In the case of genetic CJD, a mutation in PrP, a copper binding glycosylphosphatidylinositol (GPI) anchored membrane glycoprotein, which changes the PrP conformation, is the main cause for disease progression. It has recently been shown that the mutated PrP is oxidized in its Methionine residues (Canello T et al., 2010. PLoS Pathog 2010, 6 (7):e1000977). Proteins damaged by oxidation are also associated with Alzheimer disease (oxidation of amyloid-β, Aβ), Parkinson disease (oxidation of synuclein) and the aging process in general (Stadtman ER 2001. Ann NY Acad Sci 928:22-38).

[0004] Most of the neurodegenerative diseases are of slow progression, and thus a long term economic effort is required for the medical system to cope with the patient's management. For some of these diseases no treatment of any kind is available while for others, partial treatment with severe side effects is being implemented. Thus, there is an ongoing search for effective and safe medications, including from natural sources.

[0005] Pomegranate (*Punica granatum*) has long been recognized as a fruit with many benefits for health. For example, pomegranate is known as the richest plant source of the female steroid hormone estrone and recently, the male hormone testosterone and another female steroid, estriol, have also been discovered in pomegranate seed oil. A wide range of polyphenolic compounds including flavonoids, anthocyanins and tannins have been characterized both in pomegranate juice and pericarp. Further, these polyphenols, extracted both from the fermented juice and the oil, have been shown to be potent antioxidant in vitro and to additionally inhibit the cicosanoid enzyme lipoxygenase. Polyphenols extracted from pomegranate seed oil have also been shown to be significantly inhibitory of another cicosanoid pathway enzyme, cyclooxygenase (Schubert S Y et al., 1999. J Ethnopharmacology 66 (1):11-17).

[0006] U.S. Application Publication No. 20020012710 discloses a cancer chemo-preventive mixture of a pomegranate seed oil product and a pomegranate juice product and a pharmaceutical composition containing same. According to some disclosed embodiments, a pomegranate peel product is further included. Further disclosed are a selective estrogen receptor modulator and other biologically active compounds derived from pomegranates as well as methods of use thereof.

[0007] International Application Publication No. WO 2005/070412 discloses a method of enhancing the immune response of an animal, including mammals and humans, to prevent or ameliorate immunoinflammatory diseases such as Inflammatory Bowel Disease (IBD), to increase immune system development, to maintain or increase CD4 and CD8 T lymphocyte levels, to increase immune function, to increase immune response against viruses and to prevent or ameliorate the Metabolic Syndrome, Type 2 diabetes and obesity by administering orally or parentally a therapeutically effective amount of punicic acid to the animal.

[0008] International Application Publication No. WO 2009/016620 discloses methods of producing pomegranate sprouts and pomegranate sprout preparation as well as food or feed products comprising same. The pomegranate sprouts and preparations may be consumed for general health, but may be especially advantage to subjects susceptible to conditions associated with oxidative stress, such as arthero-sclerosis, diabetes, cancer, cardiovascular disease, liver disease and individuals at risk of developing neurodegenerative diseases such as Alzheimer's disease.

[0009] European Patent No. EP1175901 discloses a method of promoting binding of a peroxisome proliferator-activated receptor to a target gene sequence and promoting gene expression downstream thereof by administering a peroxisome proliferator-activated receptor agonist. The agonist is a conjugated unsaturated fatty acids having 10 to 26 carbon atoms and containing a conjugated trienoic or a conjugated tetraenoic structure and salts and ester derivatives thereof, an example being punicic acid. Particularly, the patent discloses use of the method for reduction of visceral fat amount or suppression of visceral fat accumulation, prevention or amelioration of lipid metabolism abnormalities, prevention or amelioration of glucose metabolism abnormalities, or prevention or treatment of cancer can be carried out in the human or animal.

[0010] U.S. Application Publication No. 20110065662 discloses compounds, extracts and compositions thereof, and methods of using the same, to treat neurodegenerative disorders and/or improve brain health. In certain embodiments, said compounds are pomegranate flavonoids.

[0011] The therapeutic use of pomegranate oil or its derivative is still limited due to relatively high amounts of crude oil that should be consumed. In addition, many beneficial effects of pomegranate oil have been demonstrate

only in in vitro cell culture models, due to the insufficient availability of orally administered oil.

[0012] Progression of neurodegenerative disease is associated with irreversible death of brain cells. Thus, there is a clear unmet need not only for a treatment after diagnosis but also for prevention means in cases where susceptibility can be identified in advance. There is further unmeet need for efficacious delivery means of pomegranate oils and derivative thereof to exert their therapeutic effects.

SUMMARY OF THE INVENTION

[0013] The present invention discloses means and methods for the use of pomegranate oil, or of the pomegranate oil major component punicic acid, for the prevention and treatment of neurodegenerative diseases including Creutzfeldt-Jacob disease (CJD), multiple sclerosis (MS) and other conditions associated with oxidative stress of components within the central nervous system (CNS).

[0014] The present invention is based in part on the unexpected discovery that pomegranate oil and its major component punicic acid is highly effective in preventing cooper-induced death of model cells of the genetic prion disease as well in reducing the disease symptoms and progress in model transgenic mice afflicted with the prion disease, and further in reducing the disease symptoms in mice induced for experimental autoimmune encephalomyelitis (EAE). Surprisingly, these effects were significantly enhanced when the pomegranate oil was administered in sub-micron emulsion formulations.

[0015] Thus, according to one aspect, the present invention provides a method of preventing and/or treating a neurodegenerative disease, comprising administering to a subject having or at risk of developing a neurodegenerative disease a therapeutically effective amount of pomegranate seed oil or a fraction thereof.

[0016] According to certain embodiments, the pomegranate seed oil comprises at least 50%, typically 60%, more typically 75%, 80% or more punicic acid. According to other embodiments, the pomegranate oil is enriched to contain at least 90%, 95% or more punicic acid. According to yet additional embodiments, the method comprises administering therapeutically effective amount of pomegranate oil fraction, wherein the fraction is 100% punicic acid.

[0017] The pomegranate oil can be produced employing any method as is known to a person skilled in the art. According to certain embodiments, the pomegranate oil is produced by a process selected from the group consisting of, but not limited to, expeller pressing, solvent extraction and supercritical fluid extraction with carbon dioxide.

[0018] According to other embodiments, punicic acid is used. According to these embodiments, the punicic acid can be isolated from pomegranate seed oil or produced synthetically. According to yet additional embodiments, pharmaceutically acceptable esters, salts, metabolites of punicic acid or combinations thereof can be used. According to certain embodiments, punicic acid is used in its free acid form.

[0019] According to still further embodiments the pomegranate seed oil is produced from a material selected from the group consisting of pomegranate seeds and pomegranate seed cake.

[0020] The pomegranate seed oil, punicic acid, salts or esters thereof can be administered per se, or within a formulation as is known to a person skilled in the art.

[0021] While the oil and/or fatty acids of the present invention can be easily incorporated into the human diet, their in vivo activity is limited by chemical degradation, poor bioavailability, reduced distribution to the CNS, and sub-pharmacological doses. The present invention now discloses that formulating the pomegranate oil or fractions thereof into emulsions significantly enhances the stability and bioavailability of these compounds.

[0022] Thus, according to certain embodiments, the pomegranate seed oil, punicic acid, salts or esters thereof are formulated into emulsions, particularly oil in water submicron emulsions. In these emulsions the oil component is essentially the therapeutically active agent dispersed in the water component, with the mean droplet size being in the submicron range, i.e., below about 1.0 μm, typically between about 0.03 μm and 1.0 μm, between about 0.05 μm and 0.5 μm or between 0.05 μm and 0.4 μM. The emulsion further comprises at least one emulsifying agent and/or other surfactants.

[0023] According to certain embodiments, the emulsion further comprises additional additives, selected from, but not limited to, preservatives, co-surfactants, buffers, oils, rheological agents, flavor agents, antioxidants and any combination thereof. According to certain embodiments, all the emulsion ingredients are of food grade. According to other embodiments, all the emulsion ingredients are pharmaceutically acceptable.

[0024] According to certain typical embodiments, the subject is human.

[0025] According to certain embodiments, the neurodegenerative disease is selected from the group consisting of Creutzfeldt-Jacob disease (CJD), multiple sclerosis (MS), Alzheimer disease, Parkinson disease, and amyotrophic lateral sclerosis (ALS). Each possibility represents a separate embodiment of the present invention.

[0026] According to certain particular embodiments, the neurodegenerative disease is Creutzfeldt-Jacob disease (CJD). According to other certain particular embodiments, the neurodegenerative disease is multiple sclerosis (MS).

[0027] According to certain embodiments, preventing a neurodegenerative disease comprises at least one of preventing the appearance of the disease symptoms, delaying the appearance of the disease symptoms and reducing further progression of the disease.

[0028] According to certain embodiments, the methods of the present invention further comprise a step of classifying the human subject as having or being at risk for developing a neurodegenerative disease before administering the pomegranate oil or punicic acid fraction thereof.

[0029] According to certain embodiments, classifying the subject as having or being at risk to have the neurodegenerative disease comprises analyzing the genetic profile of said subject.

[0030] According to other embodiments, the risk assessment in based on phenotypic symptoms associated with the disease. It is to be understood that the type of the genetic profile and/or phenotype depends on the particular neurodegenerative disease at question, and its setting is well within the skills of a person with knowledge in the art.

[0031] According to certain embodiments, the neurodegenerative disease is CJD, and classifying the subject to have or being at risk to have CJD comprises analyzing a sample containing a genetic material obtained from said subject for the presence of at least one mutation in the PrP

gene, wherein the presence of such mutation classifies said subject as having or being at risk to have CJD.

[0032] According to certain embodiments, the neurodegenerative disease is MS, and classifying the subject to have or being at risk to have CJD comprises analyzing said subject for an array of reversible first symptoms known to a person skilled in the art, wherein the presence of at least one reversible symptom classifies said subject as having or being at risk to further develop MS.

[0033] According to certain embodiments, the first symptom array comprises optical neuritis.

[0034] According to certain embodiments, the method of the present invention comprises administering to the subject in need thereof a pharmaceutical composition comprising a therapeutically effective amount of pomegranate oil or punicic acid, salts or derivative thereof further comprising a therapeutically acceptable diluents or carrier.

[0035] According to some embodiments, the pharmaceutical composition is in the form of a liquid emulsion as described hereinabove. The emulsions of the present invention can be further formulated to form powders, nano-fibers, capsules, granules, tablets, suspensions and the like as is known to a person skilled in the art. The pharmaceutical composition can be formulated for administration in a form selected from the group consisting of oral, parenteral, transdermal or administration via inhalation. Each possibility represents a separate embodiment of the present invention. According to certain embodiments, the pharmaceutical composition comprises at least one additional active agent.

[0036] According to certain embodiments, the pomegranate seed oil, punicic acid, salts or esters thereof are formulated into self emulsifying drug delivery system (SEDDS). According to typical embodiments, the SEDDS comprises the pomegranate seed oil, punicic acid, salts or esters thereof and at least one of emulsifying agent, a surfactant or a combination thereof.

[0037] According to some embodiments, the emulsifying and/or surfactant agents are of pharmaceutical grade. According to other embodiments, the emulsifying and/or surfactant agents are of food (nutraceutical) grade. According to certain typical embodiments, the SEDDS comprises pomegranate oil, derivative, salts or ester thereof; polyoxyethylene sorbitan triglyceride ester (Tween) 80; and Polyoxyl 40 Hydrogenated Castor Oil (Cremophor RH 40).

[0038] According to other typical embodiments, the SEDDS comprises pomegranate oil, derivative, salts or ester thereof: polyoxyethylene sorbitan triglyceride ester (Tween) 80; sorbitan fatty acid ester (Span 80), and ethanol.

[0039] Encapsulation of the SEDDS may be achieved by conventional means, e.g. using gelatin capsules, optionally internally or externally coated with agent for a sustained and/or delayed release, including for example a material which is insoluble at neutral pH but soluble at the pH of gastric contents.

[0040] According to other embodiments, the pomegranate seed oil, punicic acid, emulsions or SEDDS comprising same can be administered in a form selected from the group consisting of nutraceutical formulation, a medical food (also known as dietary food for special medical purpose), a functional food, a food additive, or a dietary supplement (also known as phytomedical product). The compositions may also contain an additional therapeutic agent, or may be administered in combination with another therapeutic compound.

[0041] According to other embodiments, the pomegranate seed oil, punicic acid or emulsions comprising same are administered parenterally, e.g., intravenously, subcutaneously, intradermally, or intramuscularly. According to further embodiments, the pomegranate seed oil, punicic acid or emulsions comprising same are administered via inhalation. According to still further embodiments, the pomegranate seed oil, punicic acid or emulsions comprising are administered transdermally.

[0042] According to some embodiments, the method of the present invention further comprises co-administration of additional active agent. According to certain embodiments, the additional active agent is a naturally derived antioxidant, including, but not limited to, Sulforaphane, Curcumin and Green tea catechins. According to other embodiments, the additional active agent is known to have an effect on the particular neurodegenerative disease. According to certain embodiments, when the neurodegenerative disease is MS, the co-administered agent is the known drug Copaxone (Glatiramer acetate). The additional active agent can be administered in the same composition comprising the pomegranate oil or derivative thereof or it can be administered concomitantly in a separate composition.

[0043] The therapeutic amount required and the form and regime of administration depend on the type of the disease to be treated and parameters related thereto, including severity and stage, and on parameters related to the subject to be treated, including age, gender and weight. A significant advantage of the method of the present invention is in that pomegranate oil and its major component punicic acid are not toxic, such that there is no known upper limit to the amount that may be administered to the subject in need. It is also to be understood that the subject can be at all ages and health conditions. Accordingly, the method of the present invention can be used for treating vulnerable populations including elderly, obese, diabetic, sick or very young subjects, as well as healthy subject not yet showing any signs of the disease.

[0044] According to additional aspect, the present invention provides an oil-in-water emulsion delivery system for pomegranate oil, derivatives and salts thereof, the delivery system comprising an oil phase comprising pomegranate seed oil, derivatives and salts thereof at a concentration of at least 5% (w/w); emulsifier comprising at least one emulsifying agent; and an aqueous phase. According to a further aspect, the present invention provides a self emulsifying drug delivery system (SEDDS) for pomegranate seed oil, derivatives and salts thereof, the delivery system comprising an oil phase comprising pomegranate seed oil, derivatives and salts thereof at a concentration of at least 5% (w/w) and emulsifier comprising at least one emulsifying agent. According to certain embodiments, the delivery system further comprises at least one additional additive selected from the group consisting of preservatives, antioxidants, co-surfactants, buffers, oils, rheological agents, flavor agents and any combination thereof.

[0045] Other objects, features and advantages of the present invention will become clear from the following description and drawings.

BRIEF DESCRIPTION OF THE FIGURES

[0046] FIG. **1** shows the effect of several preparations of pomegranate seed oil and punicic acid on the survival of fibroblast isolated from TgMHu2ME199K mice and incu-

bated in the presence of 400 μM $CuSO_4$. TgMHu2ME199K mice were heterozygous to the PrP mutation (i.e. carrying a wild type and a mutant PrP alleles) to mimic heterozygous human.

[0047] FIGS. **2**A-**2**B demonstrate the effect of pomegranate seed oil on the disease scores in mice induced for Experimental Autoimmune Encephalomyelitis (EAE). FIG. **2**A: pomegranate oil administered in the food at a concentration of 25 ml/Kg food. FIG. **2**B: pomegranate oil administered in the food at a concentration of 75 ml/Kg food. PG-oil: Pomegranate oil.

[0048] FIG. **3** demonstrates the effect of emulsified pomegranate seed oil on the disease scores in mice induced for Experimental autoimmune encephalomyelitis (EAE). Batch 1: Emulsified pomegranate oil having an average droplet size of 150 nm. Batch 2: Emulsified pomegranate oil having an average droplet size of 80-100 nm.

[0049] FIG. **4** shows a comparison between the effect of soy oil and pomegranate oil on the disease scores in mice induced for Experimental autoimmune encephalomyelitis (EAE). Test compounds were administered to the mice from the first day of the experiment.

[0050] FIG. **5** shows a comparison between the effect of soy oil and pomegranate oil on the disease scores in mice induced for Experimental autoimmune encephalomyelitis (EAE). Test compounds were administered to the mice from day 7 of the experiment.

[0051] FIGS. **6**A-**6**B show the effect of pomegranate oil on the onset of prion disease in TgMHu2ME199K mice. FIG. **6**A: The effect of punicic acid on disease onset and progression. Scores of disease severity were as follows: [1]: Hind limbs weakness [2] Hind limb partial paralysis [3] Full paralysis in one limb [4] Full hind limb paralysis [5] Death. FIG. **6**B: ten months old TgMHu2ME199K untreated mouse.

[0052] FIG. **7** demonstrates the effect of pomegranate seed oil on the disease scores in mice induced for Experimental Autoimmune Encephalomyelitis (EAE). The pomegranate oil was emulsified using pressure homogenizations to reach pomegranate oil concentration of 27%. The emulsion was administered at the initial pomegranate oil concentration (27%); 5.4% and 1.35%.

[0053] FIG. **8** shows spinal cord and brain sample stained with anti-CD3 antibodies. The figure presents samples taken from mice induced for EAE which received pomegranate oil emulsion containing 0.8% or 13.5% pomegranate oil (Emu-POM) compared to samples taken from mice induced for EAE that did not receive the pomegranate oil emulsion (untreated). Upper panel: samples taken from spinal cord. Lower panel: sample taken from brain.

[0054] FIGS. **9**A-**9**D demonstrate the effect pomegranate oil on prion disease in TgMHu2ME199K mice. FIG. **9**A-B: disease progress after 50 days of administration of pomegranate oil emulsion (Emu-POM) by gavage presented as average disease score (A) and as the percentage of mice with a disease score below 2 (B). FIG. **9**C: accumulation of Proteinase K (PK) resistant PrP protein in treated and untreated mice. FIG. **9**D: Brain cerebellar samples stained with anti-synaptophysin antibodies.

[0055] FIGS. **10**A-**10**B show the effect of pomegranate oil provided in the drinking water on the disease progress in sick TgMHu2ME199K mice. FIG. **10**A: disease progresses presented as the average change in the disease score. FIG. **10**B: disease progress presented as percentage of subjects showing change of less than 0.5 score.

DETAILED DESCRIPTION OF THE INVENTION

[0056] The present invention relates in general to the field of substances derived from natural sources which are effective in improving the health of a human subject. Particularly, the present invention discloses the use of pomegranate oil or of pure punicic acid for prophylactic as well as therapeutic treatment of neurodegenerative diseases. Without wishing to be bound by any specific theory or mechanism of action, the present invention now shows that pomegranate oil, particularly pomegranate oil delivered in the form of an oil in water submicron emulsion is highly efficient in the prevention and treatment of neurodegenerative disease associated with oxidative stress, including, but not limited to. Creutzfeldt-Jacob disease, (CJD), multiple sclerosis (MS) Parkinson disease and Alzheimer's disease.

Definitions

[0057] As used herein, the terms "pomegranate oil" and "pomegranate seed oil" are used herein interchangeably, referring to the oil fraction obtained from the seeds of the fruit of pomegranate (*Punica granatum*). The pomegranate oil can be extracted from the seeds or from seed cakes by any method known in the art. According to typical embodiments, the oil is extracted using one of press extraction, solvent extraction or supercritical fluid extraction with carbon dioxide.

[0058] As used herein, the term "punicic acid" refers to a conjugated linolenic acid isomer containing cis-9, trans-11, cis-13 double bonds in the Cis carbon chain. The term further refers to punicic acid non-toxic salts, active esters, active isomers, active metabolites, structural lipids containing punicic acid, and mixtures thereof. Punicic acid is also known as trichosanic acid and is found in the seed oil of *Punica granatum* (Punicaceae, Pomegranate) and *Trichosanthes anguina* (Cucurbitaceae, snake gourd). Punicic acid constitutes approximately 60-90% of the oil of the pomegranate seed. Non-toxic salts of punicic acid include, for example, alkyl esters having from 1 to 6 carbon atoms in the alkyl group, as well as mono-, di- and tri-glycerides, and mixtures thereof.

[0059] Active isomers of punicic acid include geometrical isomers such as eleostearic acid (cis-9, trans-11, trans-13 octadecatrienoic acid) and its non-toxic salts (e. g., sodium, potassium, calcium and magnesium salts) and its active esters (e. g., alkyl esters having from 1 to 6 carbon atoms in the alkyl group, as well as mono-, di- and tri-glycerides, and mixtures thereof.

[0060] The punicic acid may be a substantially pure single chemical compound or a mixture of one or more punicic acid compounds as defined above. The term "substantially pure" means having a purity of at least 90% by weight, typically at least 95% by weight, such as at least 98% and more, typically about 99% and 100% by weight. According to certain typical embodiments, the punicic acid is in the form of an extract obtained from pomegranate seed oil, either directly or following one or more steps of purification. According to other embodiments, the punicic acid is produced synthetically.

[0061] As used herein, the term "therapeutically effective amount" refers to the amount of pomegranate seed oil or punicic acid that is effective in preventing, reducing further progression or treating a neurodegenerative disease according to the teachings of the invention. The therapeutically effective amount can be administered at a single dose or at multiple doses.

[0062] As used herein, the term "emulsion" refers to a mixture of two immiscible liquids wherein one liquid forms a continuous phase within which droplets of the other liquid are dispersed as a discontinuous phase. In oil in water emulsions, droplets of oil are dispersed in the aqueous phase.

[0063] In the context of the present invention, the principle oil component of the emulsion is the therapeutic active agent—i.e. pomegranate oil and/or fractions thereof. According to certain embodiments, the pomegranate oil emulsion of the present invention is oil in water emulation having an average droplet size of below 1.0 micron. According to certain typical embodiments, the emulsion has an average droplet size of from about 30 nm to 500 nm.

[0064] According to one aspect, the present invention provides a method of preventing and/or treating a neurodegenerative disease, comprising administering to a subject having or at risk of developing a neurodegenerative disease a therapeutically effective amount of pomegranate seed oil or a fraction thereof. According to certain typical embodiments, the subject is human.

[0065] Protein oxidation, one of a number of brain biomarkers of oxidative stress, is increased in several age-related neurodegenerative disorders or animal models thereof, including Alzheimer's disease, Huntington's disease, prion disorders, such as Creutzfeldt-Jakob disease, and alpha-synuclein disorders, such as Parkinson's disease and frontotemporal dementia. Each of these neurodegenerative disorders is associated with aggregated proteins in brain. However, the relationship among protein oxidation, protein aggregation, and neurodegeneration remain unclear. The present invention now shows that administering pomegranate oil in in vitro and in vivo models of neurodegenerative disease results in prolonged cell survival and postponed disease progression.

[0066] According to certain embodiments, the neurodegenerative disease is selected from the group consisting of Creutzfeldt-Jacob disease (CJD), multiple sclerosis (MS), Alzheimer disease, Parkinson disease, and amyotrophic lateral sclerosis (ALS). Each possibility represents a separate embodiment of the present invention.

[0067] Creutzfeldt-Jakob disease (CJD) is a rare neurodegenerative fatal disorder. It affects about one person in every one million people per year worldwide. CJD usually appears in later life and runs a rapid course. CJD can be present in sporadic or transmissible genetic etiologies, the later resulting from pathogenic mutations in the prion protein-encoding (PRNP) gene. The E200K PrP mutation is the most prevalent in the world and is common in Israel in Jews of Libyan origin. About 15-20 patients each year are diagnosed in Israel with CJD, mostly of the genetic form (Meiner Z. et al., 2011, J Neurol 258:255-262). Each genetic CJD patient represents a large family of healthy carriers, to which preventive treatment is required. Preventive treatment is also required for individuals at risk of transmissible prion disease by contamination, as was the case in the "mad cow" disease epidemic. Inventors of the present invention and co-workers have recently published mouse model that expresses the corresponding mutant PrP form of the protein and present spontaneous and progressive neurodegenerative disease from 5 month of age until sacrificed at about 1 year, when observed to be too sick to reach food and water (Friedman-Levi Y. et al., 2011. Plos Phatog 7(11): e1002350). Mice accumulate aggregated and oxidized PrP^{Sc} in their brains in an age dependent manner. These mice were specifically generated for preventive treatment experiments, since treatment may commence when the mice are young and healthy (3 months old in preliminary experiments) or when disease is first diagnosed (5-6 months of age).

[0068] Multiple sclerosis (MS) is a chronic relapsing, multifocal inflammatory disorder of the central nervous system that leads to focal demyelination and scarring of the brain. It is a frequent disease which begins during early to middle adulthood. Multiple sclerosis (MS) is the most common neurological disorder leading to permanent disability in young adults in the developed world. MS is traditionally referred to as an autoimmune inflammatory disease. However, it is becoming increasingly evident that axonal and neuronal degeneration also occur, already at the earliest stages of the disease. This neuronal degradation may be dependent or independent of the inflammation processes. In addition, it is the progressive neurodegeneration which determines the degree of accumulating clinical disabilities. Therefore. MS may be considered as a neurodegenerative disorder. Experimental autoimmune encephalomyelitis, also named Experimental Allergic Encephalomyelitis (EAE) is an animal model of brain inflammation. It is an inflammatory demyelinating disease of the central nervous system (CNS). It is mostly used with rodents and is widely studied as an animal model of the human CNS demyelinating diseases, including the diseases MS and acute disseminated encephalomyelitis (ADEM). EAE is a well established model of MS and has been used in the screening of numerous candidate treatments, including Copaxon. Ten days after immunization of naïve mice with a mixture of Myclin Oligodendrocyte Glycoprotein (MOG)-CFA-Pertussis toxin, mice present severe clinical disease and demyelination, both parameters that can be easily measured by clinical observation and pathology. In addition, the disease is fast and short (10 days from induction and 32 weeks of follow up thereafter for the acute phase). Moreover, long term follow up of the mice presents relapsing remitting signs that can also be followed up after treatment.

[0069] A number of incurable, ageing-related or degenerative diseases have been linked to a generic and fundamental pathogenic process of protein or peptide misfolding and aggregation called "amyloidosis". These Include Alzheimer's. Parkinson's and Huntington's diseases. The amyloid deposits present in these diseases consist of particular peptides that are characteristic for each of these diseases, but regardless of their sequence the amyloid fibrils have a characteristic β-sheet structure and share a common aggregation pathway. In each disease, a specific protein or peptide misfolds, adopts β-sheet structure and oligomerize to form soluble aggregation intermediates en route to fibril formation ultimately forming insoluble amyloid fibers, plaques or inclusions. These insoluble forms of the aggregated protein or peptide form by the intermolecular association of β-strands into β-sheets. Recent evidences suggest that the soluble amyloid oligomers may be the principal cause of neurotoxicity. However, the precise molecular

mechanism by which this generic process of protein/peptide misfolding and aggregation is linked to the progressive degeneration of affected tissues is unclear. In some cases, including many of the systemic amyloid-related diseases, it is thought that the sheer mass of insoluble protein or peptide simply overwhelms the affected tissues, ultimately leading to acute organ failure. In other cases, including most of the neurodegenerative diseases listed above, the symptoms of disease develop with the appearance of only very small aggregates. Therefore, it has been suggested that the insoluble deposits are inherently toxic and might cause the progressive destruction of cells, for example by causing inflammation and oxidative stress, or by directly interfering with cell membranes or other cellular components or processes.

[0070] According to certain embodiments, the methods of the present invention further comprise a step of classifying the human subject as being in need of prevention or treatment of a neurodegenerative disease before administering the pomegranate oil or punicic acid fraction thereof.

[0071] It is always desirable to detect diseases early in their progress. Early detection enables early treatment which has generally been proven to yield a higher success rate in treating various diseases. Recently, it has been discovered that analyzing peoples' eyes, and in particular the lenses of the eyes, can yield indications of various types of neurodegenerative diseases. For example, measurements taken of light scattering within the eye has been shown to provide useful diagnostic information to detect and monitor the progress of neurodegenerative diseases, particularly those involving amyloidosis such as Alzheimer's disease (for example, U.S. Patent Application Publication No. 2008/0088795 and U.S. Pat. No. 7,107,092). In addition, changes in the brain measured with MRI and PET scans, ELISA assays, and diffraction-enhanced imaging (DEI), alone or combined with memory tests and detection of risk proteins in body fluids, may also lead to earlier and more accurate diagnosis of Alzheimer's.

[0072] Classifying a subject as being susceptible to CID can be performed by screening for mutations in the PRNP gene, particularly for the E200K PrP mutation. As discussed above, this mutation is highly prevalent in Israel and identifying and treating carrier subjects is highly desirable.

[0073] According to certain embodiments, the method of the present invention comprises administering to the subject in need thereof pomegranate seed oil. According to other embodiments, the method comprises administering punicic acid. According to certain embodiments, the punicic acid is in its free acid form. Pomegranate oil can be produced by any method as is known to a person skilled in the art.

[0074] The pomegranate seed oil, punicic acid, salts or esters thereof can be administered per w, or within a formulation as is known to a person skilled in the art.

[0075] It is well established that during the metabolism of lipid molecules such as punicic acid, a great part of it is metabolized in the liver soon after its administration. It therefore highly desired to enhance the time of such molecules in the circulation as well as their distribution in tissues other than liver.

[0076] According to some embodiments, the present invention provides the use of a therapeutically effective amount of pomegranate seed oil or a fraction thereof for preventing and/or treating a neurodegenerative disease Unexpectedly, the present invention now shows that oral administration of pomegranate oil formulated to form sub-micron or nanoemulsion is significantly more effective in reducing the clinical disease symptom in mice induced for EAE compared to oral administration of the pomegranate oil per se.

[0077] Thus, according to certain embodiments, the method of the present invention comprises administering the pomegranate oil, punicic acid, salts or esters thereof in a form of emulsion, particularly oil-in-water emulsion.

[0078] An emulsion is defined as a heterogeneous system, consisting of at least two immiscible liquids or phases in the presence of surface active materials such as surfactants or polymers. Emulsions are thermodynamically unstable. The emulsion consists of droplets of a dispersed phase in the continuous phase. Simple emulsions are classified as water in oil (W/O) or oil in water (O/W) depending on which phase constitutes the disperse phase. An important aspect in the preparation of emulsions from both fundamental and technological points of view is to obtain a desired droplet size and a narrow size distribution.

[0079] An oil-in-water emulsion is a dispersion of oil droplets or colloidal particles in an aqueous medium, with the colloid particles having an oily core surrounded by an interfacial film of emulsifiers and surface acting agents or surfactants. According to the teachings of the present invention, the principle ingredient of the oil component of the emulsion is the therapeutic agent, i.e. the pomegranate oil and/or fractions thereof.

[0080] Any emulsifier and/or surfactant that do not interfere with the activity of the pomegranate oil or fraction thereof and with the biocompatible characteristics of the emulsion can be used according to the teachings of the present invention. Furthermore, a surfactant which can solubilize the oil within micelles is also suitable for the delivery system.

[0081] According to certain embodiments, the emulsifier is one or more selected from the group consisting of polyoxyethylene polyoxypropylene copolymer (commercial name: Poloxamer™), polyethylene glycol alkyl ether (commercial name: Brij™), polyoxyethylene sorbitan triglyceride ester (commercial name: Tween™), sorbitan fatty acid ester (commercial name: Span™), transesterification product of natural vegetable oil triglyceride and polyalkylene polyol (commercial name: Labrafil™, Labrasol™), glycerol fatty acid ester (commercial name: Plurol™oleique). Polyoxyl 40 Hydrogenated Castor Oil (commercial name: Cremophor RH 40): vitamin E polyethyleneglycol succinate (Vitamin E polyethylene glycol succinate), lecithin, sodium lauryl sulfate, and bile acid and derivatives thereof.

[0082] According to certain embodiments, the emulsions of the present invention are sub-micron or nano-emulsion. The terms "sub-micron emulsion" and "nano-emulsion" are used herein interchangeably to mean oil droplet size of below 1.0 μm, typically about 0.03 to 1.0 μm, and more typically about 0.05 to 0.4 μm. Thus, a submicron emulsion having droplets of these sizes would be smaller than those of a classical microemulsion, which has droplet sizes of above 0.5 μm.

[0083] With the above-mentioned emulsion droplet size range being typical embodiments of the present invention, it is to be explicitly understood that emulsions of pomegranate oil or fractions thereof having different droplet size ranges are also encompassed within the scope of the present invention, and maybe adapted according to the required target of

action and mode of administration. The emulsion droplet size can be modified as is well known to a person skilled in the art by changing the emulsification process, conditions and emulsion components other than the pomegranate oil or fractions thereof.

[0084] Without wishing to be bound by any specific theory or mechanism of action, the higher efficacy observed for oil in water emulsions of pomegranate oil may be attributed to an improved bioavailability of the emulsified oil. The improved bioavailability may be the result of increased solubility of the active component, improved stability during passage of the gastrointestinal tract, improved absorbance from the gastrointestinal tract, improved uptake by the cells or combinations thereof.

[0085] According to certain typical embodiments, the pomegranate oil, the punicic fraction thereof or an emulsion comprising same is formulated for oral or parenteral administration. However, other forms of administration, including transdermal administration and administration via inhalation are also encompassed within the scope of the present invention.

[0086] For oral administration, an effective amount of the pomegranate oil, punicic acid or an emulsion comprising same can be administered in a form selected from, but not limited to, a solid, semi-solid or liquid state. Specific examples include tablet, capsule, powder, granule, solution, suspension and syrup agents.

[0087] Administering the pomegranate oil, punicic acid, salts or esters thereof in a solid dosage form is highly desirable in terms of subject convenience and compliance. Capsules for delivery into the gastrointestinal tract are known in the art, and include capsules for oral or rectal administration. Typically, the capsules are so designed as to be dissolved in the water environment of the gastrointestinal tract. As disclosed hereinabove, the present invention now shows that administrating the pomegranate oil, derivatives, salts or esters thereof as a sub-micron or nano-sized emulsion increases its efficacy; however, the water phase of the emulsion interrupt with the formation of water-soluble capsules.

[0088] Self-emulsifying drug delivery systems (SEDDS) are defined as isotropic mixtures of oils, surfactants and cosolvents. Self-emulsifying formulations distribute readily in the gastrointestinal tract. The aqueous contents of the stomach and the digestive motility of the intestines provide the required environment and sufficient agitation for the spontaneous formation of emulsions.

[0089] Formulation of pomegranate oil as SEDDS thus enables avoiding the use of aqueous phase and its formulation as capsules for oral delivery.

[0090] The ease of manufacture and scale-up is an additional important advantage of SEDDS compared with other drug delivery systems, such as solid dispersions, liposomes and nanoparticles. SEDDS require very simple and economical manufacturing facilities, such as simple mixer with an agitator and volumetric liquid filling equipment for large-scale manufacturing.

[0091] According to certain embodiments, the pomegranate seed oil, punicic acid, salts or esters thereof are formulated into self emulsifying drug delivery system (SEDDS). According to typical embodiments, the SEDDS comprises the pomegranate seed oil, punicic acid, salts or esters thereof and at least one of emulsifying agent, a surfactant or a combination thereof.

[0092] Methods of formulation for oral administrations are known to a person skilled in the art. For example, to formulate the therapeutically active pomegranate seed oil, punicic acid fraction thereof or emulsion comprising same into tablets, capsules, powders, granules, solutions or suspensions, the pomegranate oil or punicic acid per se, emulsions comprising same or the self emulsifying composition (SEDDS) described hereinabove is preferably mixed with a binder, a disintegrating agent and/or a lubricant. If necessary, the resultant composition may be mixed with a diluent, a buffer, an infiltrating, agent, a preservative, a penetration enhancer and/or a flavor, using known methods. Examples of the binder include crystalline cellulose, cellulose derivatives, cornstarch, and gelatin. Examples of the disintegrating agent include cornstarch, potato starch, and sodium carboxymethylcellulose. Examples of the lubricant include talc and magnesium stearate. Further, additives such as lactose and mannitol may also be used.

[0093] Alternatively, the pomegranate oil, punicic acid, derivative or salts thereof, emulsions or SEDDS comprising same can be formulated as a nutritional additive, either as a foodstuff or as a nutraceutical supplement.

[0094] Nutraceutical formulations of interest include foods for human use, including health food bars, drinks and drink supplements, and the like. These foods are enhanced by the inclusion of the pomegranate oil or punicic acid according to the teachings of the invention. For example, in the treatment of neurodegenerative diseases, such as Alzheimer's, the normal diet of a patient may be supplemented by a nutraceutical formulation taken on a regular basis. Such nutraceuticals may or may not contain calories.

[0095] The term nutraceutical composition as used herein include food product, foodstuff, dietary supplement, nutritional supplement or a supplement composition for a food product or a foodstuff. Thus, in another embodiment the present invention relates to a nutraceutical wherein the nutraceutical is a food product, foodstuff, dietary supplement, nutritional supplement or a supplement composition for a food product or a foodstuff.

[0096] In certain embodiments, the nutraceutical formulation may further comprise curcumin, tea catechins including EGCG, L-theanines, and resveratrol or sulforaphane, found in high concentrations in broccoli.

[0097] For parenteral administration, the pomegranate oil or the punicic acid fraction thereof may be formulated for hypodermically, intracutaneously, intravenously or intramuscularly injection, as described, for example in Remington's Pharmaceutical Sciences, Mack Publishing Company. Philadelphia. PA, 17th ed. (1985). Medicinal drugs for such injection may be prepared by dissolving, suspending or emulsifying the pomegranate oil or punicic acid of the invention into an aqueous or non-aqueous solvent such as vegetable oil, glyceride of synthetic resin acid, ester of higher fatty acid, or propylene glycol by a known method. If desired, additives such as a solubilizing agent, an osmoregulating agent, an emulsifier, a stabilizer, or a preservative may also be added. For formulating into suspensions, syrups or elixirs, a pharmaceutically suitable solvent may be used.

[0098] Still alternatively, the pomegranate oil, punicic acid fraction thereof or emulsion comprising same can be administered in the form of an aerosol or inhalant prepared by charging the pomegranate oil or punicic acid in the form of a liquid or fine powder, together with a gaseous or liquid spraying agent and, if necessary, a known auxiliary agent

such as an inflating agent, into a non-pressurized container such as an aerosol container or a nebulizer. A pressurized gas of, for example, dichlorofluoromethane, propane or nitrogen may be used as the spraying agent.

[0099] The pomegranate oil or punicic acid compound of the present invention may also be used together with an additional compound having other pharmaceutically suitable activity to prepare a medicinal drug. According to certain embodiments, the additional compounds have diverse antioxidant mechanism.

[0100] According to certain embodiments, the antioxidant is selected from the group consisting of synthetic antioxidants or natural ones including, but not limited to, Sulforaphane. Curcumin and Green tea catechins.

[0101] Sulforaphane is found in high concentrations in broccoli. It is both an anti-oxidant and a potent monofunctional inducer of detoxification enzymes in xenobiotic metabolism. Sulforaphane was shown to protect brains from dopaminergic cell death and from damage produced by intracerebral hemorrhage.

[0102] Curcumin is the principal curcuminoid of the popular Indian spice turmeric. It is a free radical scavenger and hydrogen donor and also binds metals, particularly iron and copper. Curcumin is remarkably non-toxic and exhibits great promise as a therapeutic agent. In a transgenic mouse model of Alzheimer's disease, curcumin was shown to reduce plaque burden and overall inflammation, an effect that may be attributed to the highly specific binding of curcumin to beta-amyloid.

[0103] Green tea catechins are the cardinal antioxidative ingredient in the green tea extracts epigallocatechin gallate (EGCG), comprising 20 times more antioxidant-active ingredients than Vitamin C. The antioxidative activity of catechins results from their ability to chelate metal ions, scavenge reactive oxygen species (ROS) and inhibit lipid peroxidation. Catechins have been suggested to suppress processes involved in the pathogenesis of Alzheimer's disease, including amyloid-β production and toxicity.

[0104] The dosage and ratios of the compound(s) of the invention administered via each of the above-described such formulations will vary depending upon known factors, such as the physiological characteristics of the particular composition and its mode and route of administration; the age, health and weight of the recipient, the nature and extent of the symptoms; the kind of concurrent treatment: the frequency of treatment, and the effect desired which can be determined by the expert in the field with normal trials, or with the usual considerations regarding the formulation of a pharmaceutical or nutraceutical composition.

[0105] The following examples are presented in order to more fully illustrate some embodiments of the invention. They should, in no way be construed, however, as limiting the broad scope of the invention. One skilled in the art can readily devise many variations and modifications of the principles disclosed herein without departing from the scope of the invention.

EXAMPLES

Example 1: Effect of Pomegranate Seed Oil and Punicic Acid on the Survival of Fibroblast Isolated from TgMHu2ME199K Mice

[0106] A transgenic model of genetic prion disease linked to the E200K mutation, which exerts spontaneous and transmissible prion disease has been previously reported (Friedman-Levi Y, et al., 2011. ibid). The transgenic mice line, denominated TgMHu2ME199K, express a chimeric mouse/human PrP. Disease related PrP accumulates intraneuronally in the brains of these mice in an age dependent manner. The transgenic mice also present other features of the diseased form of PrP, PrP^{Sc}, such as Proteinase K (PK) resistance and oxidation of methionine residues.

[0107] Primary fibroblasts isolated from 13 days gestation fetuses of TgMHu2ME199K are considered of primary nature: however they preserve the ability to divide for several generations. These fibroblasts were shown to be extremely sensitive to cooper induced oxidation stress (Canello et al., 2012. Neurobiology of Disease 45(3):1010-7).

[0108] The isolated fibroblasts were cultured in the presence of various pomegranate oil batches or punicic acid for 24 h: then stress was induced by the addition of copper for additional 24 h. Subsequently, cell survival was established by the MTT method. FIG. **1** shows that pomegranate oil and punicic acid significantly inhibited the toxic effect of cooper, as measured by elevated percentage of cell survival.

Example 2: Preparation of Oil-In-Water Emulsions of Pomegranate Oil (O/W PMO Emulsions)

A. Preparation of O/W Pomegranate Oil Emulsion by Sonication.

[0109] 1.56 gr of Pomegranate oil, 0.65 gr of Tween 80 and 0.39 gr of ALDO-MO (glyceryl monooleate) were mixed by a magnetic stirrer for 20 min.

[0110] 2.5 gr from the above mixture was added to 0.277 gr of glycerol and mixed with magnetic stirrer for 15 min. 2 gr of the glycerol mixture was then added drop wise to 8 gr of deionized water. After vortexing for 30-60 seconds, a crude white emulsion, with micron sized droplets was obtained. At the second stage 10 gr of this crude emulsion were sonicated by a horn sonicator for 10 min (with the use of ice-water cooling bath).

Components Concentrations at the Final Emulsion.

[0111]

10.8%	Pomegranate oil
4.5%	Tween 80
2.7%	glyceryl monooleate (ALDO-MO)
2%	Glycerol
80%	Water

[0112] The O/W nanoemulsion droplets size was 188 nm (Z Average: peak 1: 1001 nm, 40%: peak 2: 394 nm, 60%: polydispersity index (Pdi): 0.250 (measured by dynamic light scattering (DLS)).

[0113] The same preparation but with larger quantities was preformed, 50 gr of the crude emulsion were sonicated by horn sonicator for 10 and 30 min.

The O/W emulsion droplets size was:

[0114] For 10 min sonication: 1676 nm (Z Average), peak 1: 204 nm, 100%; Pdi: 0.123.

[0115] For 30 min sonication: 161 nm (Z Average), peak 1: 174 nm, 100%: Pdi: 0.141 (all measured by DLS).

B. Preparation of O/W Pomegranate Oil Emulsion by Homogenization with Homogenizer

[0116] 7.8 gr of pomegranate oil, 3.25 gr of Tween 80 and 1.95 gr of glyceryl monooleate (ALDO-MO) were mixed by a magnetic stirrer for 20 min. 12.5 gr from the above mixture was added to 1.385 gr of glycerol and mixed with magnetic stirrer for 15 min. 10 gr of the glycerol mixture was then added drop wise to 40 gr of deionized water. After vortexing for 30-60 seconds a crude white emulsion with micron sized droplets was obtained. At the second stage 50 gr of this crude emulsion were homogenized by homogenizer for 10, 20 or 30 min.

Components Concentrations at the Final Emulsion:

[0117]

10.8%	Pomegranate oil
4.5%	Tween 80
2.7%	glyceryl monooleate
2%	Water

The O/W emulsion droplets size was:

[0118] For 10 min homogenization: 204 nm (Z Average: peak 1: 446 nm, 62.5%: peak 2: 64 nm, 36.7%; peak 3: 5153 nm, 0.4%: Pdi: 0.342.

[0119] For 20 min homogenization: 185 nm (Z Avenge: peak 1: 210 nm, 66.5%; peak 2: 1131 nm, 30.2%; peak 3: 4274 nm, 3.3%; Pdi: 0.244.

[0120] For 30 min homogenization: 205 nm (Z Average: peak 1: 96 nm, 36.6%; peak 2: 510 nm, 60.3%: peak 3: 4960 nm, 3.1%; Pdi: 0.319 (all measured by DLS).

C. Preparation of O/W Pomegranate Oil Emulsion by Homogenization with a High Pressure-Homogenizer (Microfluidizer)

[0121] 31.2 gr of Pomegranate oil, 8 gr of Tween 80 and 5.5 gr of glyceryl monooleate (ALDO-MO) were mixed by a magnetic stirrer for 20 mm, 42 gr from the above mixture was added to 5.5 gr of glycerol and mixed with magnetic stirrer for 15 min. 44 gr of the glycerol mixture was then added drop wise to 56 gr of deionized water. After vortexing for 30-60 seconds a crude white emulsion with micron sized droplets was obtained. At the second stage 100 gr of this crude emulsion were homogenized by high pressure homogenizer.

Components Concentrations at the Final Emulsion:

[0122]

27.13%	Pomegranate oil
6.96%	Tween 80
4.81%	glyceryl monooleate (ALDO-MO)
5.1%	Glycerol
56%	Water

The O/W emulsion droplets size was:

[0123] For 5 homogenization cycle repeats (runs): 156 nm (Z Average: peak 1: 172 nm, 96%: peak 2: 2023 nm, 3%; peak 3: 4706 nm, 1%; Pdi: 0.167.

[0124] For 20 homogenization: cycle repeats: 136 nm (Z Avenge: peak 1: 36 nm, 100%: Pdi: 0.09.

[0125] For sterilization, the obtained emulsion after 20 homogenization repeats was filtered by 0.22p filter.

D. Preparation of O/W Pomegranate Oil Emulsion by Sonication with Tween 80 and Span 80 as Surfactants.

[0126] First, an oil phase was prepared by dissolving 0.25 gr of span 80 in 4.75 gr of PMO using a magnetic stirrer for 10 min. An aqueous phase was prepared by dissolving 2.25 gr tween 80 in 42.75 gr deionized water using also a magnetic stirrer for 10 min. Crude O/W emulsion was prepared by mixing the oil phase with the aqueous phase. At the second stage 50 gr of this crude emulsion were sonicated by a horn sonicator for 15 min (with the use of ice-water cooling bath).

Components Concentrations at the Final Emulsion:

[0127]

9.5%	Pomegranates oil
4.5%	Tween 80
0.5%	Span 80
85.5%	Water

The O/W emulsion droplets size was:

[0128] For 15 min sonication: Z Average 141 nm (peak 1: 135 nm, 100%: Pdi: 0.177

E. Preparation of O/W Pomegranate Oil Emulsion by Homogenizer with Lecithin

[0129] Aqueous phase of the emulsion was prepared by mixing 0.5 g of lecithin (Phospholipon 50) and 9 ml of deionized water, followed by stirring at room temperature with a magnetic stirrer for 15 min. To form the nanoemulsion, 0.5 g of pomegranate oil (PMO) was added to the lecithin solution while homogenizing with homogenizer for 10 min.

Components Concentrations at the Final Emulsion:

[0130]

5%	Pomegranate oil
5%	Lecithin
90%	Water

The O/W emulsion droplets size was:

[0131] For 10 min homogenization: 2×4 nm (Z Average: peak 1: 431 nm, 81% peak 2: 74 nm, 19% Pdi: 0.258.

F. Preparation of Pomegranate Oil Self Emulsifying Drug Delivery System (SEDDS)—Option I

[0132] 0.5 gr of Pomegranate oil, 0.5 gr of Tween 80 and 2.5 gr of glyceryl monooleate (ALDO-MO) were mixed by a magnetic stirrer for 20 min. The above mixture was added to 0.35 gr of glycerol and mixed with magnetic stirrer for 15 min. 10 μl of the glycerol mixture was then added to 3 ml of deionized water. After vortexing for 30-60 seconds an emulsion with sub-micron sized droplets was obtained.

Components Concentrations at the SEDDS:

[0133]

12.98%	Pomegranate oil
25.97%	Tween 80
55.94%	glyceryl monooleate (ALDO-MO)
9.09%	Glycerol

The O/W nanoemulsion droplets size was:

[0134] Z Average 415 nm, peak 1: 260 nm, 25%: peak 2: 33 nm, 75%: Pdi: 0.541.

G. Preparation of Pomegranate Oil Self Emulsifying Drug Delivery System, (SEDDS)—Option II

[0135] 250 mg of Pomegranates oil, 300 mg of Tween 80 and 400 mg of Cremophor RH 40 were mixed by a magnetic stirrer for 20 min. 10 μl of the mixture was then added to 3 ml of deionized water. After vortexing for 30-60 seconds an emulsion with nano-sized droplets was obtained.

Components Concentrations at the SEDDS:

[0136]

26.3%	Pomegranate oil
31.57%	Tween 80
42.13%	Cremophor RH 40

The O/W nanoemulsion droplets size was:

[0137] 101 nm Z Average, peak 1: 35 nm, 92.5%; peak 2: 334 nm, 7.1%: peak 3: 4796 nm, 0.4%: Pdi: 0.403.

H. Preparation of Pomegranate Oil Self Emulsifying Drug, Delivery System (SEDDS)—Option III

[0138] 250 mg of Pomegranate oil, 350 mg of Tween 80, 155 mg of Span 80 and 50 μl of ethanol were mixed by a magnetic stirrer for 20 min. 10 μl of the mixture was then added to 3 ml of deionized water. After vortexing for 30-60 seconds an emulsion with nano-sized droplets was obtained.

Components Concentrations at the SEDDS:

[0139]

31.46%	Pomegranates oil
44%	Tween 80
19.5%	Span 80
4.97%	Ethanol

The O/W nanoemulsion droplets size was:

[0140] 224 nm Z Average, peak 1: 18 nm, 100%: Pdi: 0.389.

Example 3: Effect of Pomegranate Seed Oil Administered in the Food on the Disease Progression in EAE Induced Mice

[0141] Groups of ten naïve C57 black mice were induced for EAE as follows: mice were injected subcutaneously with a mixture of 250 micrograms of MOG35-55 in complete Freud's adjuvant. Mice were supplemented with 200 nanograms of pertussis toxin given intraperitoneally at the day of inoculation and 48 hrs later. Evaluation of clinical signs was done by two different persons blindly as previously described (Pollak Y. et al., J Neuroimmunol 2000, 104(1): 31-36) Scores of disease severity were as follows: [1]: Hind limbs weakness [2] Hind limb partial paralysis [3] Full paralysis in one limb [4] Full hind limb paralysis [5] Death. Control groups were administrated (from day zero) regular rodent food, while the experimental groups received food enriched for pomegranate oil containing 60% punicic acid. The pomegranate oil was administered at a concentration of 25 ml oil/Kg food or at three-time higher close of 75 ml oil/Kg food. Mice had access to food at libido. Mice were followed for signs of disease and then scored for several weeks until most of then returned to normal condition. FIG. **2** describes the development of disease in the distinct groups of mice. The figure shows that following the administration of two different concentrations of pomegranate oil in the mice food, disease appeared at milder scores which returned to basic levels much faster than in the untreated group.

Example 4: Effect of Emulsified Pomegranate Seed Oil Administered by Gavage on the Disease Progression in EAE Induced Mice

[0142] Mice were induced for EAE and evaluated for EAE clinical signed as described in Example 2 hereinabove. Number of mice was 8-10 in each group.

[0143] Two emulsion batches prepared as described in Example 2A hereinabove comprising 10.8% pomegranate oil were used. One batch (designated "Batch 1") comprised oil particles at an average size of 150 nm while another batch (designated "Batch 2") comprised oil particles at an average size of 80 nm. Batch 1 was kept at 4° C. in a regular bottle that was open every day to retrieve the required administration dose. Batch 2 was kept in nitrogeneted ampoules, each containing one-time dose, 200 μl of the emulsions were administered by gavage 5 times a week. All preparations (control and emulsified pomegranate oil) were administered to the mice from the first day of the experiment. Mice receiving food with no addition served as control.

[0144] FIG. **3** shows the disease development in the distinct groups of mice. The figure shows that similarly to the results obtained with pomegranate seed oil per se, disease appeared at milder scores and returned to basic levels much faster than in the untreated group. However, these effects were obtained with emulsified pomegranate oil concentration of about one tenth of the pomegranate oil concentration required when the oil per se was given in the mice food.

[0145] It is further noticeable from FIG. **3** that there is no significant difference in the effect obtained with Batch 1 and Batch 2, indicating that the pomegranate oil within the emulsion is highly stable and not easily susceptible to oxidation.

[0146] In an additional experiment, pomegranate oil emulsion prepared as described in Example 2C above (employing high pressure homogenization, designated "batch 4") was administered to the EAE induced mice. The emulsion was administered at its initial concentration of 27% pomegranate oil and in two diluted forms of pomegranate oil concentration of 5.4% and 1.35%, 100 μl of each emulsion concentration was administered by gavage 5 times a week. As is shown in FIG. **7**, administration of pomegranate oil emulsion delayed the appearance of the disease symptoms, reduced their severity and enhanced the mice recovery. It is to be emphasized that the effect of pomegranate oil was observed also at the lowest concentration examined (1.35% of pomegranate oil). The dose of 100 μl of 1.35% pomegranate oil emulsion per mice is equivalent to a dose of 1.2 ml for a human weighing 60 Kg. This amount can be easily taken by a human subject on a daily basis as required. It is to be further noted that the high pressure homogenization is an efficient method for large scale commercial preparation of emulsions, providing emulsions with the desired range of droplet size.

Example 5: Specificity of Pomegranate Oil

[0147] To specificity of the effect of pomegranate oil was further examined using an emulsion with an oil phase different from the pomegranate oil. The control emulsion contained Soy bean oil as the oil phase. The effect of pomegranate oil on the development of EAE as described in Example 2 hereinabove was thus compared to the effect of Soy bean oil. In addition to batch 2 of the pomegranate oil emulsion described above additional batch, designated “batch 3” was also administered. Batch 3 was also prepared as described in Example 2A with shorter sonication time resulting in a larger droplet size of Z average of about 300 μm. As clearly shown in FIG. **4**, no inhibiting activity of EAE progression was observed for emulsion with Soy bean oil, while both batch 2 and batch 3 of the emulsified pomegranate oil postponed the appearance of the disease signs and significantly reduced its severity. It is thus concluded that punicic acid is the principle active ingredient in the pomegranate oil.

[0148] In an equivalent experiment, administration of the emulsified pomegranate oil (batch 2) and the emulsified Soy bean oil started only at the 7th day after the induction of EAE. FIG. **5** shows that the emulsified pomegranate oil was highly effective in reducing the disease score also when administered at a later stage of the disease development. No effect was observed for the emulsion containing Soy bean oil. These results clearly demonstrate that intake of pomegranate oil reduced the EAE clinical symptoms.

Example 6: Effect of Pomegranate Oil on Activated T-Cell Infiltration in EAE Induced Mice

[0149] CD3-positive T-cells are considered as activated T-cells, and it is known that these activated cells infiltrate into the central nervous system (CNS) during EAE and MS. The infiltration of the activated T-cells induces inflammation of the central nervous system leading to a cascade of symptoms associated with MS, including demyelination and scarring of the brain. Samples of brain and spinal cord were taken from EAE-induced mice treated with emulsified pomegranate oil (batch 4) or from non-treated mice.

[0150] Paraffin-embedded sections were processed for immunohistochemistry with anti CD3 antibodies (Friedman-Levi Y et al., 2007. J Virol 81: 9942-9949). After incubation with the primary antibody, slides were incubated with the appropriate secondary antibody bound to Horseradish peroxidase (HRP) and developed with an HRP substrate. FIG. **8** shows that in samples obtained from untreated mice CD3-positive cells infiltrated into the tissue. In contrast, in the treated samples (0.8 and 13.5% pomegranate oil) the positive cells remained close to the blood vessels, meaning that the activated T-cells were secreted from the blood into the immediate surroundings, but did not pass the second membrane that separate blood vessels from the CNS tissue. These results demonstrate that oral administration of pomegranate oil significantly delayed the appearance of the deleterious MS phenomenon associated with EAE and MS.

Example 7: Histochemical Analysis

[0151] Luxol fast blue (LFB) stain is a commonly used stain to observe myclin under light microscopy. LFB is thus used to detect demyelination in the central nervous system (CNS). Spinal cord samples from naïve mice and from EAE-induced mice non-treated or treated with emulsified pomegranate oil (13.5%) were stained with LFB. The stained pattern of the pomegranate-oil treated mice resembled that of the naïve mice, while part of the blue pattern was missing in the samples obtained from non-treated EAE-induced mice (data no shown). These results indicate that oral administration of emulsified pomegranate oil inhibits demyelination in the brain of EAE-induced model mice.

Example 8: Treatment of TgMHu2ME199K Mice

[0152] Pomegranate oil was administrated to groups of 3 months old TgMHu2ME199K mice in their food as described above for the EAE experiments. In this model the oil was given to the mice for at least six month or as required until the end point of the clinical experiments. The long term administration of the oil had no adverse effect on the mice. Mice were sacrificed at the end of the experiment and brains kept at different conditions for future mechanistic studies. FIG. **6**A shows the clinical results from these experiments, indicating transgenic mice to which pomegranate oil was administered in the food succumb to the spontaneous disease significantly later then mice eating normal food.

[0153] In an additional experiment, emulsified pomegranate oil (batch 2, 10.% oil) was administrated by gavage (200 μl/ml; 5 days a week) to TgMHu2ME199K mice starting at the age of 100 days and up to 150 days. From the 150th day and during 90 days the disease was scored for severity as described in Example 3 hereinabove. The progress of the disease is presented as the average score of treated and untreated groups (FIG. **9**A) or as individual mice reaching score 2, a significant disease marker (FIG. **9**B). A significant difference in the disease progress between the treated and untreated mice was observed.

[0154] FIG. **9**C demonstrates the levels of Proteinase K (PK) resistant PrP, which is a marker of the prion disease, in treated and untreated mice at the time of the experiment termination (the same for both groups). Surprisingly, higher concentration of the aberrantly folded protein (PrPSc) was observed in samples taken from the treated mice. Without wishing to be bound by any specific theory or mechanism of action, these results may imply a neuroprotective effect of the pomegranate oil, which allows the cells to survive for longer time while continuing to produce the toxic protein.

[0155] FIG. **9**D shows staining of the synaptophysin protein in brain cerebellar samples taken from naïve mice, from untreated EAE-induced mice and from EAE-induced mice treated with pomegranate oil emulsion. Paraffin-embedded sections were processed for immunohistochemistry with anti synaptophysin antibodies (Ferrer I 2002. Cerebellum 1: 213-222). After incubation with the primary antibody, slides were incubated with the appropriate secondary antibody bound to Horseradish peroxidase (HRP) and developed with an HRP. As is clearly demonstrated in FIG. **9**D, samples taken from treated mice showed higher staining, closer to the staining shown for naïve mice. Intact synaptophysin is a measure for intact synapses. In neurodegenerative diseases, preservation of intact synapses correlated with preservation of cognitive abilities. Thus, these results show that pomegranate oil can be used for preservation of brain synapses and cognitive abilities of subject afflicted with a neurodegenerative disease such as Parkinson and Alzheimer’s disease.

Example 9: Effect of Pomegranate Oil on TgMHu2ME199K Mice Showing Disease Signs

[0156] In this experiment, TgMHu2ME199K mice already showing disease symptoms (score 1.5 and above) were treated with emulsified pomegranate oil (batch 3). The pomegranate oil emulsion was added to the drinking water at a concentration of 10 g oil emulsion per 100 ml. Individual dose per day under these conditions is equivalent to the gavage administration of 200 μl/ml. FIG. **10** shows that the rate of the disease progression is significantly lower compared to its progress in untreated mice, implying that pomegranate oil is effective also in treating an existing neurodegenerative disease such as CJD. Progress of the disease was measured as the disease score average of all participating subjects (FIG. **10**A), or as the percentage of mice exhibiting change of less than 0.5 score (FIG. **10**B).

[0157] The foregoing description of the specific embodiments will so fully reveal the general nature of the invention that others can, by applying current knowledge, readily modify and/or adapt for various applications such specific embodiments without undue experimentation and without departing from the generic concept, and, therefore, such adaptations and modifications should and are intended to be comprehended within the meaning and range of equivalents of the disclosed embodiments. It is to be understood that the phraseology or terminology employed herein is for the purpose of description and not of limitation. The means, materials, and steps for carrying out various disclosed functions may take a variety of alternative forms without departing from the invention.

What is claimed:

1. A method of treating a condition associated with oxidative stress of components within the central nervous system (CNS), the method comprising administering to a subject in need thereof a therapeutically effective amount of pomegranate seed oil, a fraction thereof, derivatives or salts of same.

2. The method of claim **1**, wherein said method comprises administering the pomegranate seed oil, fraction thereof, derivatives or salts of same as the sole pomegranate-derived component.

3. The method of claim **1**, wherein the condition associated with oxidative stress of components within the central nervous system (CNS) is other than Creutzfeldt-Jacob disease (CJD), multiple sclerosis (MS), Alzheimer disease, Parkinson disease, and amyotrophic lateral sclerosis (ALS).

4. The method of claim **1**, wherein the pomegranate seed oil comprises at least 50% punicic acid.

5. The method of claim **4**, wherein the pomegranate seed oil comprises punicic acid at a concentration selected from the group consisting of at least 60%, at least 80% and at least 95%.

6. The method of claim **1**, wherein the pomegranate seed oil fraction is punicic acid.

7. The method of claim **1**, wherein the pomegranate seed oil, fraction thereof, derivatives or salts of same is administered in a form of an emulsion or in a form of self-emulsifying drug delivery system (SEDDS).

8. The method of claim **7**, wherein the emulsion is an oil in water emulsion, comprising an oil phase comprising pomegranate seed oil, a fraction thereof, derivatives or salts thereof at a concentration of at least 5% (w/w); an emulsifier comprising at least one emulsifying agent; and an aqueous phase.

9. The method of claim **7**, wherein the emulsion is a sub-micron emulsion or nano-emulsion.

10. The method of claim **7**, wherein the SEDDS comprises an oil phase comprising pomegranate seed oil, a fraction thereof, derivatives or salts thereof at a concentration of at least 5% (w/w); at least one emulsifier; and at least one non-aqueous solvent; and wherein said SEDDS is devoid of an aqueous phase.

11. The method of claim **10**, wherein the pomegranate seed oil fraction is punicic acid.

12. The method of claim **10**, wherein the oil phase consists of pomegranate seed oil or a fraction thereof.

13. The method of claim **1**, wherein the subject is human.

14. The method of claim **1**, wherein the pomegranate seed oil, fraction thereof, derivative or salts of same is administered orally in a form selected from a nutraceutical formulation, a functional food, a food additive, or a dietary supplement.

15. The method of claim **7**, wherein the emulsion is administered orally in a form selected from a nutraceutical formulation, a functional food, a food additive, or a dietary supplement.

16. The method of claim **7**, wherein the SEDDS is administered orally in a form selected from a nutraceutical formulation, a functional food, a food additive, or a dietary supplement.

17. The method of claim **16**, wherein the SEDDS is encapsulated in a coating material.

* * * * *
