# Supplementary material for: Non-Categorical Analyses Identify Rotenone-Induced ‘Parkinsonian’ Rats Benefiting from Nano-Emulsified Punicic Acid (Nano-PSO) in a Phenotypically Diverse Population: Implications for Translational Neurodegenerative Therapies
Source: Int J Mol Sci. 2024 Nov 25;25(23):12635. doi: 10.3390/ijms252312635 (PMC11640963; doi:10.3390/ijms252312635)

The influence of individual diversity on the benefit of consuming nanoemulsified punicic acid (Nano-PSO) in a population of rotenone conditioned, parkinsonian albino rats.

Statistical analysis of the project database

Biostatistics

2023-03-09

Content

Introduction....2
Importing Data....2
Statistical analysis....4
Descriptive statistics....4
Principal Components (PCAs)....11
Correlated tables....32
Conclusion....33
Dendograms....34
Annex....43
Treatment comparison....43

Introduction

The results presented in this document are the product of statistical analyses carried out on the data contained in the following three files:

1. *Relative expression 24-01-23 National Polytechnic Institute.xlsx*
2. *HPLC 01.24.23 Mexican Institute of Psychiatry.xlsx*
3. *Miscellaneous Parameters 25-01-23 Biomedical Research Institute.xlsx*

The content of the previous files consisted of several hierarchical contingency tables, where a first row was the treatments, the second row the individuals (rats), a third row some variable (for example, glucose level), etc. This type of structure is not suitable for statistical analysis, at least for most popular computer programs, such as R, SPSS, SAS, STATA, etc.

The proper structure of the data should be like a matrix, where the rows are the individuals, the columns the variables or characteristics of those individuals, and the cell a value of that characteristic (column) for that individual (row).

Thus, after importing the data into the R environment, it was transformed into the appropriate structure to carry out the following statistical analyses:

1. Descriptive statistics.
2. Principal component analysis.
3. Dendograms.
4. Identification of *outliers*.
5. Comparison of treatments.

The latter is presented in an annex.

Importing data

File 1: Miscellaneous Parameters 25-01-23 Institute of Biomedical Research.xlsx

In this file there are 6 tables, labeled as:

1. “TABLE 1 NUMBER OF TH+ NEURONS IN S. nigra”
2. “TABLE 2 a-SYNUCLEIN”
3. “TABLE 3 MDA”
4. “TABLE 4 ANTIOXIDANT ENZYMES IN BLOOD”
5. “TABLE 5 LATENCY”
6. “TABLE 6 BIOCHEMICAL INDICATORS”

File 2: HPLC 01.24.23 Mexican Institute of Psychiatry.xlsx

In this file there are 2 tables, labeled as:

1. “TABLE 2 NEUROTRANSMITERS AND METABOLITES IN Caudate nucleus”
2. “TABLE 3 NEUROTRANSMITERS AND METABOLITES IN S. nigra”

File 3: Relative expression 24-01-23 National Polytechnic Institute.xlsx

In this file there is only 1 table, labeled as:

1. “TABLE 8 GENE RELATIVE EXPRESSION IN Caudate nucleus”

Each of these tables was transformed into a structure called a *dataframe* in the R environment. For example, this last table was transformed to the following dataframe:

Show 10 entries

Search:

	trat	rat	var	valor
1	Ctrl	R2	CAT	0.42042912
2	Ctrl	R3	CAT	0.64574773
3	Ctrl	R4	CAT	0.87144573
4	Ctrl	R5	CAT	0.94502656
5	Ctrl	R6	CAT	0.45829854
6	Ctrl	R7	CAT	0.04320246
7	Ctrl	R8	CAT	0.038847
8	ROT	R2	CAT	0.4990321
9	ROT	R3	CAT	0.36467774
10	ROT	R4	CAT	0.90055109

Showing 1 to 10 of 196 entries

Previous 1 2 3 4 5 … 20 Next

Statistical analysis

To respond to the main objective of the study, descriptive statistical calculations, principal component analysis and hierarchical classification using dendograms were performed, the results of which will be presented below.

Also for complementarity, statistical comparisons of treatments are attached for each of the variables.

Descriptive statistics

Tables 1 to 9 show a red gradient to indicate variation in mean and standard deviation values, faint gradient emphasizes low value and intense gradient high value.

Table 1
Number of TH positive neurons (1000) in S. nigra

Group	Variable	n	Min	max	median	iqr	mean	sd	se
**Ctrl**	Left SNpc	6	7.88	17.32	9.90	2.64	10.96	**3.42**	1.40
**ROT**	Left SNpc	6	0.98	2.91	1.86	1.80	1.92	**0.99**	0.40
**ROT/nano-PSO**	Left SNpc	6	2.67	4.89	3.56	0.79	3.62	**0.78**	0.32
**Nano-PSO/ROT**	Left SNpc	6	4.65	7.01	5.85	1.23	5.81	**0.90**	0.37
**Ctrl**	Right SNpc	6	8.35	12.30	10.00	2.69	10.21	**1.67**	0.68
**ROT**	Right SNpc	6	1.91	2.89	2.30	0.42	2.30	**0.36**	0.15
**ROT/nano-PSO**	Right SNpc	6	1.76	4.17	2.36	1.40	2.76	**1.00**	0.41
**Nano-PSO/ROT**	Right SNpc	6	4.49	6.60	5.84	1.07	5.62	**0.81**	0.33
**Ctrl**	Total	6	17.50	29.14	19.96	3.94	21.17	**4.35**	1.77
**ROT**	Total	6	3.24	5.33	4.21	1.25	4.22	**0.83**	0.34
**ROT/nano-PSO**	Total	6	5.64	7.42	6.29	1.09	6.37	**0.75**	0.31
**Nano-PSO/ROT**	Total	6	10.49	13.02	11.12	1.19	11.43	**0.97**	0.40

Table 2
Concentration of a-synuclein (pg/ml)

Group	Variable	n	min	max	median	iqr	mean	sd	se
**Ctrl**	Caudate nucleus	8	8.19	27.45	21.74	7.79	20.16	**6.76**	2.39
**ROT**	Caudate nucleus	8	205.69	319.08	251.87	37.53	255.59	**35.80**	12.66
**ROT/nano-PSO**	Caudate nucleus	8	13.04	16.76	13.87	1.02	14.12	**1.22**	0.43
**Nano-PSO/ROT**	Caudate nucleus	8	13.04	16.76	13.87	1.02	14.12	**1.22**	0.43
**Ctrl**	S. nigra	8	58.23	72.45	64.87	4.69	65.32	**4.42**	1.56
**ROT**	S. nigra	8	432.74	692.35	592.86	102.48	586.83	**87.17**	30.82
**ROT/nano-PSO**	S. nigra	8	177.46	373.82	228.74	162.25	259.08	**83.95**	29.68
**Nano-PSO/ROT**	S. nigra	8	177.46	373.82	228.74	162.25	259.08	**83.95**	29.68

Table 3
Concentration of MDA (nM)

Group	Variable	n	Min	max	median	iqr	mean	sd	se
**Ctrl**	Caudate nucleus	8	17.43	71.03	27.31	21.07	35.78	**20.05**	7.09
**ROT**	Caudate nucleus	7	193.81	294.41	223.11	73.58	242.41	**43.10**	16.29
**ROT/nano-PSO**	Caudate nucleus	8	16.93	33.24	22.82	5.88	23.73	**5.35**	1.89
**Nano-PSO/ROT**	Caudate nucleus	8	11.53	41.70	25.67	6.66	24.60	**9.54**	3.37
**Ctrl**	S. nigra	8	47.87	158.46	63.98	36.34	76.92	**37.02**	13.09
**ROT**	S. nigra	7	495.73	675.00	541.84	89.25	567.53	**67.39**	25.47
**ROT/nano-PSO**	S. nigra	8	222.23	427.53	367.86	43.20	345.56	**67.67**	23.93
**Nano-PSO/ROT**	S. nigra	8	126.29	300.96	208.61	87.01	216.79	**62.80**	22.20

Table 4
Activity of Antioxidant Enzymes

Group	Variable	n	min	max	median	iqr	mean	sd	se
**Ctrl**	(GPx activity product)NADPH (nmol/ml)	8	74.68	175.72	109.05	52.01	113.59	**36.70**	12.97
**ROT**	(GPx activity product)NADPH (nmol/ml)	8	28.94	114.02	44.42	31.44	55.44	**28.98**	10.25
**ROT/nano-PSO**	(GPx activity product)NADPH (nmol/ml)	8	39.48	134.76	81.51	12.98	80.21	**27.30**	9.65
**Nano-PSO/ROT**	(GPx activity product)NADPH (nmol/ml)	8	17.91	122.88	100.55	69.55	82.75	**43.92**	15.53
**Ctrl**	Catalase specific activity (mU/ml)	8	24.06	55.33	34.54	11.78	38.61	**10.05**	3.55
**ROT**	Catalase specific activity (mU/ml)	8	20.10	27.56	22.63	3.56	22.93	**2.61**	0.92
**ROT/nano-PSO**	Catalase specific activity (mU/ml)	8	25.44	53.44	36.60	14.73	37.76	**9.77**	3.45
**Nano-PSO/ROT**	Catalase specific activity (mU/ml)	8	12.04	76.97	24.41	40.20	35.79	**25.55**	9.03
**Ctrl**	SOD Activity (% inhibition rate)	8	2.40	23.70	3.30	1.73	5.88	**7.28**	2.57
**ROT**	SOD Activity (% inhibition rate)	8	8.80	22.95	16.05	6.53	16.38	**4.99**	1.76
**ROT/nano-PSO**	SOD Activity (% inhibition rate)	8	4.10	27.45	14.10	14.01	15.38	**8.54**	3.02
**Nano-PSO/ROT**	SOD Activity (% inhibition rate)	8	3.30	15.75	6.00	7.38	7.79	**4.92**	1.74

Table 5
Incline beam test (Latency in seconds)

Group	Variable	n	min	max	median	iqr	mean	sd	se
**Ctrl**	21 days	10	7.00	97.00	19.00	19.00	33.00	**34.42**	10.88
**ROT**	21 days	10	96.00	120.00	119.00	8.00	115.20	**7.84**	2.48
**ROT/nano-PSO**	21 days	10	17.00	120.00	120.00	83.25	83.80	**47.29**	14.96
**Nano-PSO/ROT**	21 days	10	10.00	65.00	31.00	29.50	32.10	**18.85**	5.96
**Ctrl**	42 days	10	5.00	120.00	16.50	21.00	28.20	**33.80**	10.69
**ROT**	42 days	10	120.00	120.00	120.00	0.00	120.00	**0.00**	0.00
**ROT/nano-PSO**	42 days	10	7.00	120.00	15.50	7.75	23.60	**34.13**	10.79
**Nano-PSO/ROT**	42 days	10	5.00	120.00	20.50	13.00	30.00	**34.03**	10.76

Table 6
Blood Biochemistry

Group	Variable	n	min	max	median	iqr	mean	sd	se
**Ctrl**	CHOLESTEROL (mmol/L)	8	150.00	165.00	156.50	4.00	156.50	**4.57**	1.61
**ROT**	CHOLESTEROL (mmol/L)	8	150.00	158.00	153.00	4.00	153.50	**3.02**	1.07
**ROT/nano-PSO**	CHOLESTEROL (mmol/L)	8	151.00	166.00	160.00	3.00	159.38	**4.41**	1.56
**Nano-PSO/ROT**	CHOLESTEROL (mmol/L)	8	154.00	163.00	158.50	4.75	158.88	**3.27**	1.16
**Ctrl**	GLUCOSE (mmol/L)	8	76.00	113.00	82.50	2.50	85.50	**11.41**	4.04
**ROT**	GLUCOSE (mmol/L)	8	83.00	129.00	95.50	19.25	99.75	**15.46**	5.47
**ROT/nano-PSO**	GLUCOSE (mmol/L)	8	71.00	94.00	81.50	5.75	80.88	**6.77**	2.39
**Nano-PSO/ROT**	GLUCOSE (mmol/L)	8	77.00	156.00	92.50	12.25	97.38	**25.05**	8.86
**Ctrl**	TRIGLYCERIDES (mmol/L)	8	101.00	272.00	144.00	21.75	151.50	**51.43**	18.18
**ROT**	TRIGLYCERIDES (mmol/L)	8	99.00	150.00	130.00	32.75	129.25	**19.59**	6.93
**ROT/nano-PSO**	TRIGLYCERIDES (mmol/L)	8	107.00	215.00	141.00	35.50	145.25	**35.32**	12.49
**Nano-PSO/ROT**	TRIGLYCERIDES (mmol/L)	8	97.00	188.00	138.00	34.50	135.12	**29.36**	10.38

Table 7
Neurotransmitter and Metabolites in Caudate nucleus

Group	Variable	n	min	max	median	iqr	mean	sd	se
**Ctrl**	5HT	8	0.80	2.46	1.32	0.53	1.39	**0.53**	0.19
**ROT**	5HT	8	1.01	8.49	1.38	0.16	2.25	**2.53**	0.89
**ROT/nano-PSO**	5HT	8	1.05	1.92	1.43	0.30	1.46	**0.27**	0.10
**Nano-PSO/ROT**	5HT	8	0.62	1.78	1.16	0.38	1.18	**0.35**	0.12
**Ctrl**	DA	8	10.48	43.59	31.32	10.04	29.68	**11.13**	3.93
**ROT**	DA	8	20.84	34.50	25.87	6.00	26.53	**4.47**	1.58
**ROT/nano-PSO**	DA	8	9.52	35.97	28.44	10.85	26.08	**8.71**	3.08
**Nano-PSO/ROT**	DA	8	14.41	27.27	21.45	6.58	22.20	**4.55**	1.61
**Ctrl**	DA/DOPA	8	0.34	2.17	1.46	0.84	1.41	**0.70**	0.25
**ROT**	DA/DOPA	8	0.85	2.42	1.13	0.27	1.21	**0.51**	0.18
**ROT/nano-PSO**	DA/DOPA	8	0.34	2.75	0.95	0.51	1.09	**0.73**	0.26
**Nano-PSO/ROT**	DA/DOPA	8	1.04	3.11	1.85	1.29	2.04	**0.80**	0.28
**Ctrl**	DOPAC	8	11.93	69.76	20.72	15.58	26.99	**18.81**	6.65
**ROT**	DOPAC	8	14.25	27.76	24.91	4.47	23.33	**4.71**	1.66
**ROT/nano-PSO**	DOPAC	8	11.44	63.16	27.87	26.09	31.30	**18.97**	6.71
**Nano-PSO/ROT**	DOPAC	8	8.64	20.95	10.32	3.51	11.90	**4.04**	1.43

Table 8
Neurotransmitters and Metabolites in S. nigra

Group	Variable	n	min	max	median	iqr	mean	sd	se
**Ctrl**	5ht	3	1.08	1.99	1.85	0.46	1.64	**0.49**	0.28
**ROT**	5ht	3	1.56	2.17	2.13	0.31	1.95	**0.34**	0.20
**ROT/nano-PSO**	5ht	5	0.36	2.39	1.96	1.52	1.57	**0.93**	0.41
**Nano-PSO/ROT**	5ht	4	2.03	2.81	2.54	0.22	2.48	**0.32**	0.16
**Ctrl**	DA	3	1.16	2.76	1.42	0.80	1.78	**0.86**	0.50
**ROT**	DA	3	0.56	1.14	1.06	0.29	0.92	**0.31**	0.18
**ROT/nano-PSO**	DA	5	0.07	1.17	0.75	0.69	0.67	**0.46**	0.21
**Nano-PSO/ROT**	DA	4	1.96	2.45	2.23	0.31	2.22	**0.22**	0.11
**Ctrl**	DA/DOPA	3	0.07	0.15	0.09	0.04	0.10	**0.05**	0.03
**ROT**	DA/DOPA	3	0.02	0.05	0.04	0.02	0.04	**0.02**	0.01
**ROT/nano-PSO**	DA/DOPA	5	0.01	0.04	0.02	0.01	0.02	**0.01**	0.00
**Nano-PSO/ROT**	DA/DOPA	4	0.03	0.06	0.04	0.01	0.04	**0.01**	0.01
**Ctrl**	DOPAC	3	9.14	29.87	17.67	10.37	18.89	**10.42**	6.01
**ROT**	DOPAC	3	21.34	30.83	29.85	4.75	27.34	**5.22**	3.01
**ROT/nano-PSO**	DOPAC	5	7.15	49.44	28.77	34.34	28.77	**19.27**	8.62
**Nano-PSO/ROT**	DOPAC	4	40.30	68.85	59.78	8.26	57.18	**12.05**	6.03

Table 9
Relative Gene Expression in the Caudate nucleus

Estadísticos descriptivos

Group	Variable	n	min	max	median	iqr	mean	sd	se
**Ctrl**	CAT	7	0.04	0.94	0.46	0.53	0.49	**0.36**	0.14
**ROT**	CAT	7	0.04	0.90	0.36	0.58	0.38	**0.36**	0.14
**ROT/nano-PSO**	CAT	7	0.01	0.02	0.02	0.00	0.02	**0.01**	0.00
**Nano-PSO/ROT**	CAT	7	0.36	0.98	0.92	0.16	0.83	**0.22**	0.08
**Ctrl**	GLUT3	7	0.49	0.94	0.82	0.28	0.73	**0.18**	0.07
**ROT**	GLUT3	7	0.08	0.37	0.16	0.12	0.20	**0.11**	0.04
**ROT/nano-PSO**	GLUT3	7	0.21	0.87	0.63	0.09	0.59	**0.20**	0.07
**Nano-PSO/ROT**	GLUT3	7	0.02	0.76	0.59	0.62	0.40	**0.34**	0.13
**Ctrl**	GLUT4	7	0.01	0.80	0.36	0.37	0.31	**0.28**	0.11
**ROT**	GLUT4	7	0.01	0.89	0.20	0.26	0.26	**0.31**	0.12
**ROT/nano-PSO**	GLUT4	7	0.13	0.81	0.54	0.38	0.49	**0.27**	0.10
**Nano-PSO/ROT**	GLUT4	7	0.00	0.25	0.09	0.15	0.11	**0.10**	0.04
**Ctrl**	GPX1	7	0.00	0.45	0.03	0.16	0.12	**0.17**	0.06
**ROT**	GPX1	7	0.00	0.35	0.00	0.18	0.10	**0.16**	0.06
**ROT/nano-PSO**	GPX1	7	0.29	0.85	0.73	0.15	0.66	**0.18**	0.07
**Nano-PSO/ROT**	GPX1	7	0.24	0.85	0.57	0.18	0.53	**0.21**	0.08
**Ctrl**	SNCA	7	0.00	0.98	0.57	0.69	0.53	**0.42**	0.16
**ROT**	SNCA	7	0.11	0.68	0.20	0.07	0.24	**0.20**	0.08
**ROT/nano-PSO**	SNCA	7	0.17	0.93	0.73	0.46	0.62	**0.30**	0.11
**Nano-PSO/ROT**	SNCA	7	0.04	0.20	0.12	0.05	0.13	**0.05**	0.02
**Ctrl**	SOD	7	0.04	0.94	0.12	0.63	0.38	**0.40**	0.15
**ROT**	SOD	7	0.00	0.35	0.15	0.22	0.14	**0.14**	0.05
**ROT/nano-PSO**	SOD	7	0.00	0.71	0.06	0.25	0.20	**0.28**	0.11
**Nano-PSO/ROT**	SOD	7	0.02	0.66	0.08	0.44	0.26	**0.27**	0.10
**Ctrl**	TH	7	0.49	0.90	0.73	0.28	0.71	**0.17**	0.06
**ROT**	TH	7	0.45	0.90	0.77	0.12	0.76	**0.15**	0.06
**ROT/nano-PSO**	TH	7	0.23	0.64	0.48	0.17	0.49	**0.14**	0.06
**Nano-PSO/ROT**	TH	7	0.22	0.94	0.64	0.32	0.61	**0.26**	0.10

Main Components (PCA)

In contrast to comparing treatments, principal component analysis (PCA) deals with all the variables in each table. PCA transforms a set of correlated variables into a new set of uncorrelated variables. The goal of PCA is to reduce the dimensionality in which the original set of variables is expressed. Normally, if the number of variables is reduced there is no gain in your result. In the identification of individuals, reference is made to the unique sequential number they have.

File 1, Table 1: Mean of TH+ Neurons (10^3)

Although the table has three variables (Left SNpc, Right SNpc and Total), the third of them is the sum of the first two, and we could say that only two are possibly independent. In this case the point scatter plot is as follows.

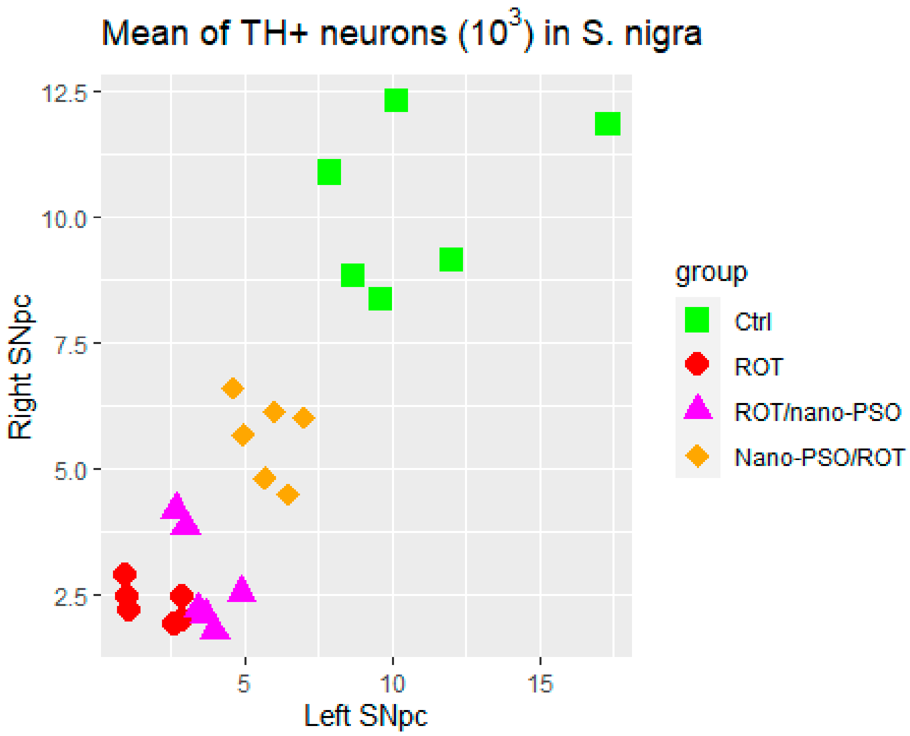

Given the small number of variables, the advantage of principal component analysis is small. The graph of the transformation of the coordinates of the records to the first two components is illustrated in the following graph.

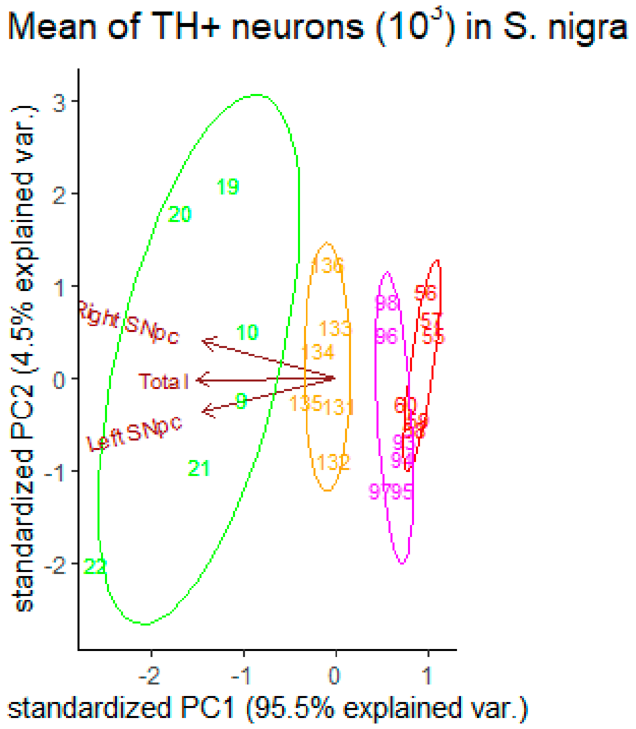

In the graph above we observe that the point clouds of the different groups move almost perpendicular to the axis of the Total variable. Although less precisely, we observe in the scatter plot above that the point clouds of the groups advance perpendicular to the line (which gives the perpendicularity of the family of lines). This is consistent with what is observed in the treatment comparison graph with respect to Table 1 and the total variable. The following table calculates the percentages of intersection of the ellipses.$y = xx + y = k$

Ellipses Intersection Table (Percentages of Areas)

```
##                  Ctrl       ROT  ROT/nano-PSO  Nano-PSO/ROT
## Ctrl                1 0.0000000    0.00000000             0
## ROT                 0 1.0000000    0.06104671             0
## ROT/nano-PSO        0 0.1213536    1.00000000             0
## Nano-PSO/ROT        0 0.0000000    0.00000000             1
## attr(,"class")
## [1] "Overlap"
```

The following table identifies the upper or lower ends of each variable: A minus sign, "-", to the right of the number will indicate that such a value is a lower outlier, while a plus sign, "+", will indicate that it is higher.

Left SNpc		Right SNpc		Total SNpc		trat	id
9.650		8.350	-	18.00		Ctrl	9
8.690		8.810		17.50	-	Ctrl	10
7.880	-	10.870		18.75		Ctrl	19
10.150		12.300	+	22.45		Ctrl	20
12.040		9.130		21.17		Ctrl	21
17.320	+	11.820		29.14	+	Ctrl	22
1.070		2.170		3.24	-	ROT	55
0.980	-	2.890	+	3.87		ROT	56
1.025		2.425		3.45		ROT	57
2.914	+	1.966		4.88		ROT	58
2.650		1.910	-	4.56		ROT	59
2.895		2.435		5.33	+	ROT	60
3.450		2.190		5.64	-	ROT/nano-PSO	93
3.680		2.070		5.75		ROT/nano-PSO	94
3.990		1.760	-	5.75		ROT/nano-PSO	95
3.010		3.830		6.84		ROT/nano-PSO	96
4.890	+	2.530		7.42	+	ROT/nano-PSO	97
2.670	-	4.170	+	6.84		ROT/nano-PSO	98
5.680		4.810		10.49	-	Nano-PSO/ROT	131
6.510		4.490	-	11.00		Nano-PSO/ROT	132
4.980		5.670		10.65		Nano-PSO/ROT	133
6.020		6.130		12.15		Nano-PSO/ROT	134
7.010	+	6.010		13.02	+	Nano-PSO/ROT	135
4.650	-	6.600	+	11.25		Nano-PSO/ROT	136

File 1, Table 2: α -synuclein Concentration (pg/ml)

This table contains two variables related to -synuclein Concentration (pg/ml): "Caudate nucleus" and "S. nigra". As with Table 1, with only two variables, the advantage of PCA is reduced. The point scatterplot is as followsα.

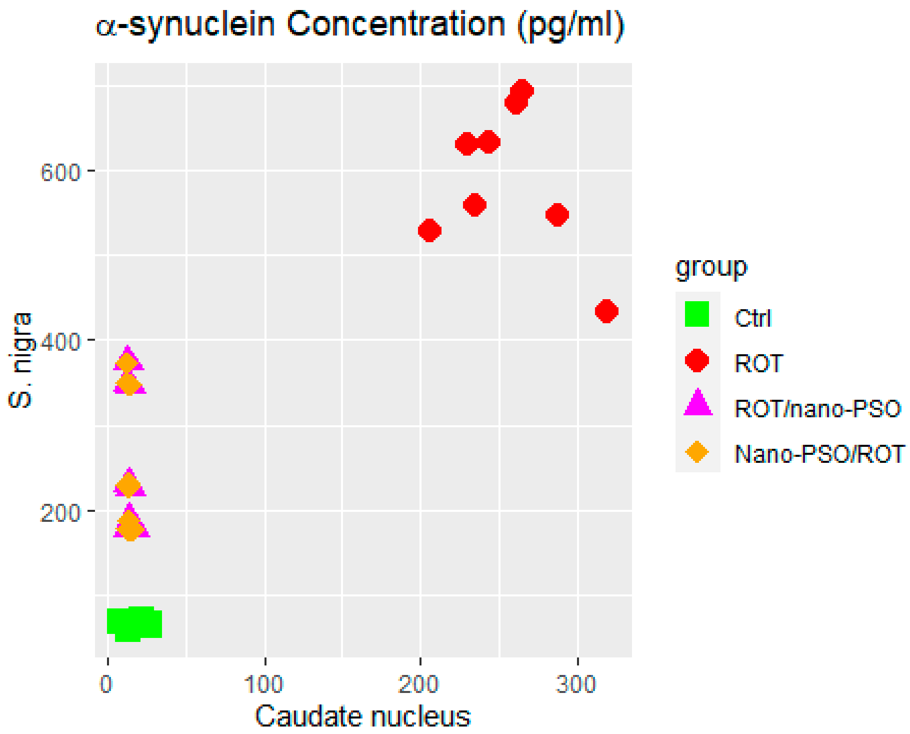

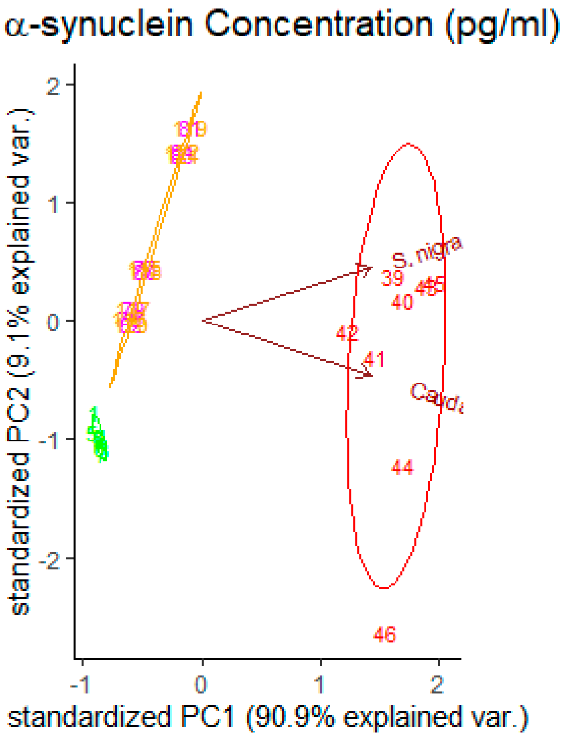

In this case the first main component explains over 90% of the total variance. Reviewing the rotation matrix we observe that:

$$PC_1 = 0.7071\,\text{Caudate nucleus}_z + 0.7071\,\text{S. nigra}_z$$

that is, the first major component is an average of the two standardized variables.

Caudate nucleus		S. nigra		trat	id
8.192161	-	68.97800		Ctrl	1
21.300210		67.24000		Ctrl	2
27.417300		66.74400		Ctrl	3
22.174080		72.45300	+	Ctrl	4
13.188114		58.23124	-	Ctrl	5
23.619835		63.00011		Ctrl	6
27.451732	+	62.94440		Ctrl	7
17.978822		63.00011		Ctrl	8
229.165000		628.72500		ROT	39
243.147000		632.20000		ROT	40
234.408300		556.99400		ROT	41
205.686800	-	527.20900		ROT	42
260.590841		678.42463		ROT	43
287.747471		545.96804		ROT	44
264.923841		692.35110	+	ROT	45
319.082044	+	432.73789	-	ROT	46
13.435380		230.60500		ROT/nano-PSO	77
13.435380		177.98600		ROT/nano-PSO	78
14.309250		188.41000		ROT/nano-PSO	79
14.309250		226.88200		ROT/nano-PSO	80
13.090629		373.82099	+	ROT/nano-PSO	81
16.760255	+	177.45767	-	ROT/nano-PSO	82
14.538982		347.36069		ROT/nano-PSO	83
13.041886	-	350.14598		ROT/nano-PSO	84
13.435380		230.60500		Nano-PSO/ROT	115
13.435380		177.98600		Nano-PSO/ROT	116
14.309250		188.41000		Nano-PSO/ROT	117
14.309250		226.88200		Nano-PSO/ROT	118
13.090629		373.82099	+	Nano-PSO/ROT	119
16.760255	+	177.45767	-	Nano-PSO/ROT	120
14.538982		347.36069		Nano-PSO/ROT	121
13.041886	-	350.14598		Nano-PSO/ROT	122

Ellipses Intersection Table (Percentages of Areas)

```
##                Ctrl  ROT  ROT/nano-PSO  Nano-PSO/ROT
## Ctrl              1    0             0             0
## ROT               0    1             0             0
## ROT/nano-PSO      0    0             1             1
## Nano-PSO/ROT      0    0             1             1
## attr(,"class")
## [1] "Overlap"
```

Being the percentages of intersection 1, it follows that the point clouds of the ROT/nano-PSO and Nano-PSO/ROT groups cannot be differentiated from each other.

Archivo 1,Tabla 3: MDA Concentration (nM)

The table "MDA Concentration (nM)" contains only two variables: "Caudate nucleus" and "S. nigra". From what was observed in the treatment comparison graph, the variable "S. nigra" is differentiating from the groups. The scatter plot is as follows.

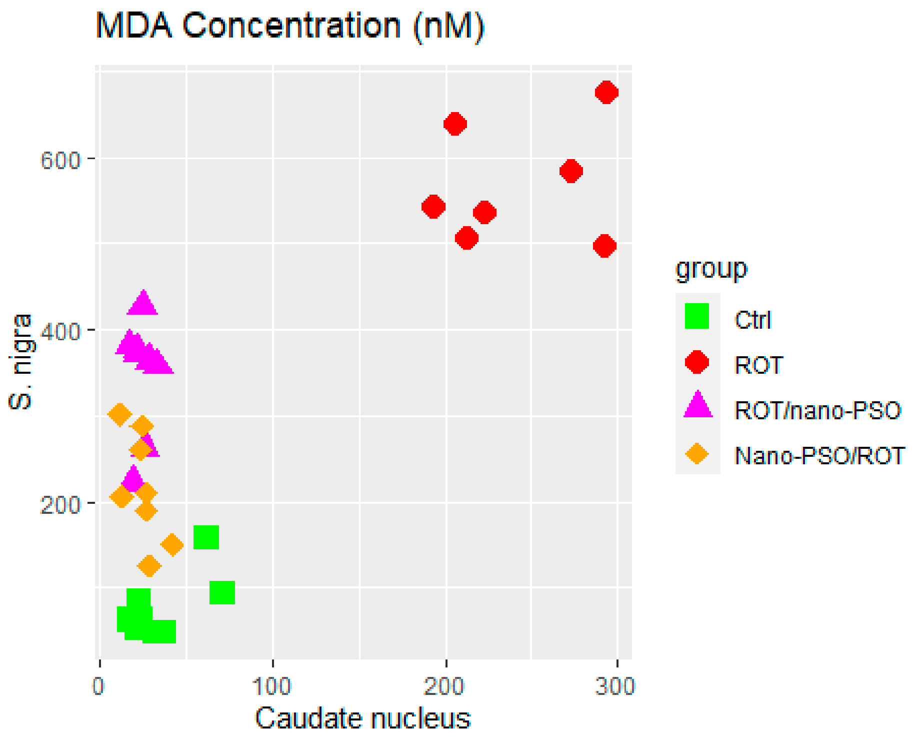

The graph corresponding to the PCA is:

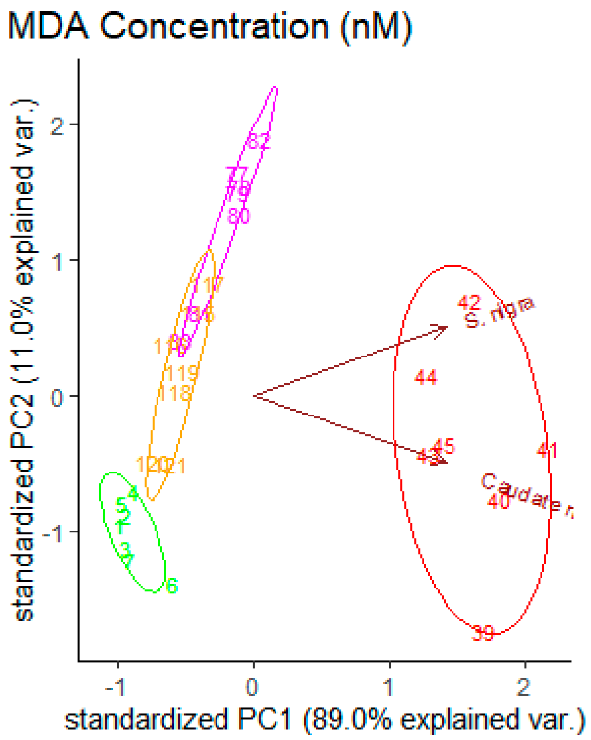

Ellipses Intersection Table (Percentages of Areas)

```
##                  Ctrl ROT ROT/nano-PSO Nano-PSO/ROT
## Ctrl                1   0    0.0000000   0.00000000
## ROT                 0   1    0.0000000   0.00000000
## ROT/nano-PSO        0   0    1.0000000   0.09596462
## Nano-PSO/ROT        0   0    0.1420486   1.00000000
## attr(,"class")
## [1] "Overlap"
```

Caudate nucleus		S. nigra		trat	id
21.79810		52.27000		Ctrl	1
23.17293		64.82000		Ctrl	2
31.44370		48.95780		Ctrl	3
22.37800		85.67000		Ctrl	4
17.43359	-	63.14279		Ctrl	5
71.02618	+	94.13623		Ctrl	6
37.09892		47.86867	-	Ctrl	7
61.89367		158.46170	+	Ctrl	8
293.16500		495.72500	-	ROT	39
273.18500		582.20000		ROT	40
294.40830	+	675.00400	+	ROT	41
205.68680		637.11900		ROT	42
213.49420		506.59490		ROT	43
193.81370	-	541.84060		ROT	44
223.11380		534.22070		ROT	45
NA		NA		ROT	46
16.93086	-	380.52000		ROT/nano-PSO	77
21.30021		376.79700		ROT/nano-PSO	78
21.45173		371.58500		ROT/nano-PSO	79
28.29117		364.13900		ROT/nano-PSO	80
25.88971		263.43970		ROT/nano-PSO	81
24.19259		427.53050	+	ROT/nano-PSO	82
18.53424		222.22920	-	ROT/nano-PSO	83
33.23757	+	358.22370		ROT/nano-PSO	84
23.68540		260.35500		Nano-PSO/ROT	115
12.65700		207.14230		Nano-PSO/ROT	116
24.30500		288.75160		Nano-PSO/ROT	117
27.21500		190.48900		Nano-PSO/ROT	118
27.03127		210.08200		Nano-PSO/ROT	119
28.70937		126.28840	-	Nano-PSO/ROT	120
41.70036	+	150.29170		Nano-PSO/ROT	121
11.52495	-	300.96280	+	Nano-PSO/ROT	122

File 1,Table 4: Antioxidant enzymes in blood

The Table 4, ANTIOXIDANT ENZYMES IN BLOOD, has three variables:

- Catalase specific activity (μU/ml): CAT_act
- (GPx activity product)NADPH (nmol/ml): GPx_act
- SOD Activity (% inhibition rate): SOD_act

The PCA graph indicates a low overlap between the pairs of groups: Ctrl vs ROT and between the pair ROT vs ROT/nano-PSO.

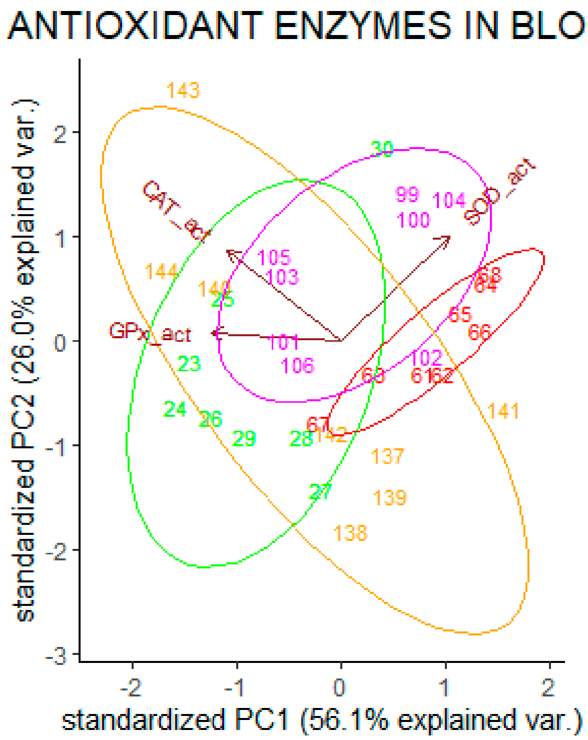

Ellipses Intersection Table (Percentages of Areas)

```
##                     Ctrl         ROT ROT/nano-PSO Nano-PSO/ROT
## Ctrl         1.0000000000 0.004419404    0.4227067   0.37997337
## ROT          0.0008760157 1.000000000    0.0000000   0.05350161
## ROT/nano-PSO 0.2807535320 0.000000000    1.0000000   0.21137427
## Nano-PSO/ROT 0.6170287367 0.438299352    0.5167949   1.00000000
## attr(,"class")
## [1] "Overlap"
```

Catalase		NADPH		SOD		trat	id
48.45399		127.846		2.40		Ctrl	23
34.53988		175.720	+	3.90		Ctrl	24
55.32515	+	100.165		5.40		Ctrl	25
33.33742		151.523		3.90		Ctrl	26
24.06135	-	83.073		2.60		Ctrl	27
34.53988		74.678	-	2.70		Ctrl	28
33.96319		117.942		2.40		Ctrl	29
44.62577		77.805		23.70	+	Ctrl	30
20.38037		62.606		14.70		ROT	61
23.95092		34.540		13.50		ROT	62
25.32515		79.067		12.30		ROT	63
20.09816	-	49.931		22.95	+	ROT	64
27.55828	+	28.944	-	17.40		ROT	65
20.85890		35.528		18.75		ROT	66
22.06135		114.019	+	8.80	-	ROT	67
23.20245		38.903		22.65		ROT	68
35.91411		76.653		23.55		ROT/nano-PSO	99
31.44785		81.427		23.10		ROT/nano-PSO	100
44.84663		81.591		6.50		ROT/nano-PSO	101
27.49693		39.479	-	13.40		ROT/nano-PSO	102
37.28834		134.760	+	14.80		ROT/nano-PSO	103
25.43558	-	85.706		27.45	+	ROT/nano-PSO	104
53.43558	+	84.554		10.10		ROT/nano-PSO	105
46.22086		57.503		4.10	-	ROT/nano-PSO	106
31.44785		17.915	-	3.30	-	Nano-PSO/ROT	137
13.75460		86.859		3.40		Nano-PSO/ROT	138
17.36196		56.900		5.40		Nano-PSO/ROT	139
53.95092		114.239		6.60		Nano-PSO/ROT	140
12.03681	-	24.829		15.75	+	Nano-PSO/ROT	141
17.01840		122.881	+	10.10		Nano-PSO/ROT	142
76.96933	+	117.695		14.00		Nano-PSO/ROT	143
63.76687		120.658		3.80		Nano-PSO/ROT	144

File 1, Table 5: Latency (sec)

The Latency (sec) table has two variables "21 days" and "42 days". The scatter plot of the data is provided below. From the graph we deduce that the ROT group is totally differentiated.

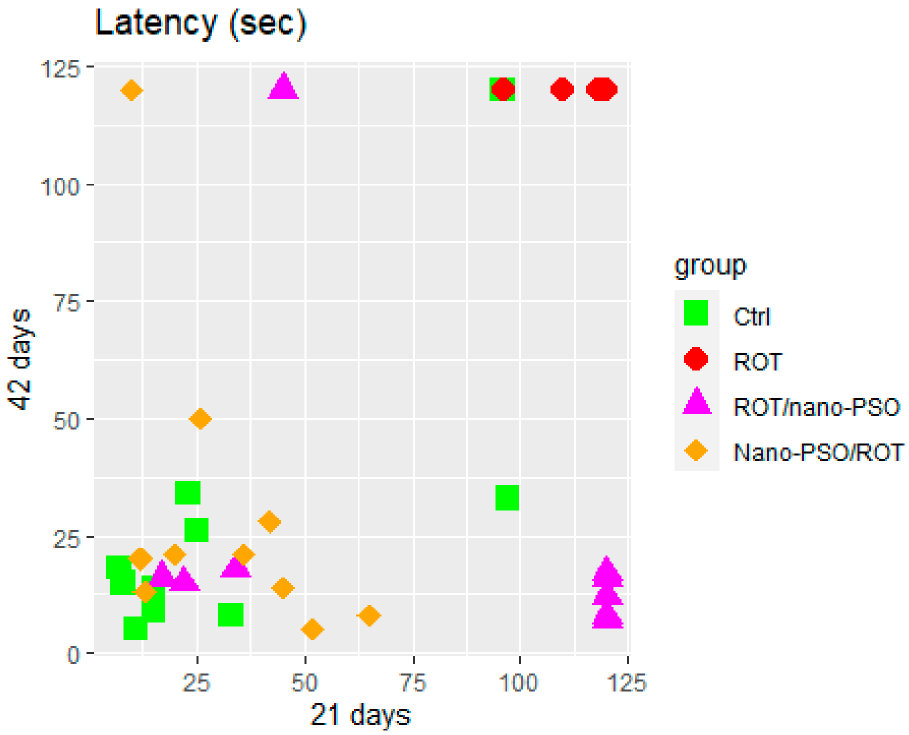

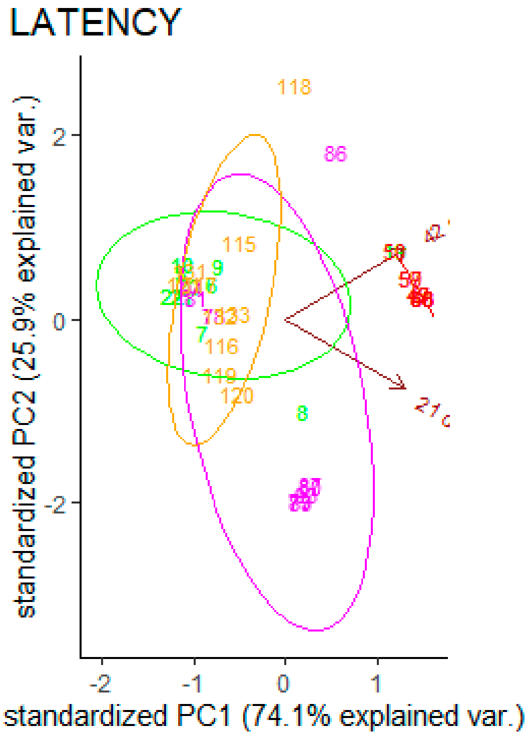

Ellipses Intersection Table (Percentages of Areas)

```
##                     Ctrl ROT ROT/nano-PSO Nano-PSO/ROT
## Ctrl         1.0000000   0    0.1411546    0.6032290
## ROT          0.0000000   1    0.0000000    0.0000000
## ROT/nano-PSO 0.2728444   0    1.0000000    0.3128462
## Nano-PSO/ROT 0.4251700   0    0.1140753    1.0000000
## attr(,"class")
## [1] "Overlap"
```

21 days		42 days		trat	id
8		15		Ctrl	5
25		26		Ctrl	6
33		8		Ctrl	7
97	+	33		Ctrl	8
23		34		Ctrl	9
7	-	18		Ctrl	10
96		120	+	Ctrl	19
15		14		Ctrl	20
15		9		Ctrl	21
11		5	-	Ctrl	22
118		120		ROT	47
120		120		ROT	48
118		120		ROT	49
96	-	120		ROT	50
120		120		ROT	55
120		120		ROT	56
110		120		ROT	57
120		120		ROT	58
110		120		ROT	59
120		120		ROT	60
120		7		ROT/nano-PSO	77
34		18		ROT/nano-PSO	78
120		8		ROT/nano-PSO	79
120		16		ROT/nano-PSO	80
22		15		ROT/nano-PSO	81
120		7		ROT/nano-PSO	85
45		120	+	ROT/nano-PSO	86
120		17		ROT/nano-PSO	87
120		12		ROT/nano-PSO	93
17	-	16		ROT/nano-PSO	94
26		50		Nano-PSO/ROT	115
45		14		Nano-PSO/ROT	116
20		21		Nano-PSO/ROT	117
10	-	120	+	Nano-PSO/ROT	118
52		5	-	Nano-PSO/ROT	119
65	+	8		Nano-PSO/ROT	120

21 days	42 days	trat	id
13	13	Nano-PSO/ROT	121
12	20	Nano-PSO/ROT	131
36	21	Nano-PSO/ROT	132
42	28	Nano-PSO/ROT	133

File 1, Table 6: Biochemical indicators

In the following table the application of PCA, the overlap of the groups indicates that they do not explain the variance of the observations.

The Table BIOCHEMICAL INDICATORS has three variables:

- GLUCOSE (mmol/L): GLUCOSE
- TRIGLYCERIDES (mmol/L): TRIGLYCERIDES
- CHOLESTEROL (mmol/L): CHOLESTEROL

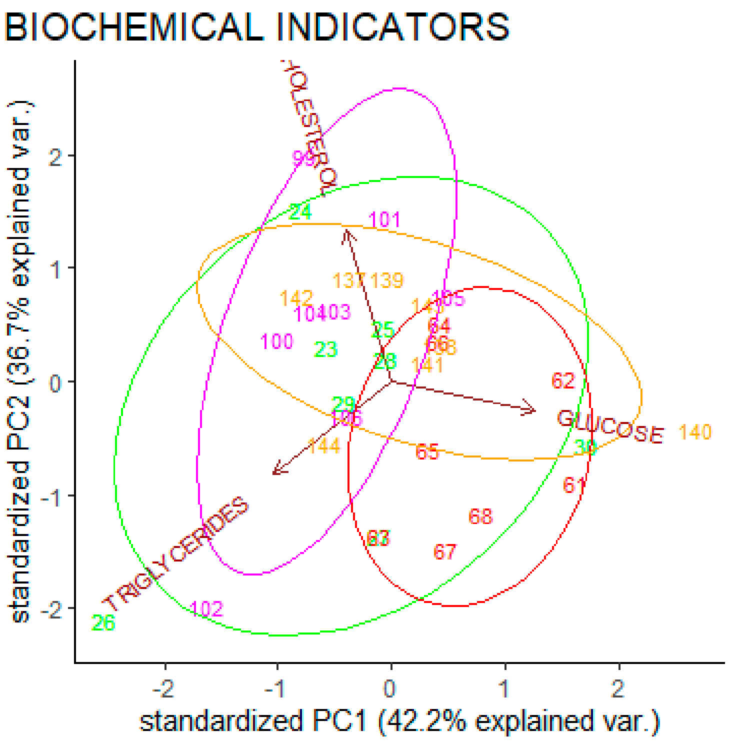

Ellipses Intersection Table (Percentages of Areas)

```
##                    Ctrl       ROT  ROT/nano-PSO Nano-PSO/ROT
## Ctrl          1.0000000 0.8245595    0.85226617    0.9360460
## ROT           0.3045102 1.0000000    0.09309552    0.3047027
## ROT/nano-PSO  0.4228680 0.1250773    1.00000000    0.4855064
## Nano-PSO/ROT  0.4139820 0.3649058    0.43276254    1.0000000
```

```
## attr(,"class")
## [1] "Overlap"
```

GLUCOSE		TRIGLYCERIDES		CHOLESTEROL		trat	id
82		149		159		Ctrl	23
81		142		165	+	Ctrl	24
83		123		158		Ctrl	25
76	-	272	+	155		Ctrl	26
83		150		150	-	Ctrl	27
85		129		157		Ctrl	28
81		146		156		Ctrl	29
113	+	101	-	152		Ctrl	30
129	+	140		154		ROT	61
111		99	-	155		ROT	62
83	-	150	+	150		ROT	63
93		115		158	+	ROT	64
85		120		152		ROT	65
91		114		157		ROT	66
98		149		150		ROT	67
108		147		152		ROT	68
76		125		166	+	ROT/nano-PSO	99
82		171		161		ROT/nano-PSO	100
82		107	-	162		ROT/nano-PSO	101
71	-	215	+	151	-	ROT/nano-PSO	102
81		139		160		ROT/nano-PSO	103
77		143		160		ROT/nano-PSO	104
94	+	110		159		ROT/nano-PSO	105
84		152		156		ROT/nano-PSO	106
93		151		163		Nano-PSO/ROT	137
87		106		156		Nano-PSO/ROT	138
100		147		163		Nano-PSO/ROT	139
156	+	129		157		Nano-PSO/ROT	140
78		97	-	154	-	Nano-PSO/ROT	141
77	-	147		161		Nano-PSO/ROT	142
92		116		159		Nano-PSO/ROT	143
96		188	+	158		Nano-PSO/ROT	144

Archivo 2,Tabla 1: Neurotransmiters and Metabolites in Caudate nucleus

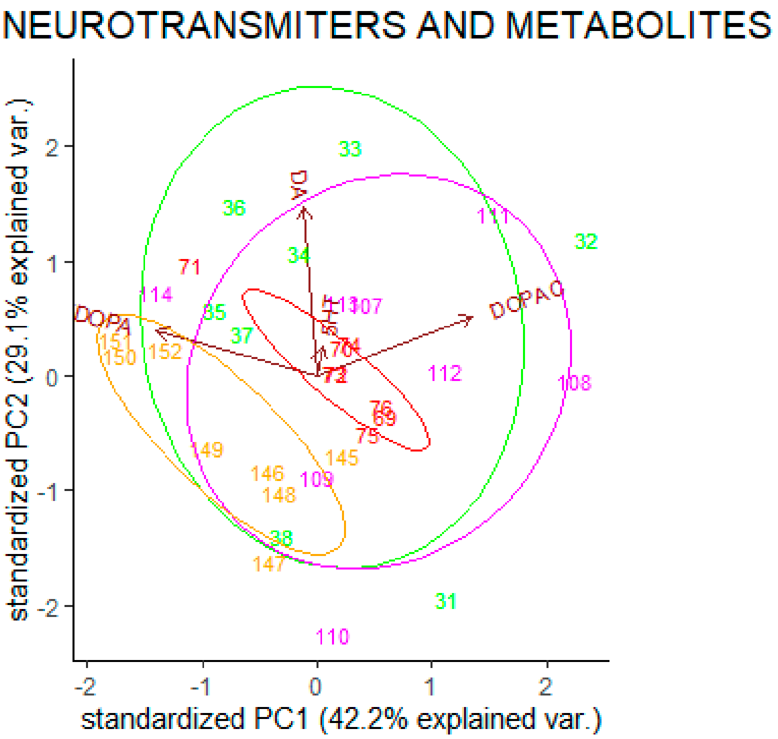

Ellipses Intersection Table (Percentages of Areas)

```
##                      Ctrl ROT ROT/nano-PSO Nano-PSO/ROT
## Ctrl         1.00000000   1   0.85711174     0.940680
## ROT          0.07137605   1   0.08724572     0.000000
## ROT/nano-PSO 0.70120629   1   1.00000000     0.881247
## Nano-PSO/ROT 0.18287709   0   0.20941446     1.000000
## attr(,"class")
## [1] "Overlap"
```

DA		DOPAC		DA/DOPA		5HT		trat	id
10.477	-	30.451		0.3440610	-	0.799	-	Ctrl	31
31.587		69.761	+	0.4527888		1.525		Ctrl	32
43.592	+	32.364		1.3469287		1.377		Ctrl	33
36.231		23.154		1.5647836		1.259		Ctrl	34
31.063		14.312		2.1704164	+	1.716		Ctrl	35
38.186		18.278		2.0891782		2.457	+	Ctrl	36
30.193		15.697		1.9234886		1.096		Ctrl	37
16.137		11.929	-	1.3527538		0.894		Ctrl	38
23.560		27.620		0.8530051	-	1.696		ROT	69
29.462		25.090		1.1742527		1.012	-	ROT	70
34.500	+	14.250	-	2.4210526	+	1.290		ROT	71
20.840	-	18.500		1.1264865		8.490	+	ROT	72
27.270		22.900		1.1908297		1.420		ROT	73
29.350		25.820		1.1367157		1.345		ROT	74
22.820		24.736		0.9225420		1.350		ROT	75
24.470		27.760	+	0.8814841		1.400		ROT	76
32.010		29.320		1.0917462		1.417		ROT/nano-PSO	107
21.647		63.160	+	0.3427327	-	1.443		ROT/nano-PSO	108
20.141		17.210		1.1703080		1.245		ROT/nano-PSO	109
9.521	-	11.840		0.8041385		1.047	-	ROT/nano-PSO	110
35.973	+	52.610		0.6837673		1.582		ROT/nano-PSO	111
25.489		38.400		0.6637760		1.923	+	ROT/nano-PSO	112
32.435		26.420		1.2276684		1.324		ROT/nano-PSO	113
31.400		11.440	-	2.7447552	+	1.670		ROT/nano-PSO	114
21.860		20.950	+	1.0434368	-	0.930		Nano-PSO/ROT	145
20.420		13.080		1.5611621		1.024		Nano-PSO/ROT	146
14.410	-	10.170		1.4169125		0.620	-	Nano-PSO/ROT	147
18.940		13.210		1.4337623		1.130		Nano-PSO/ROT	148
21.050		9.880		2.1305668		1.355		Nano-PSO/ROT	149
26.420		8.640	-	3.0578704		1.185		Nano-PSO/ROT	150
27.270	+	8.770		3.1094641	+	1.445		Nano-PSO/ROT	151
27.260		10.470		2.6036294		1.776	+	Nano-PSO/ROT	152

File 2, Table 2: Neurotransmitters and Metabolites in S. nigra

In this single case, the names of the individuals indicate how the pools were formed to obtain the variable.

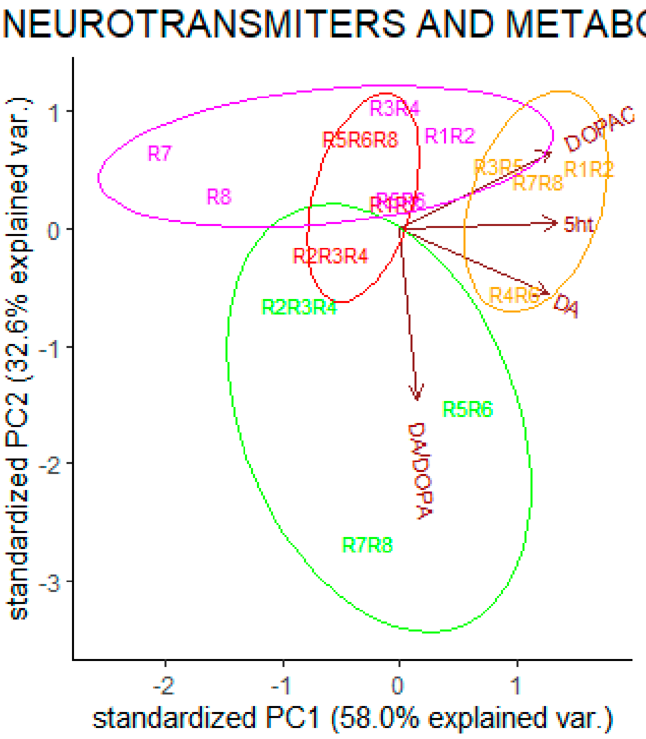

Ellipses Intersection Table (Percentages of Areas)

```
##                   Ctrl       ROT  ROT/nano-PSO  Nano-PSO/ROT
## Ctrl         1.00000000 0.4898054   0.05578912     0.0000000
## ROT          0.08459539 1.0000000   0.21007202     0.0000000
## ROT/nano-PSO 0.02785990 0.6074005   1.00000000     0.5440581
## Nano-PSO/ROT 0.00000000 0.0000000   0.26462519     1.0000000
## attr(,"class")
## [1] "Overlap"
```

DA		DOPAC		DA/DOPA		5ht		trat	rat
1.161	-	17.670		0.06570458	-	1.080	-	Ctrl	R2R3R4
2.760	+	29.870	+	0.09240040		1.850		Ctrl	R5R6
1.420		9.140	-	0.15536105	+	1.990	+	Ctrl	R7R8
1.137	+	30.827	+	0.03688325		2.131		ROT	R1R7
1.063		21.336	-	0.04982190	+	1.557	-	ROT	R2R3R4
0.560	-	29.851		0.01875984	-	2.169	+	ROT	R5R6R8
1.170	+	49.440	+	0.02366505		2.322		ROT/nano-PSO	R1R2
0.750		46.420		0.01615683		1.960		ROT/nano-PSO	R3R4
1.030		28.770		0.03580118	+	2.390	+	ROT/nano-PSO	R5R6
0.070	-	7.150	-	0.00979021	-	0.356	-	ROT/nano-PSO	R7
0.340		12.080		0.02814570		0.803		ROT/nano-PSO	R8
2.360		68.850	+	0.03427741		2.806	+	Nano-PSO/ROT	R1R2
1.960	-	60.530		0.03238064	-	2.034	-	Nano-PSO/ROT	R3R5
2.450	+	40.300	-	0.06079404	+	2.523		Nano-PSO/ROT	R4R6
2.110		59.030		0.03574454		2.557		Nano-PSO/ROT	R7R8

File 3,Table 1: Relative Gene Expression in Caudate nucleus

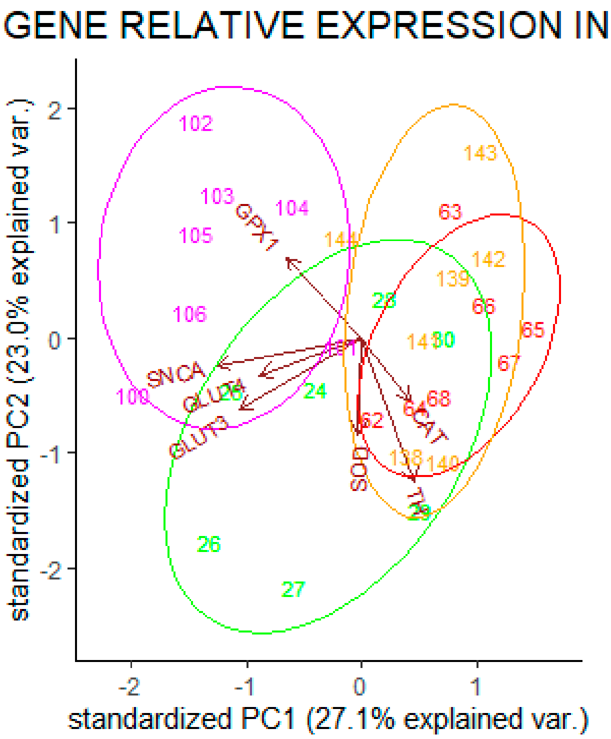

Ellipses Intersection Table (Percentages of Areas)

```
##                    Ctrl       ROT  ROT/nano-PSO  Nano-PSO/ROT
## Ctrl          1.0000000 0.5597952     0.2132752     0.4753830
## ROT           0.2291537 1.0000000     0.0000000     0.5375551
## ROT/nano-PSO  0.1563337 0.0000000     1.0000000     0.0000000
## Nano-PSO/ROT  0.2963056 0.8185055     0.0000000     1.0000000
## attr(,"class")
## [1] "Overlap"
```

CAT		GPX1		SOD		SNCA		TH		GLUT3		GLUT4		trat	id
0.4204		0.4463	+	0.8710		0.4070		0.5170		0.5054		0.4138		Ctrl	24
0.6457		0.2462		0.0454	-	0.8867		0.6250		0.8232		0.4513		Ctrl	25
0.8714		0.0096		0.0833		0.9801	+	0.8696		0.9361	+	0.7984	+	Ctrl	26
0.9450	+	0.0077		0.5290		0.8951		0.9039	+	0.8609		0.3650		Ctrl	27
0.4583		0.0049	-	0.0537		0.5697		0.4913	-	0.4875	-	0.0116	-	Ctrl	28
0.0432		0.0271		0.9427	+	0.0000	-	0.8244		0.8345		0.0777		Ctrl	29
0.0388	-	0.0948		0.1176		0.0001		0.7352		0.6326		0.0431		Ctrl	30
0.4990		0.3245		0.2666		0.6829	+	0.9005	+	0.3676	+	0.2016		ROT	62
0.3647		0.3539	+	0.1470		0.2023		0.4534	-	0.1563		0.0090	-	ROT	63
0.9006	+	0.0012		0.0240		0.2087		0.7540		0.1840		0.8912	+	ROT	64
0.7486		0.0017		0.0128		0.1468		0.7148		0.1289		0.0162		ROT	65
0.0386		0.0002	-	0.0002	-	0.1219		0.7737		0.0819	-	0.2836		ROT	66
0.0448		0.0002		0.3544	+	0.1081	-	0.8162		0.1290		0.0768		ROT	67
0.0373	-	0.0344		0.2049		0.2089		0.8987		0.3228		0.3215		ROT	68
0.0197		0.7500		0.7090	+	0.9268	+	0.6075		0.8697	+	0.5381		ROT/nano-PSO	100
0.0069	-	0.2951	-	0.5012		0.3467		0.6398	+	0.2153	-	0.6791		ROT/nano-PSO	101
0.0163		0.8496	+	0.0115		0.8151		0.2276	-	0.6265		0.1321		ROT/nano-PSO	102
0.0227		0.7788		0.0011	-	0.9150		0.4561		0.5746		0.1307	-	ROT/nano-PSO	103
0.0239	+	0.7268		0.0578		0.1737	-	0.4771		0.5594		0.4814		ROT/nano-PSO	104
0.0224		0.6720		0.0463		0.4689		0.4345		0.6274		0.8079	+	ROT/nano-PSO	105
0.0210		0.5621		0.0638		0.7307		0.6226		0.6815		0.6989		ROT/nano-PSO	106
0.9224		0.5944		0.6638	+	0.1983	+	0.8208		0.6850		0.0434		Nano-PSO/ROT	138
0.9478		0.8479	+	0.4690		0.1714		0.6063		0.0235	-	0.1489		Nano-PSO/ROT	139
0.9815	+	0.5662		0.4803		0.1110		0.9371	+	0.5860		0.0943		Nano-PSO/ROT	140
0.8642		0.5479		0.0464		0.1179		0.7406		0.6622		0.0002	-	Nano-PSO/ROT	141
0.9804		0.6098		0.0202		0.0383	-	0.6451		0.0704		0.1985		Nano-PSO/ROT	142
0.3597	-	0.2367	-	0.0172	-	0.1115		0.3222		0.0346		0.0078		Nano-PSO/ROT	143
0.7496		0.2956		0.0848		0.1509		0.2222	-	0.7649	+	0.2485	+	Nano-PSO/ROT	144

Correlation of Tables 4, 6 and 9

Tables 4 (ANTIOXIDANT ENZYMES IN BLOOD), 6 (BIOCHEMICAL INDICATORS) and 9 (GENE RELATIVE EXPRESSION IN Caudate nucleus) have information on the same individuals which allows us to look for correlations between the data. In this case the resulting table has 13 numerical variables. However, the explanation of variance seems reduced: PC1 explains 21.8%; PC1 and PC2 explain 37.39%; PC1 to PC3: explain 51.23%. In the PC1-PC2 space, the difference between the ROT and ROT/nano-PSO groups seems to widen.

```
##  [1]  21.82032  37.38717  51.22949  61.40356  70.59270  78.28129  85.3
7295
##  [8]  90.43292  93.85203  96.48686  98.23967  99.33729 100.00000
```

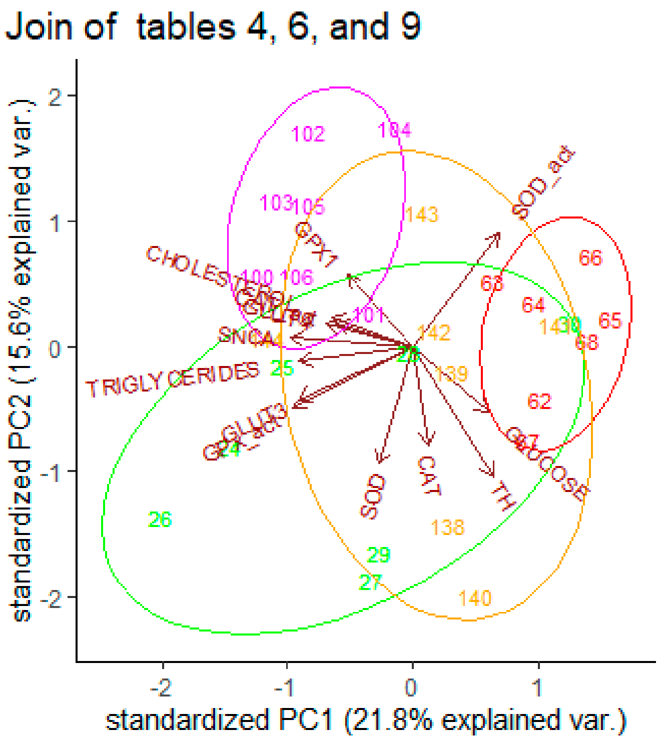

Ellipses Intersection Table (Percentages of Areas)

```
##                     Ctrl       ROT  ROT/nano-PSO  Nano-PSO/ROT
## Ctrl          1.00000000 0.6898638    0.05757722     0.6328744
## ROT           0.14595442 1.0000000    0.00000000     0.2099726
## ROT/nano-PSO  0.01596714 0.0000000    1.00000000     0.1323717
## Nano-PSO/ROT  0.55972809 0.8777448    0.42216124     1.0000000
## attr(,"class")
## [1] "Overlap"
```

Conclusion

The differences shown in the different groups according to the tables provided are illustrated in the following table. It indicates the table with the TX symbology, where X represents the internal enumeration followed by the description of it. A "+" sign in red signifies a strong separation between the groups by means of the information provided in the table. The appearance of a table without this sign means that the intersection between the groups is reduced.

	**Group**		
**Group**	**ROT**	**ROT/nano-PSO**	**Nano-PSO/ROT**
**Ctrl**	T1:TH+ neuron avg.+<br>T2:α-synuclein conc.+<br>T3:MDA conc.+<br>T4:Antiox. Enzymes in Blood<br>T5:Latency+	T1:TH+ neuron avg.+<br>T2:α-synuclein conc.+<br>T3:MDA conc.+<br>T8:NeuroT. & MetaB. in SN	T1:TH+ neuron avg.+<br>T2:α-synuclein conc.+<br>T3:MDA conc.+<br>T8:NeuroT. & MetaB in SN+
**ROT**		T1:TH+ neuron avg.<br>T2:α-synuclein conc.+<br>T3:MDA conc.+<br>T4:Antioxidant Enzymes in Blood<br>T5:Latency+<br>T9:Gene Rel. Expr. in CN+	T1:TH+ neuron avg.+<br>T2:α-synuclein conc.+<br>T3:MDA conc.+<br>T5:Latency+<br>T6:Biochemical indicators<br>T7:NeuroT. & MetaB. in CN+<br>T8:NeuroT. & MetaB. in SN+
**ROT/nano-PSO**			T1:TH+ neuron avg.+<br>T3:MDA conc.<br>T9:Gene Rel. Expr. in CN+

Dendogramas

A dendogram is a tree-shaped representation of the degree of similarity that exists between individuals in a population. The similarity or similarity between individuals is measured from a distance: two individuals are very similar if the distance between them is reduced. In a dendogram the distance between two individuals is measured as the distance (height if the dendogram is vertical) from their branches to the point of attachment of them. The separation or overlap shown in PCA in the previous graphs can be corroborated by means of these dendograms. The following groupings and their mixtures are consistent with those found by PCA.

File 1,Table 1: Mean of TH+ Neurons (10^3)

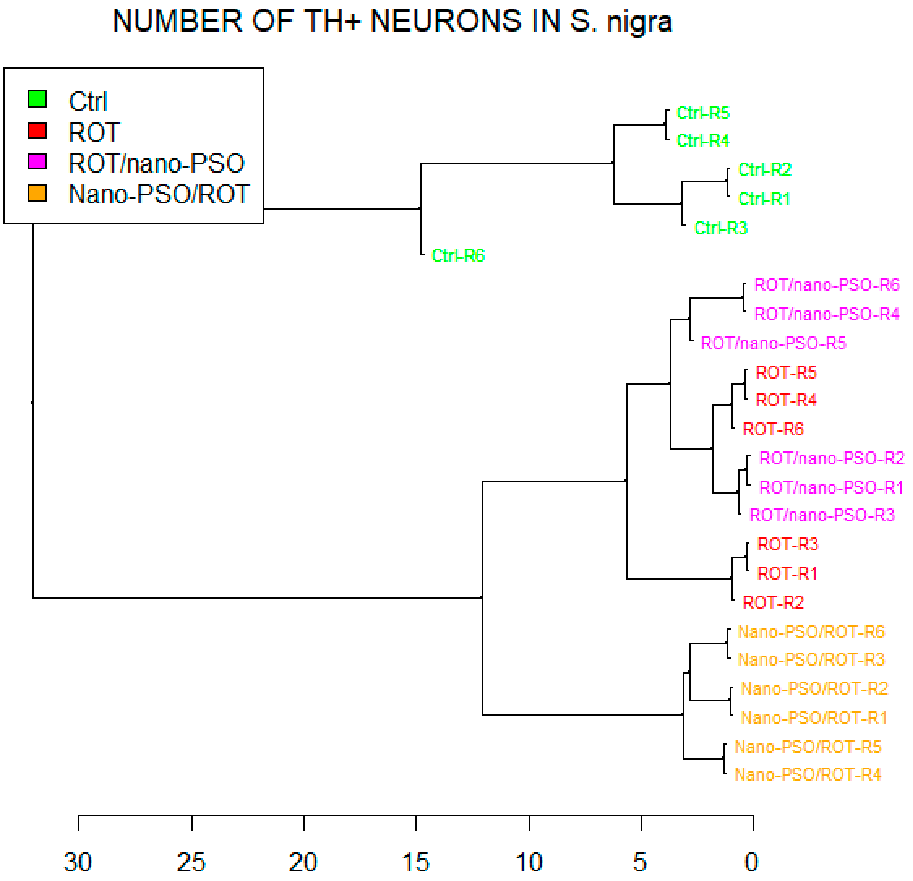

File 1,Tabl2 2: Concentration of α-synuclein (pg/ml)

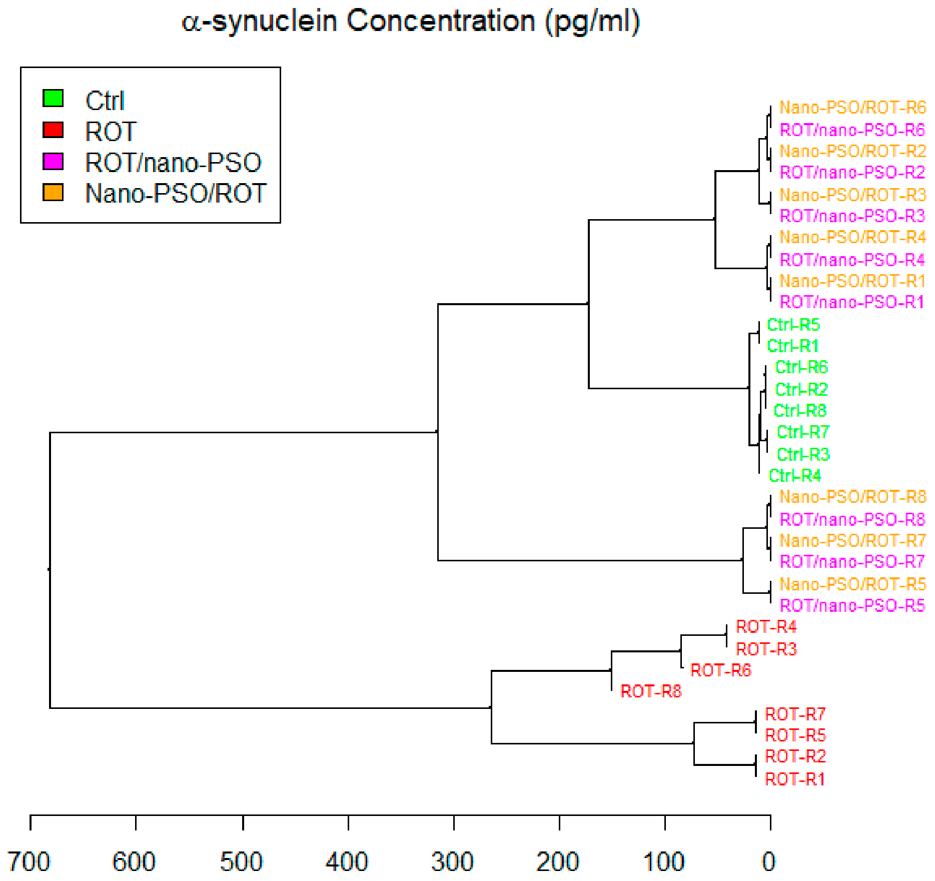

File 1,Table 3: MDA Concentration (nM)

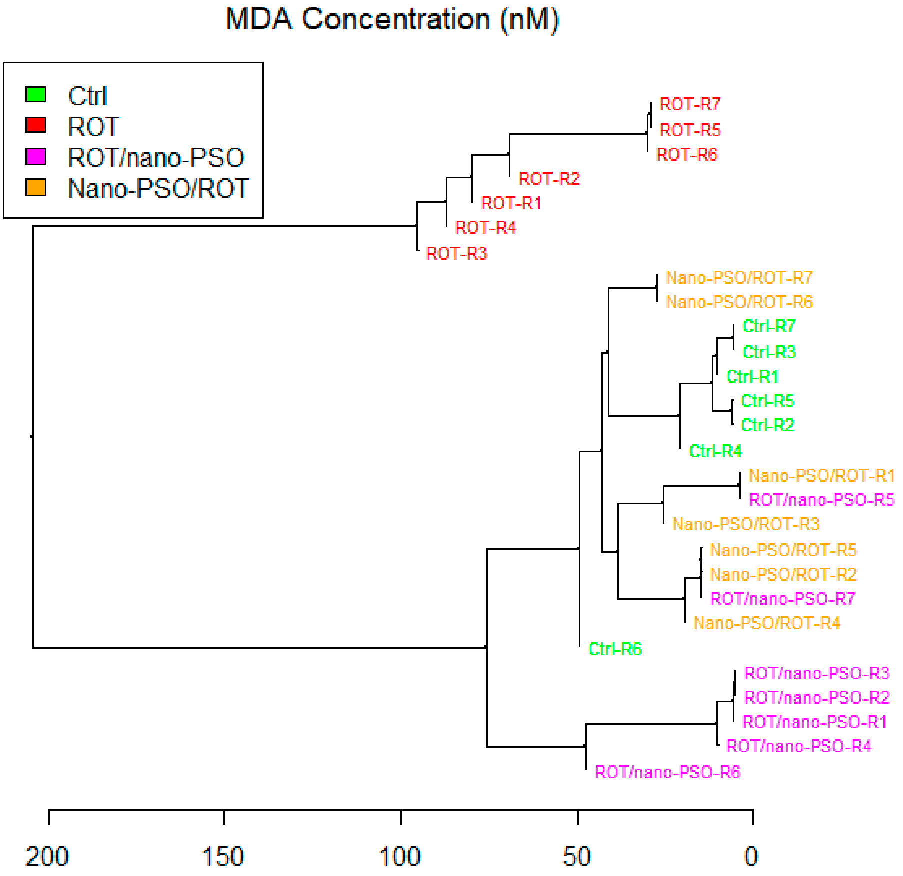

File 1,Table 4: Antioxidant enzymes in blood

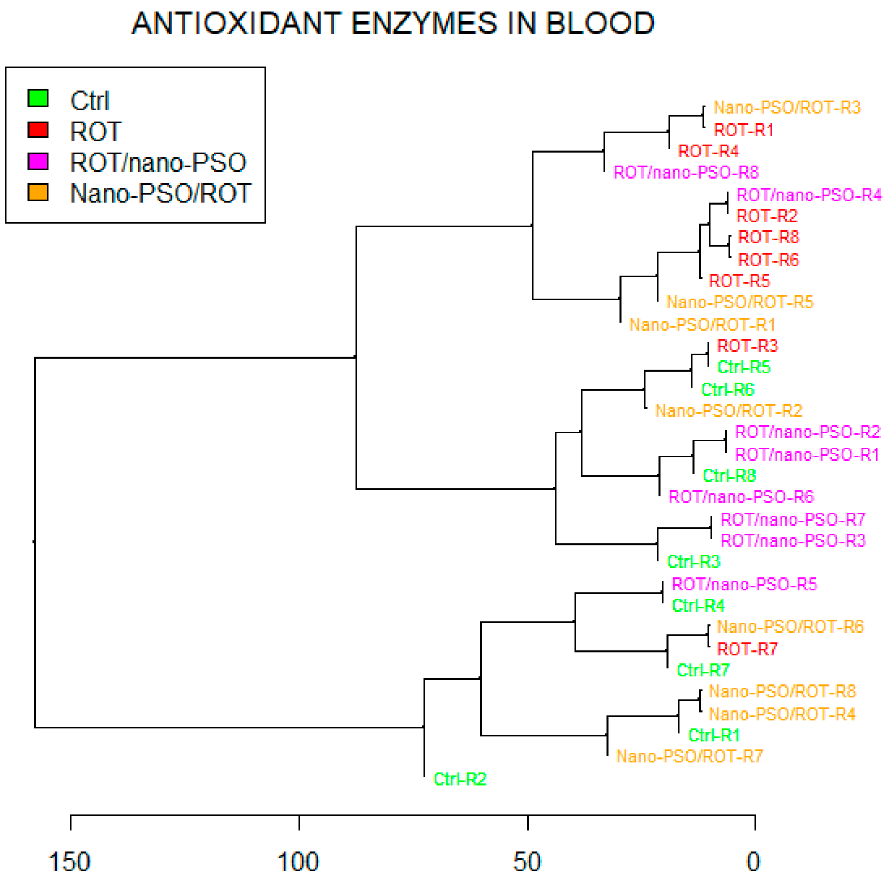

File 1,Tabea 5: Incline beam test (Latency in sec)

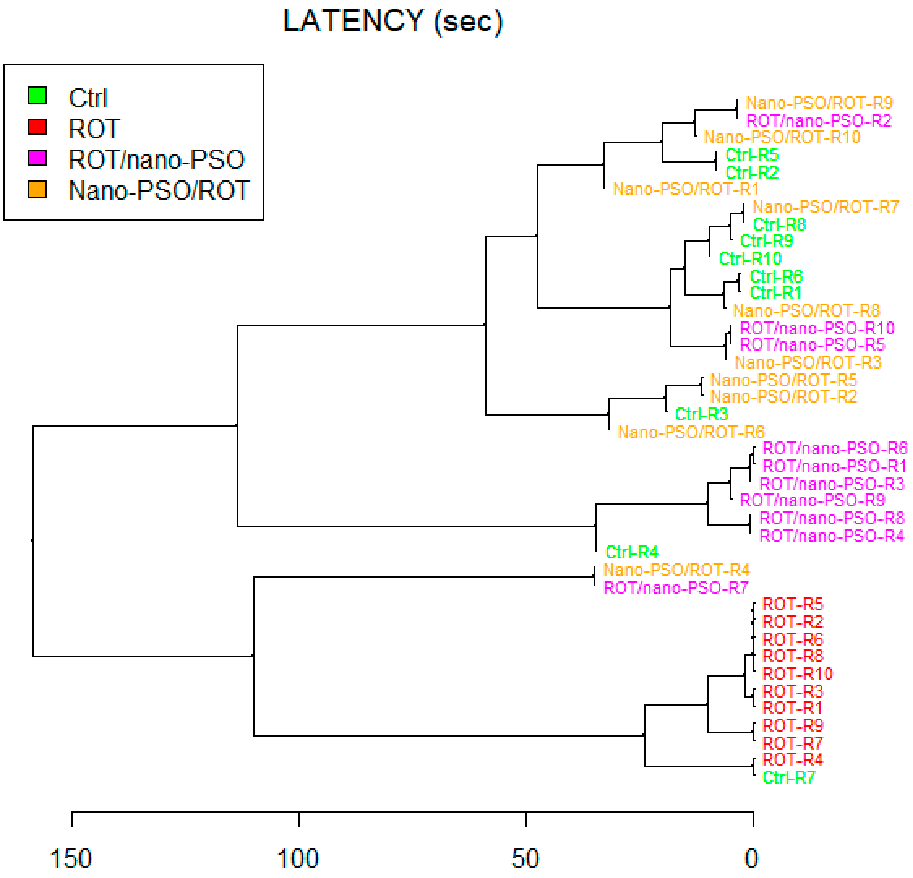

File 1,Table 6: Biochemical indicators

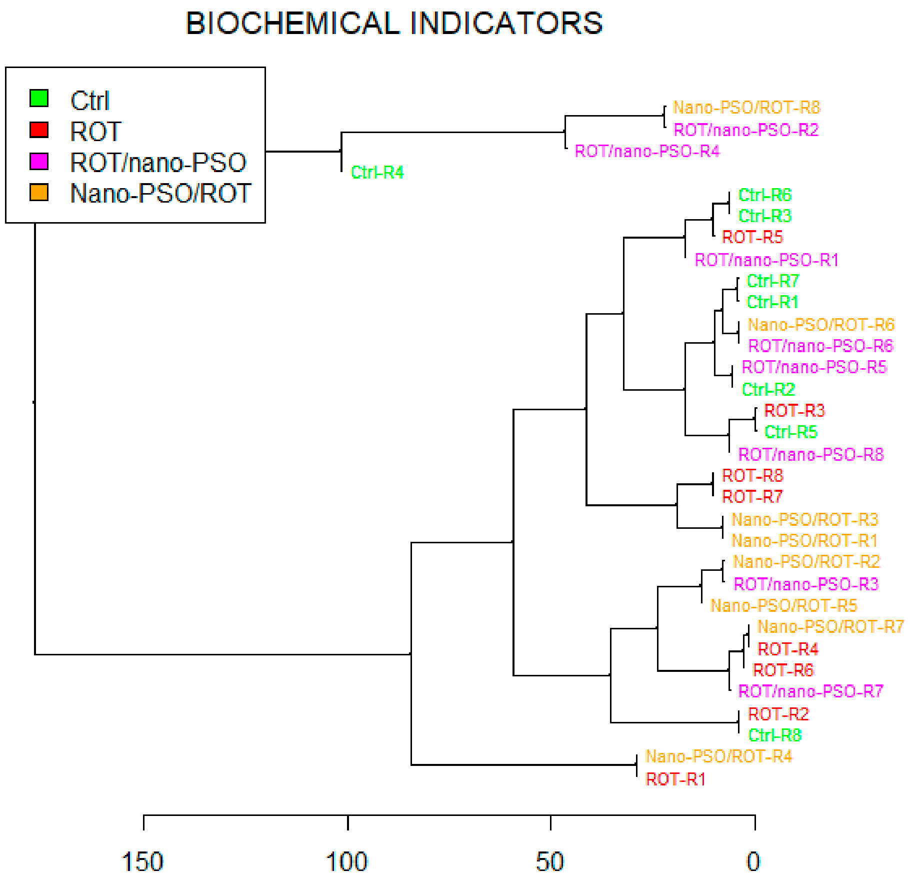

File 2,Tabl1 1: Neurotransmitters and Metabolites in Caudate nucleus

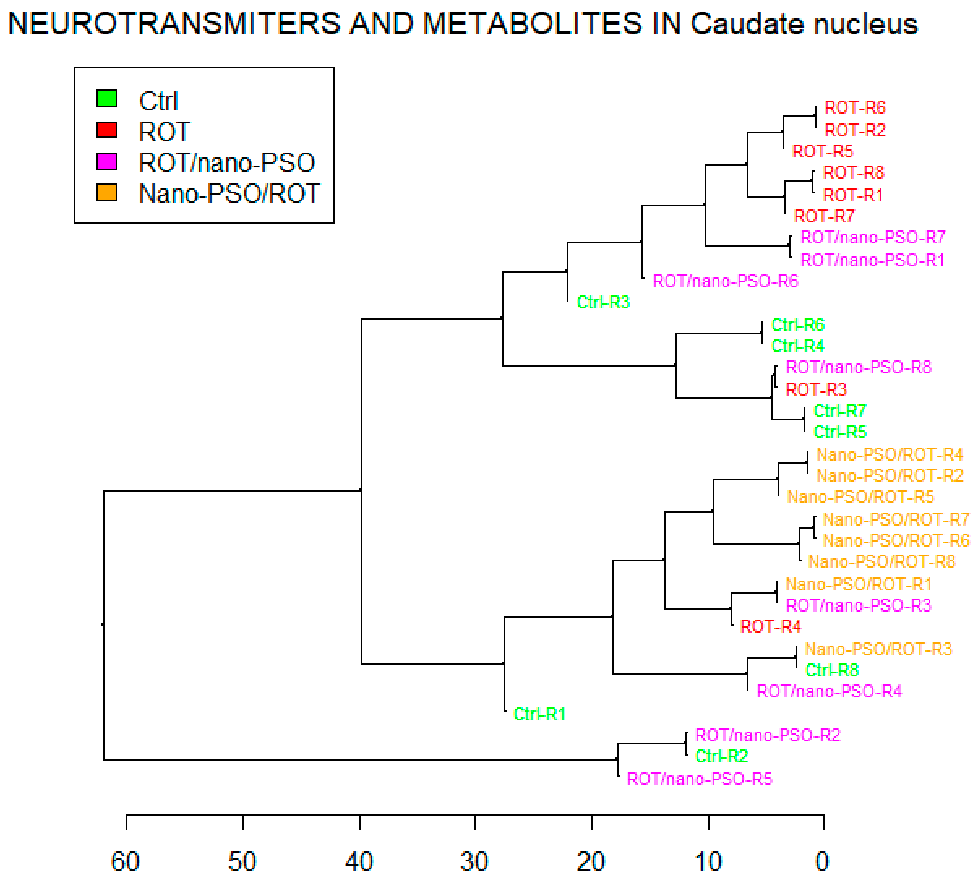

File 2,Table 2: Neurotransmitters and Metabolites in S. nigra

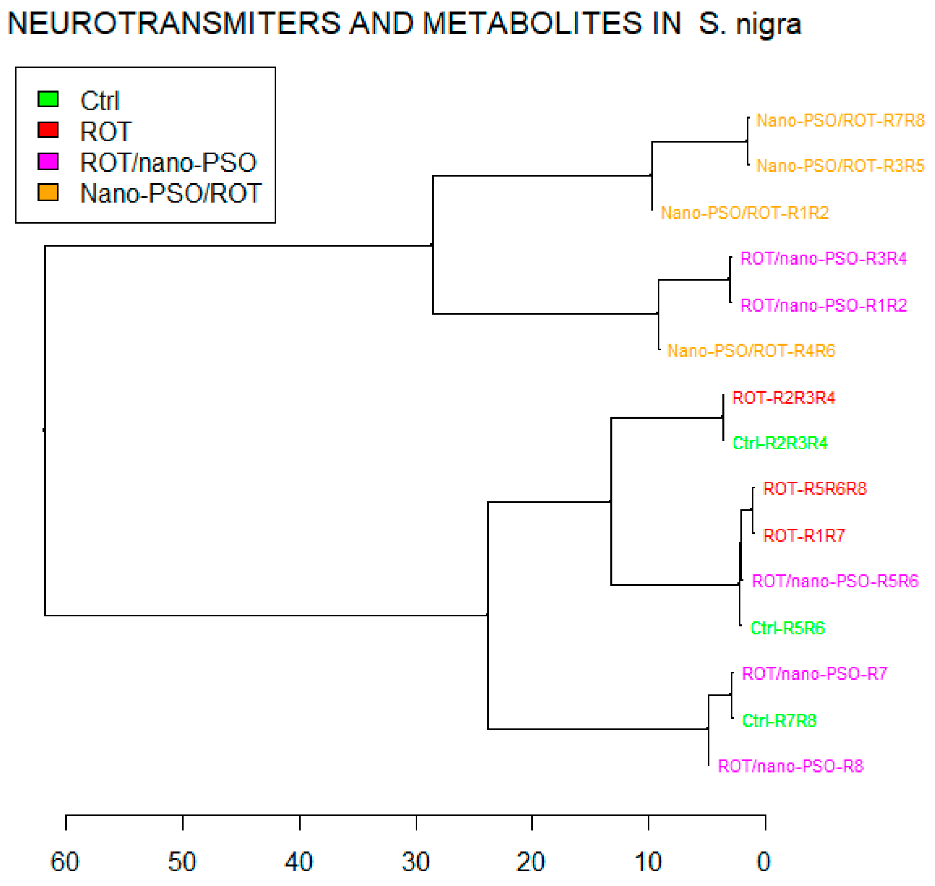

File 3,Table 1: Relative Gene Expression in Caudate nucleus

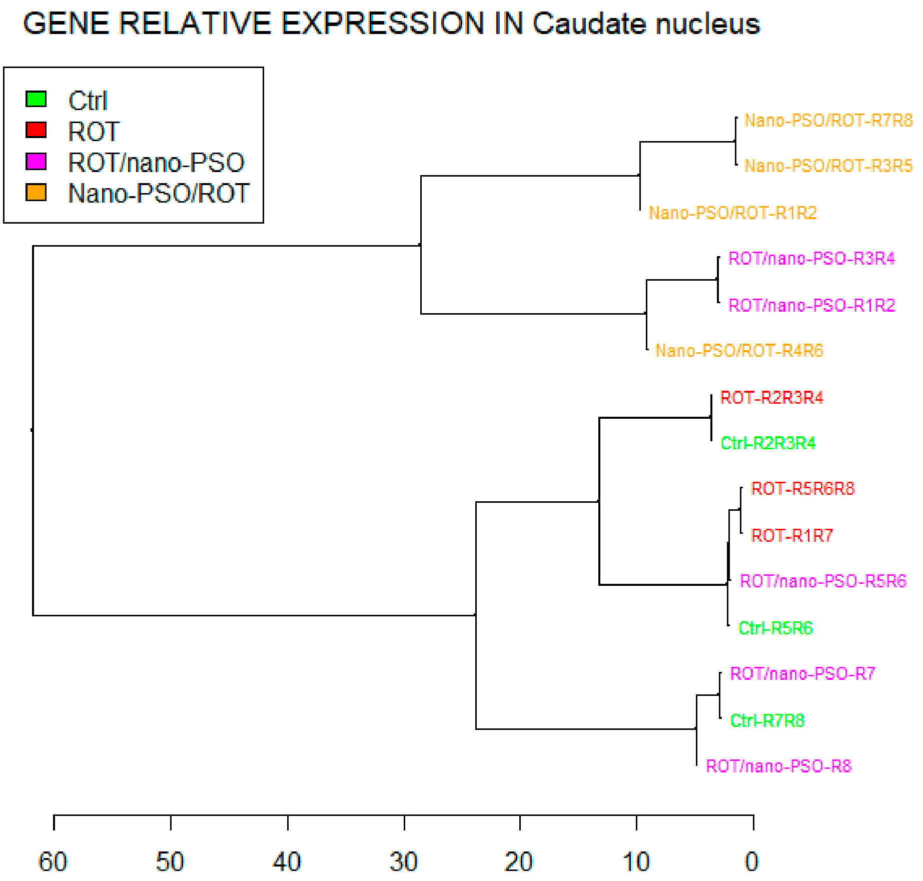

Annex 1

Treatment comparison

File 1,Table 1: Mean number of TH+ neurons in S. nigra

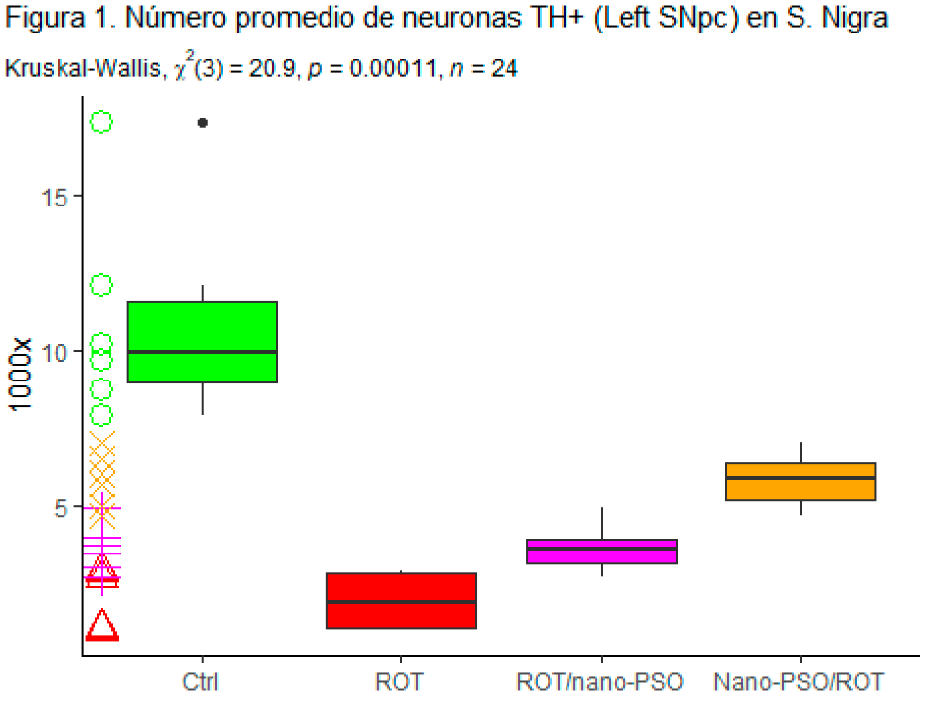

Figura 2. Número promedio de neuronas TH+ (Right SNpc) en S. Nigra

Kruskal-Wallis, $\chi^2(3) = 19.55$, $p = 0.00021$, $n = 24$

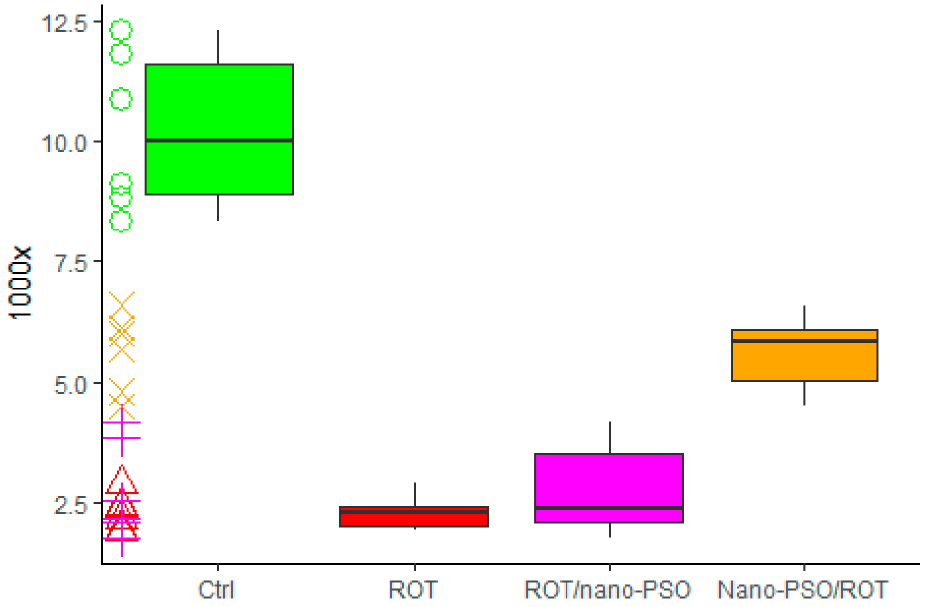

Figura 3. Número promedio de neuronas TH+ (Total) en S. Nigra

Kruskal-Wallis, $\chi^2(3) = 21.62$, $p = <0.0001$, $n = 24$

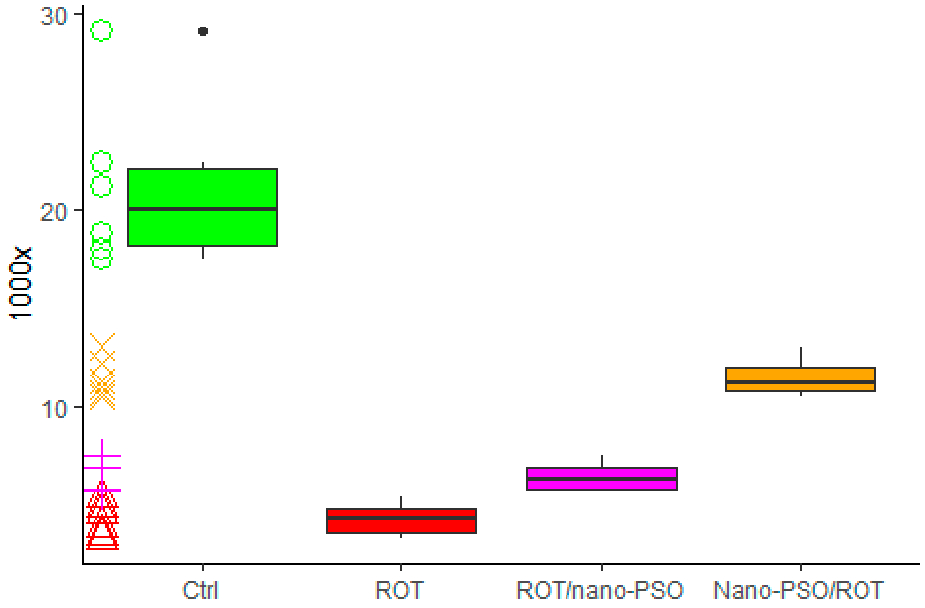

Archivo 1,Tabla 2:Concentración a-synuclein (pg/ml)

Figura 4. Concentración a-synuclein en Caudate nucleus

Kruskal-Wallis, $\chi^2(3) = 20.31$, $p = 0.00015$, $n = 32$

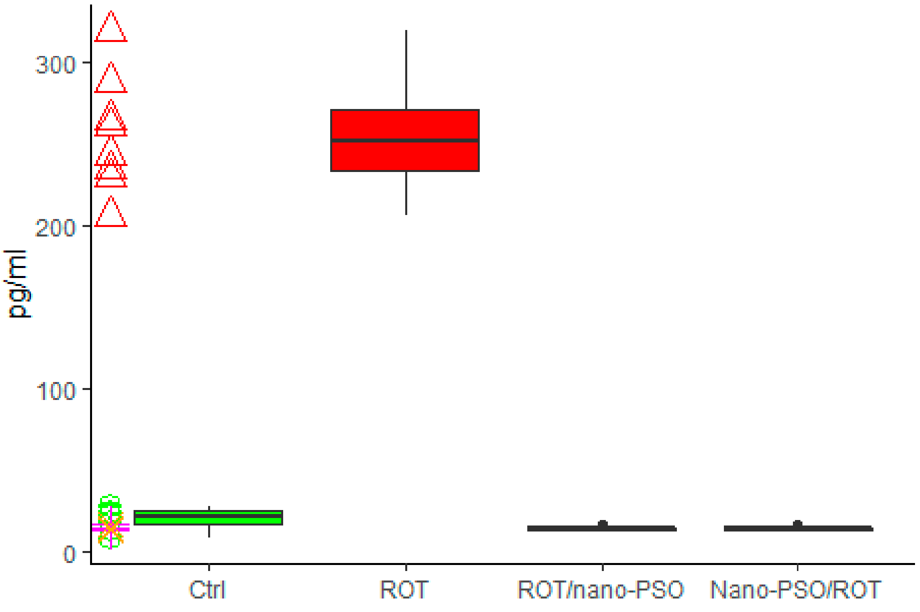

Figura 5. Concentración a-synuclein en S. Nigra

Kruskal-Wallis, $\chi^2(3) = 26.23$, $p = <0.0001$, $n = 32$

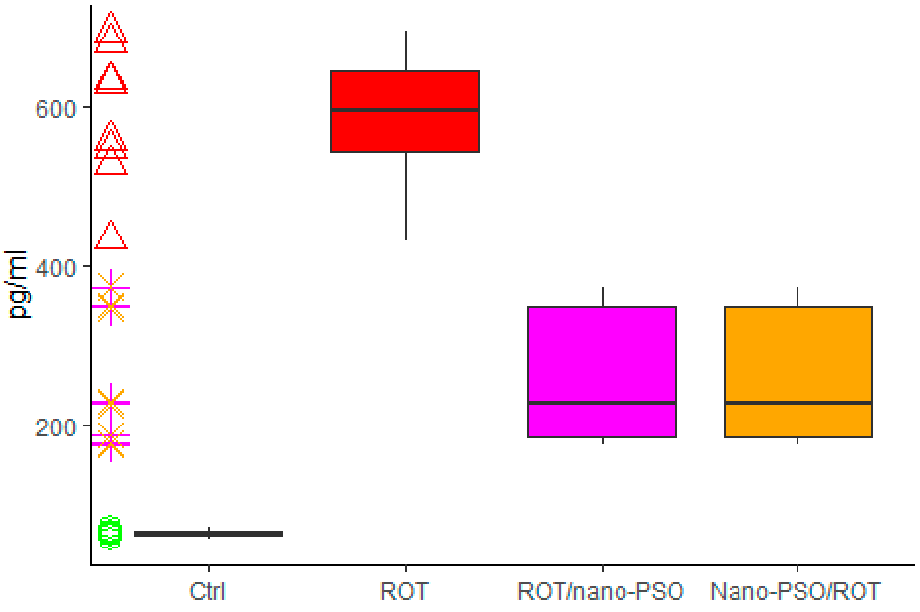

Archivo 1,Tabla 3:Concentración MDA (nM)

Figura 6. Concentración MDA en Caudate nucleus

Kruskal-Wallis, $\chi^2(3) = 16.63$, $p = 0.00084$, $n = 32$

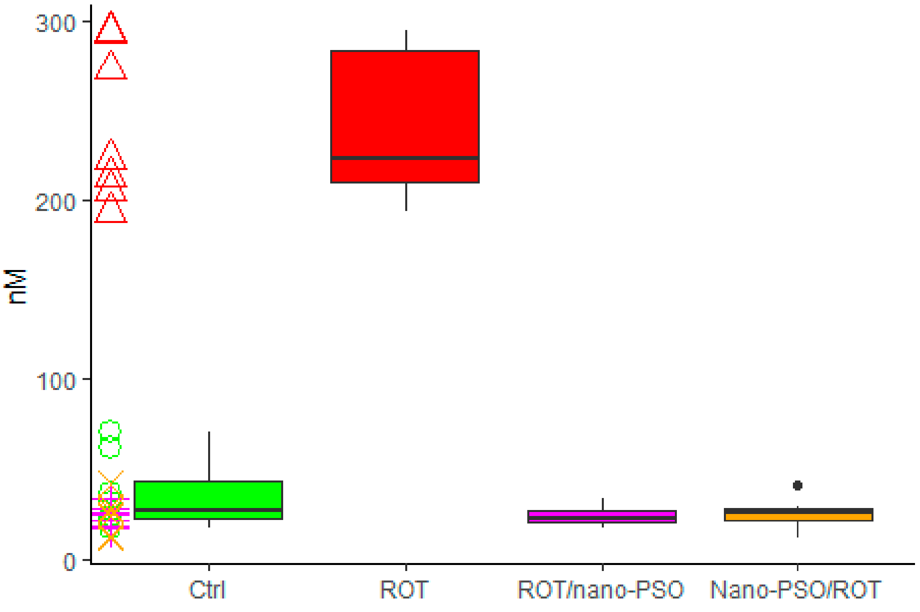

Figura 7. Concentracion MDA en S. nigra

Kruskal-Wallis, $\chi^2(3) = 26.84$, $p = <0.0001$, $n = 32$

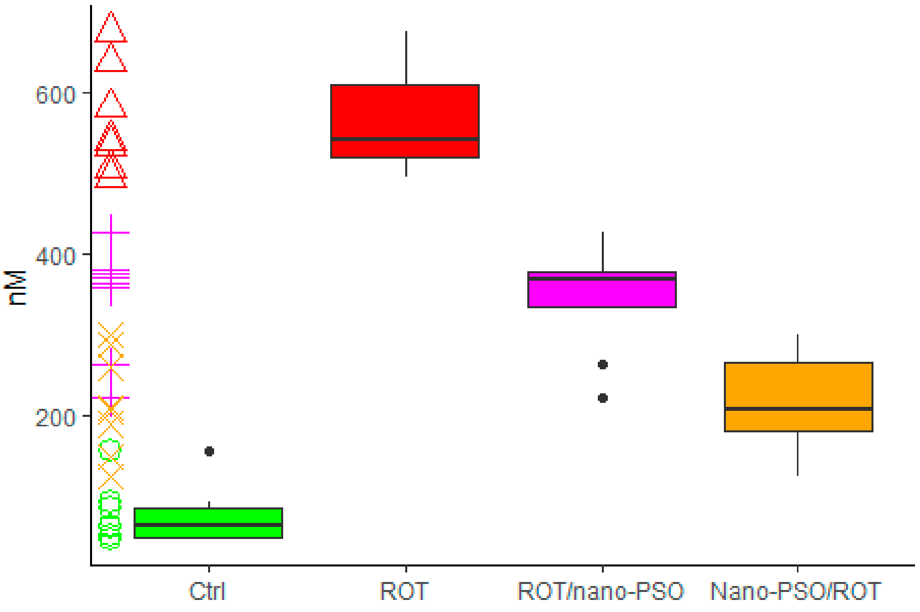

File 1,Table 4: Activity of Blood Antioxidant Enzymes

Figura 8. Actividad específica Catalase

Kruskal-Wallis, $\chi^2(3) = 9.22$, $p = 0.026$, $n = 32$

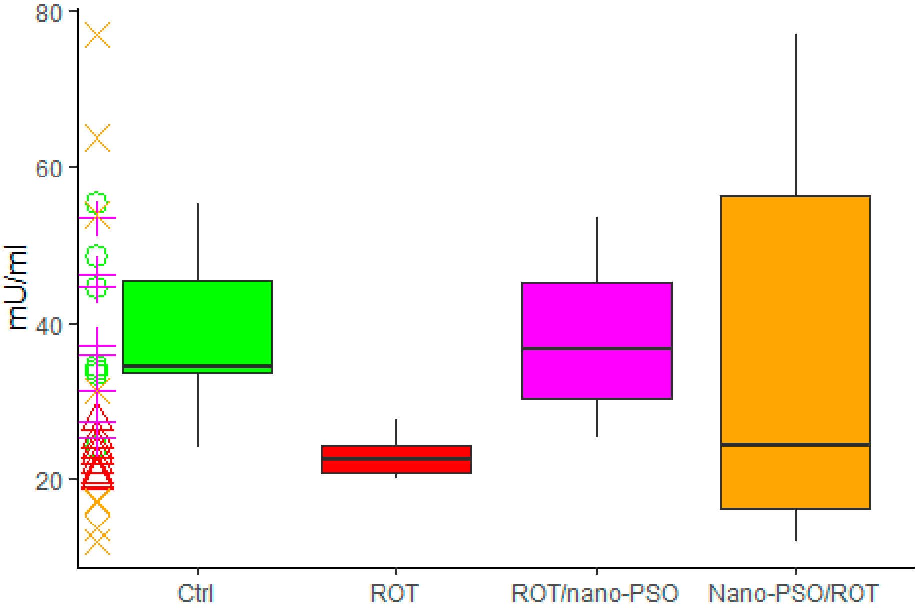

Figura 9. Actividad específica NADPH

Kruskal-Wallis, $\chi^2(3) = 8.49$, $p = 0.037$, $n = 32$

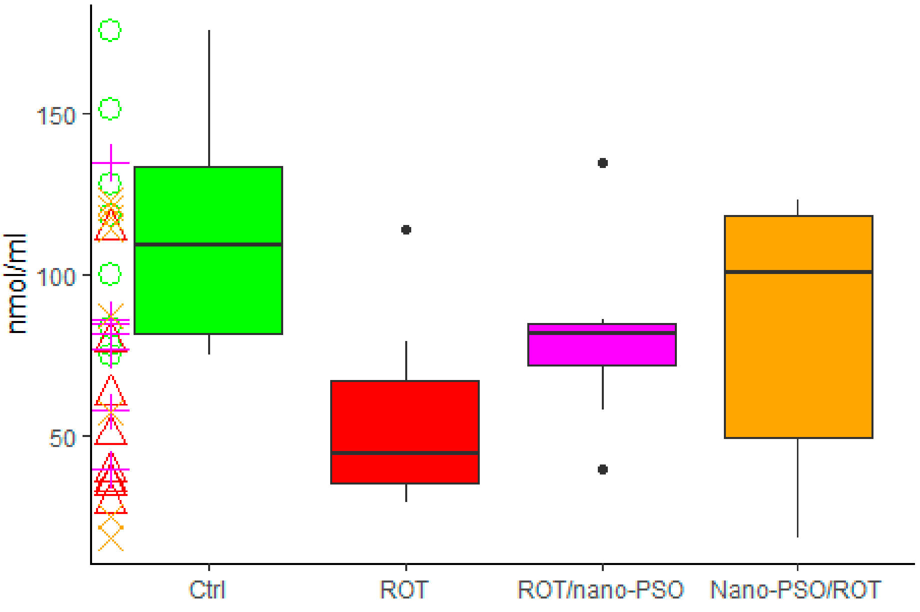

Figura 10. Actividad específica SOD

Kruskal-Wallis, $\chi^2(3) = 12.33$, $p = 0.0063$, $n = 32$

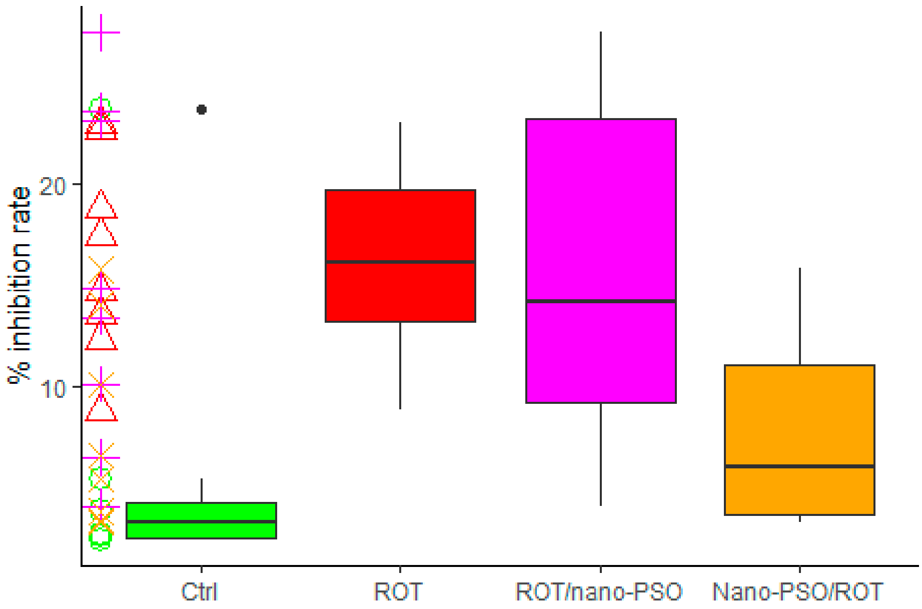

File 1,Table 5: Inclined beam test (Latency in seconds)

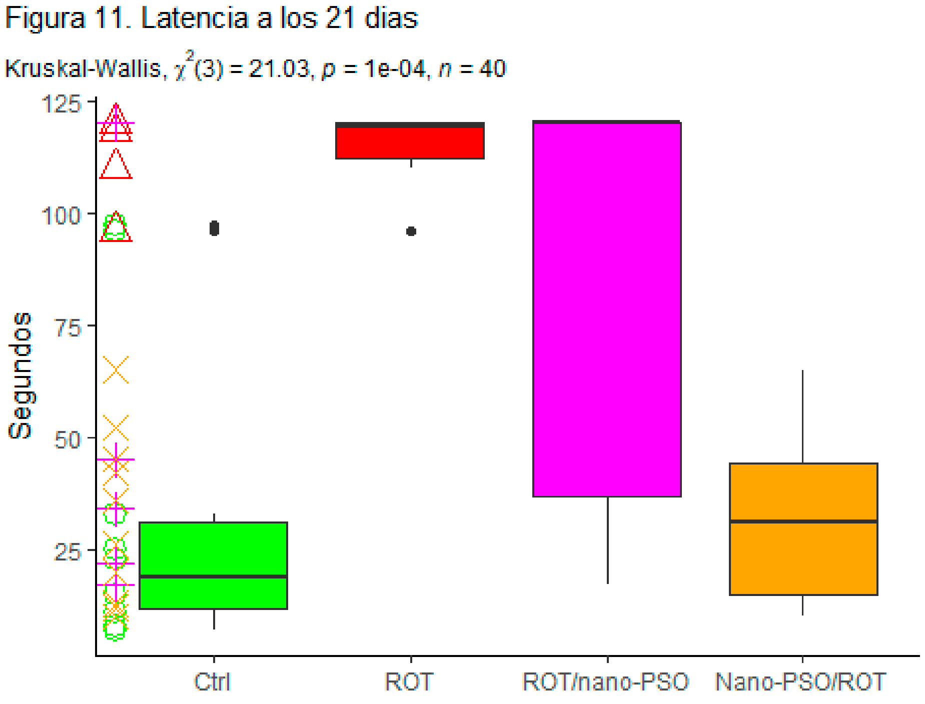

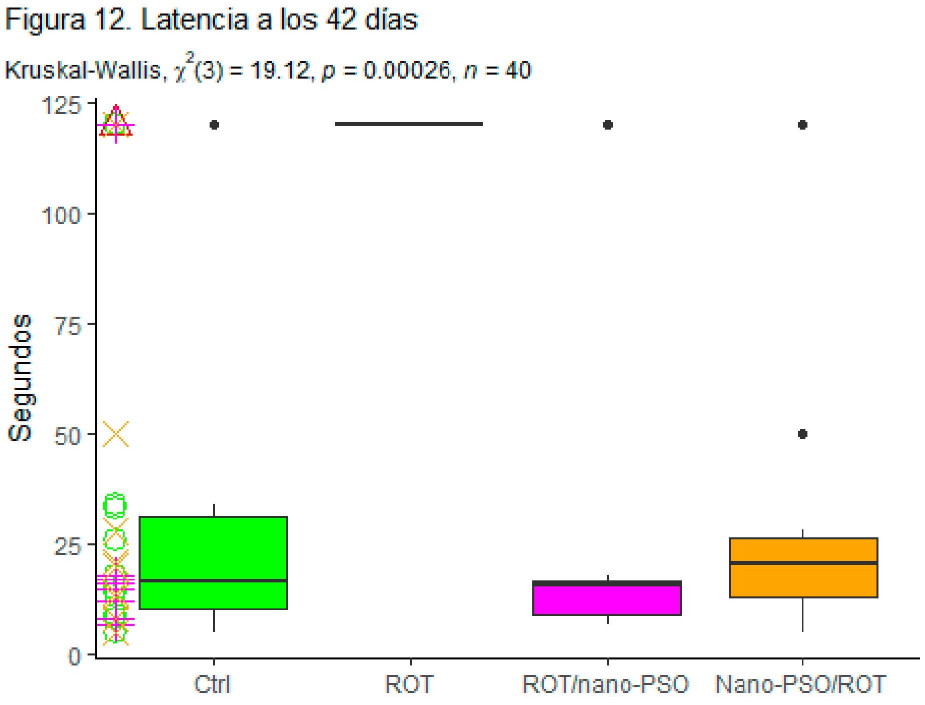

File 1,Table 6: Blood Biochemistry

Figura 13. Glucosa

Kruskal-Wallis, $\chi^2(3) = 10.55$, $p = 0.014$, $n = 32$

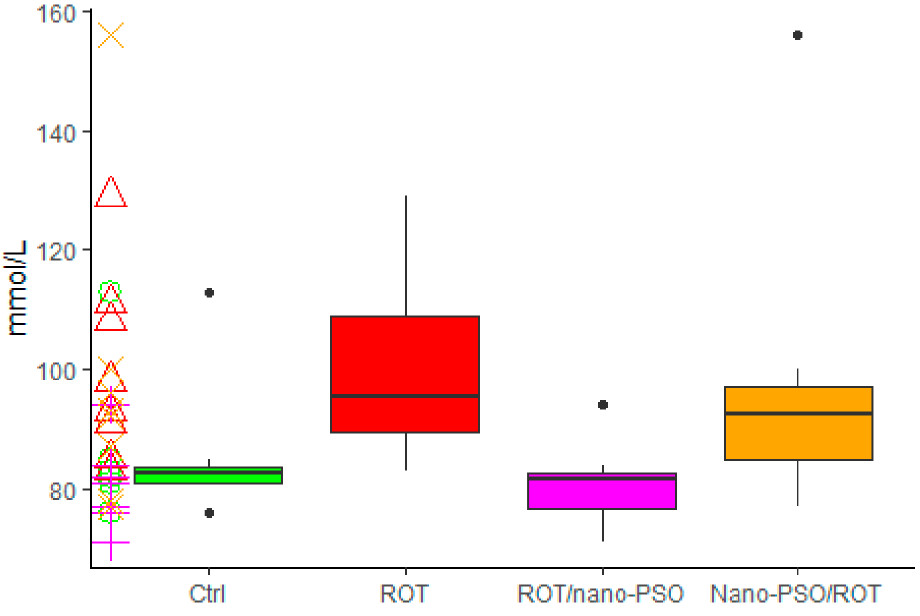

Figura 14. Triglicéridos

Kruskal-Wallis, $\chi^2(3) = 0.98$, $p = 0.8$, $n = 32$

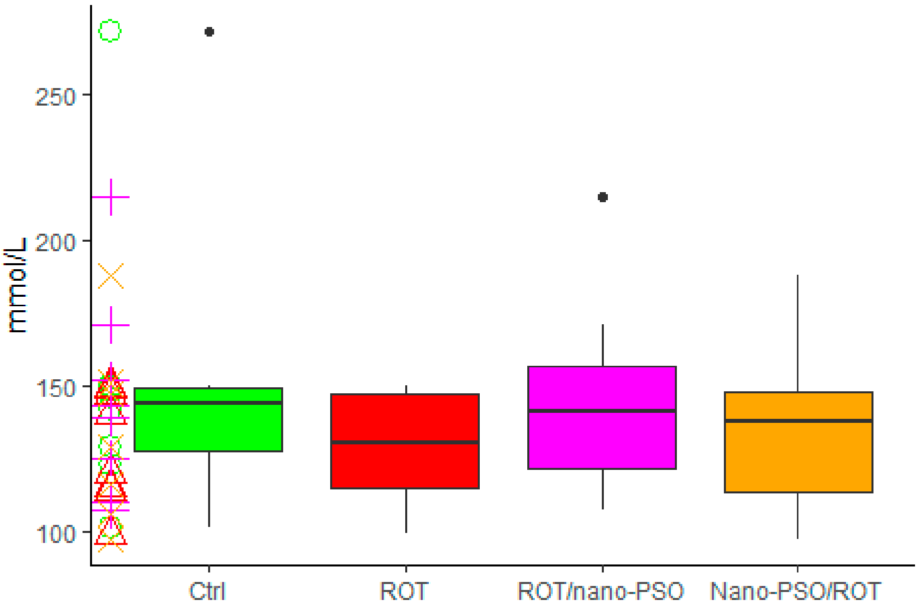

Figura 15. Colesterol

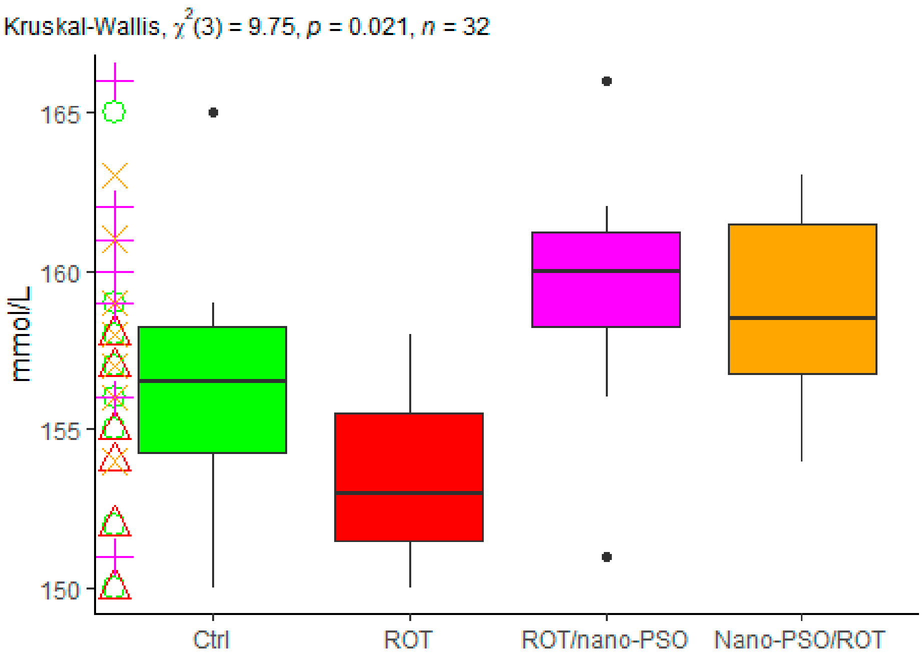

File 2,Table 1: Neurotransmitters and Metabolites in Caudate nucleus

Figura 16. Concentración DA en Caudate nucleus

Kruskal-Wallis, $\chi^2(3) = 4.95$, $p = 0.18$, $n = 32$

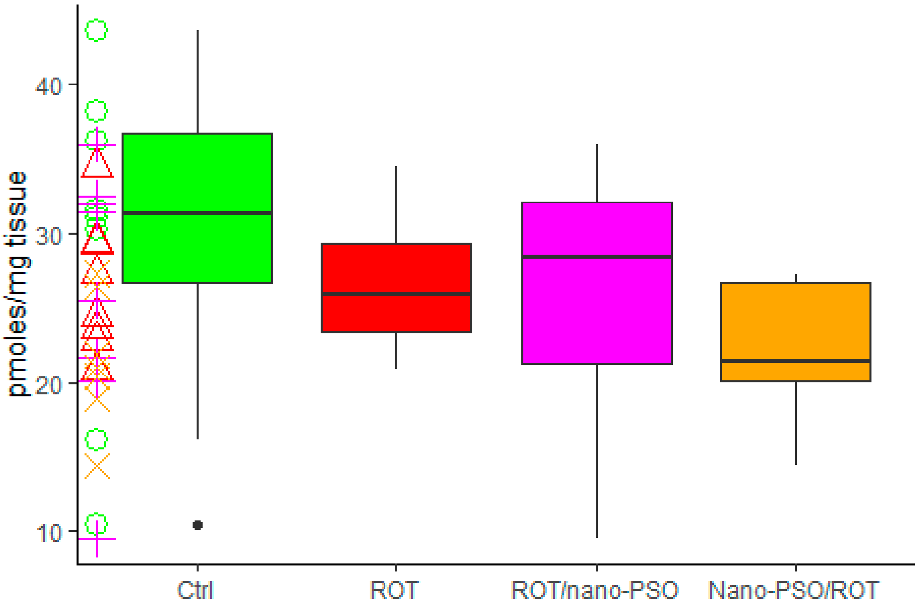

Figura 17. Concentración DOPAC en Caudate nucleus

Kruskal-Wallis, $\chi^2(3) = 12.54$, $p = 0.0057$, $n = 32$

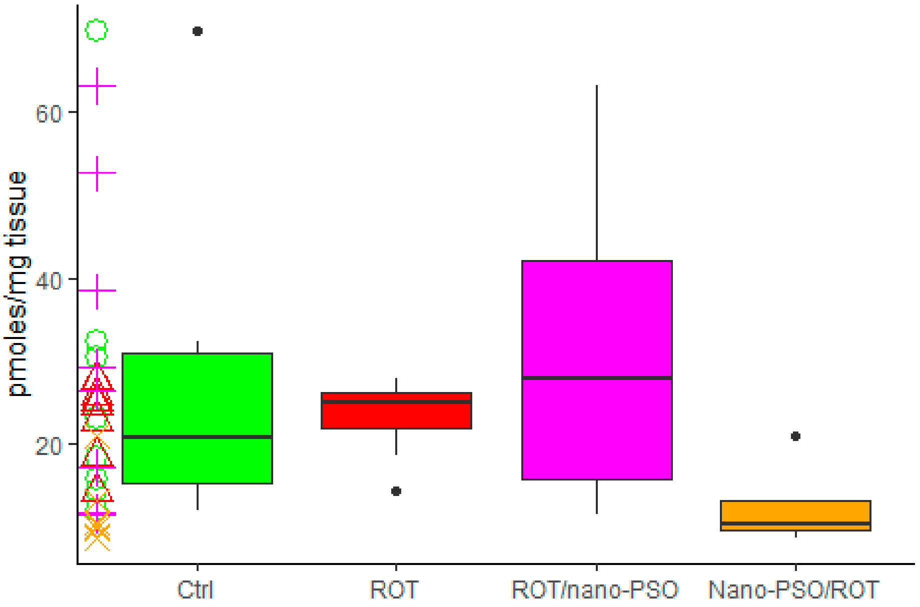

Figura 18. Concentración DA/DOPA en Caudate nucleus

Kruskal-Wallis, $\chi^2(3) = 8.63$, $p = 0.035$, $n = 32$

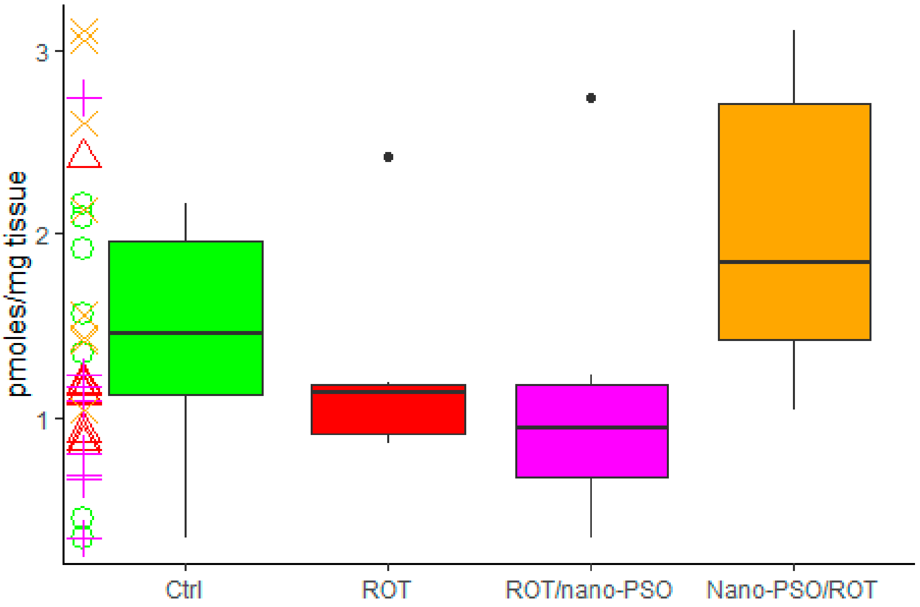

Figura 19. Concentración 5HT en Caudate nucleus

Kruskal-Wallis, $\chi^2(3) = 2.71$, $p = 0.44$, $n = 32$

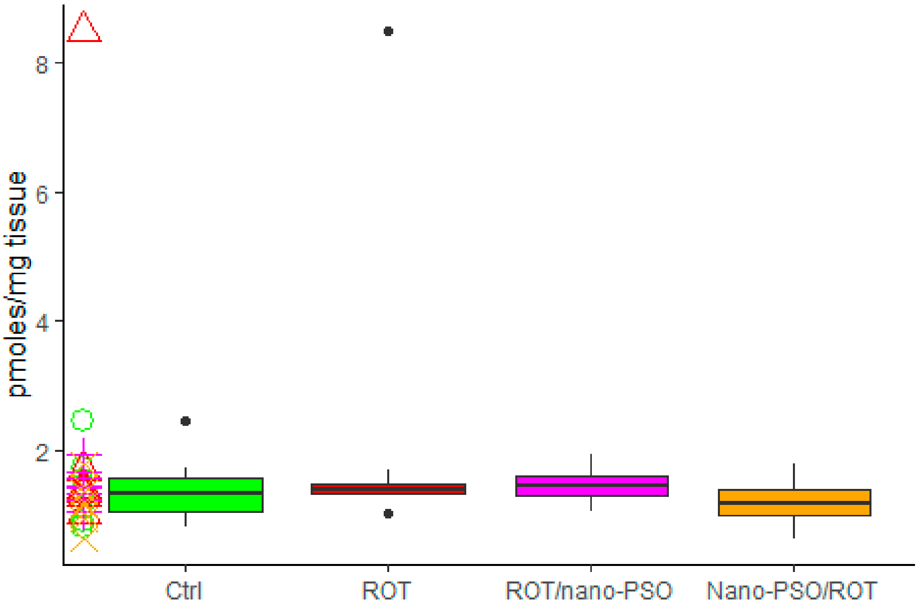

File 2,Table 2: Neurotransmitter and Metabolites in S. nigra

Figura 20. Concentración DA en s. nigra

Kruskal-Wallis, $\chi^2(3) = 10.08$, $p = 0.018$, $n = 15$

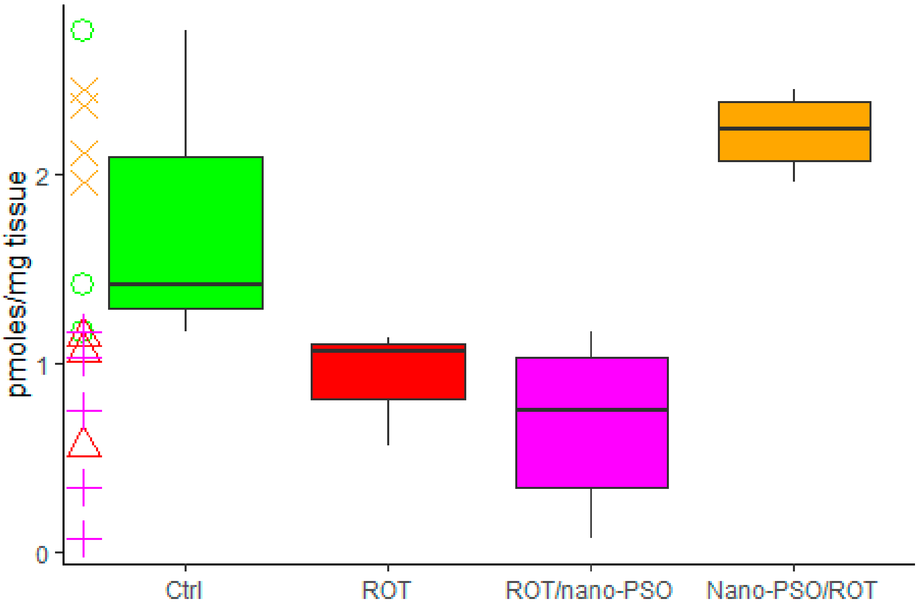

Figura 21. Concentración DOPAC en S. nigra

Kruskal-Wallis, $\chi^2(3) = 7.31$, $p = 0.063$, $n = 15$

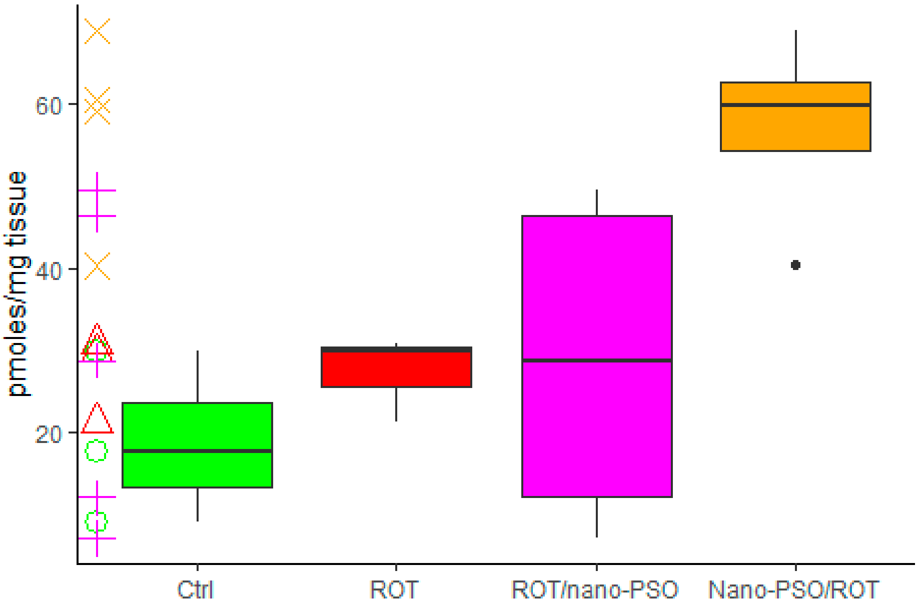

Figura 22. Concentración DA/DOPA en S. nigra

Kruskal-Wallis, $\chi^2(3) = 9.02$, $p = 0.029$, $n = 15$

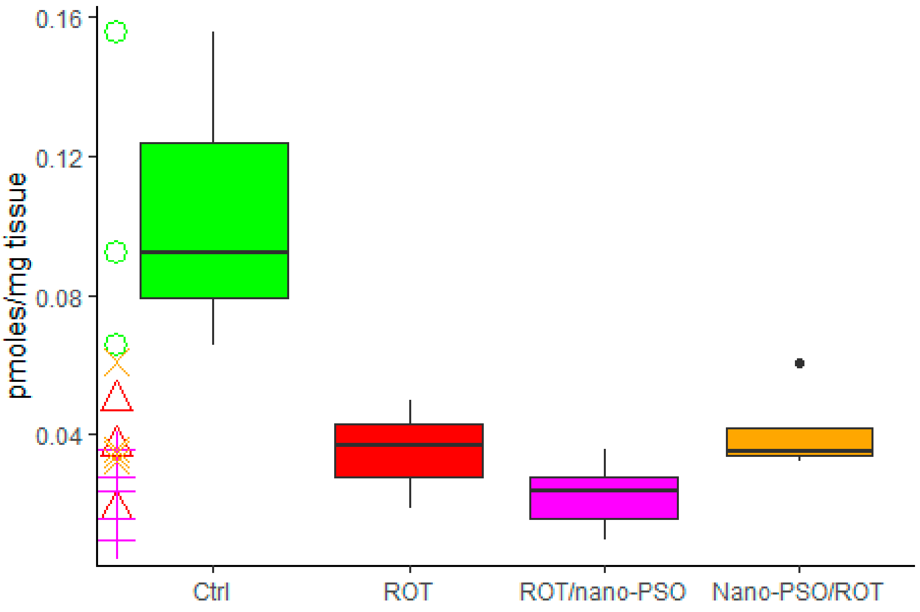

Figura 23. Concentración 5HT en S. nigra

Kruskal-Wallis, $\chi^2(3) = 6.06$, $p = 0.11$, $n = 15$

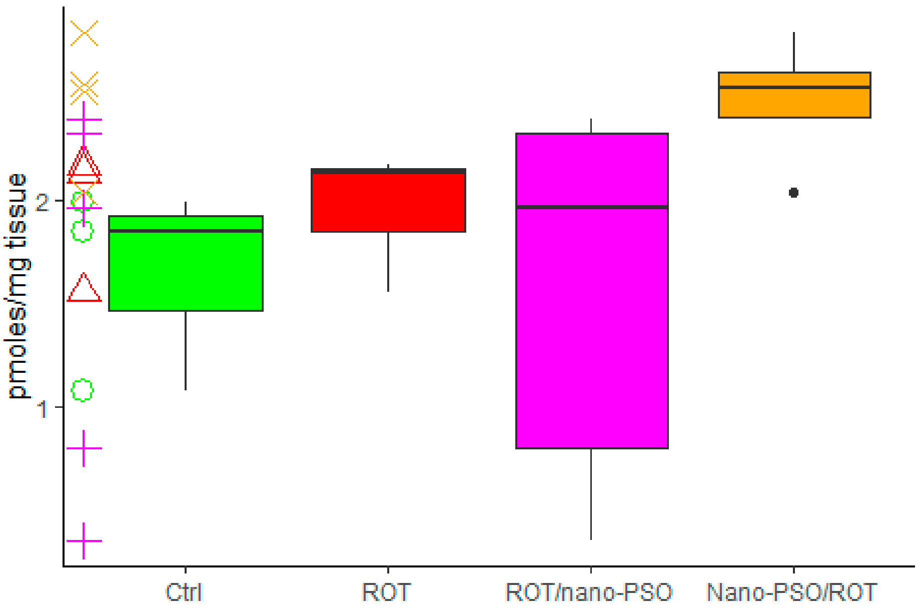

File 3,Table 1: Relative gene expression in Caudate nucleus

Figura 24. Expresión relativa de genes (CAT) en Caudate nucleus

Kruskal-Wallis, $\chi^2(3) = 18.89$, $p = 0.00029$, $n = 28$

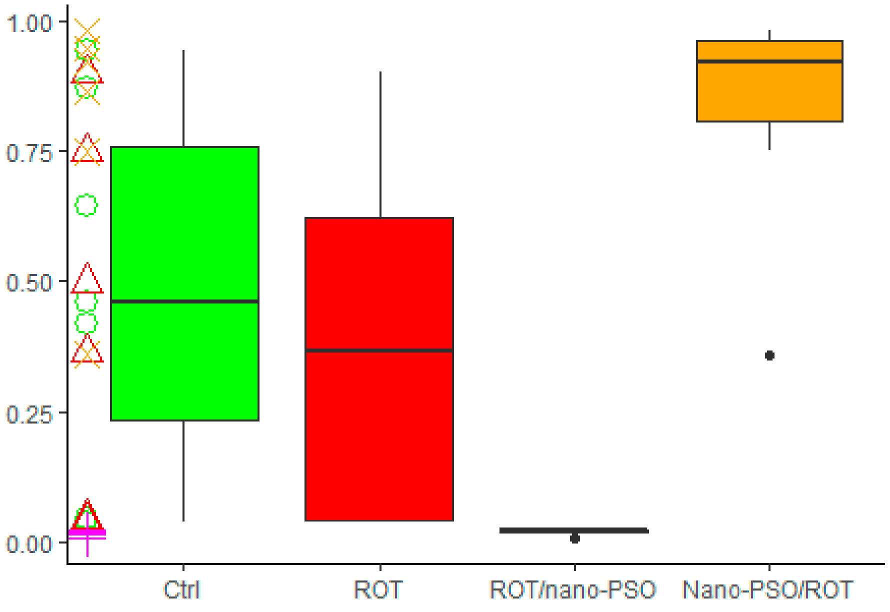

Figura 25. Expresión relativa de genes (GPX1) en Caudate nucleus

Kruskal-Wallis, $\chi^2(3) = 17.25$, $p = 0.00063$, $n = 28$

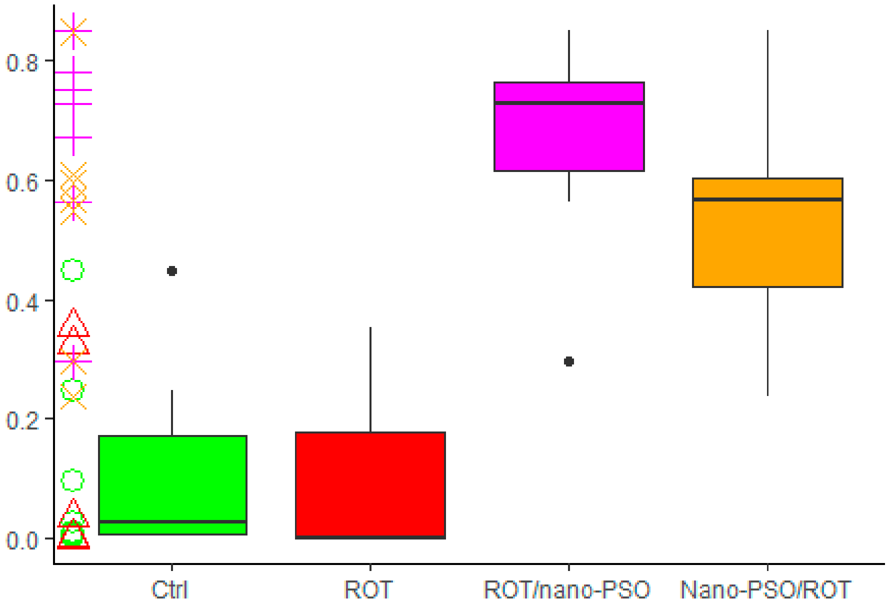

Figura 26. Expresión relativa de genes (SOD) en Caudate nucleus

Kruskal-Wallis, $\chi^2(3) = 2.39$, $p = 0.5$, $n = 28$

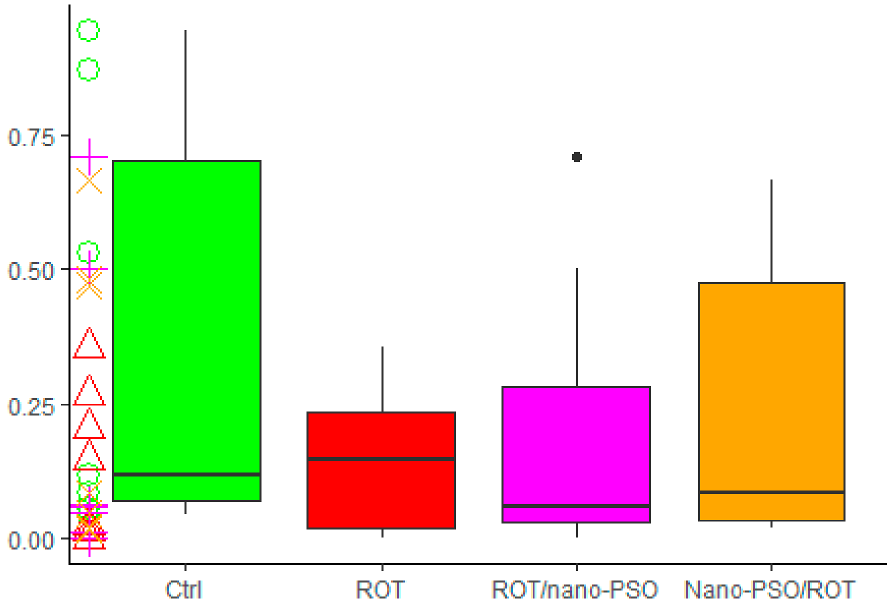

Figura 27. Expresión relativa de genes (SNCA) en Caudate nucleus

Kruskal-Wallis, $\chi^2(3) = 9.76$, $p = 0.021$, $n = 28$

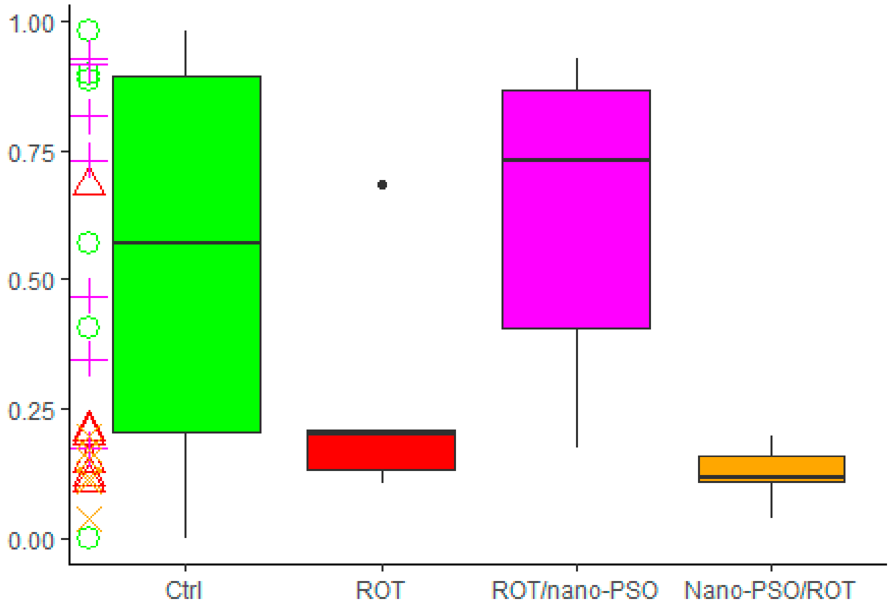

Figura 28. Expresión relativa de genes (TH) en Caudate nucleus

Kruskal-Wallis, $\chi^2(3) = 7.18$, $p = 0.066$, $n = 28$

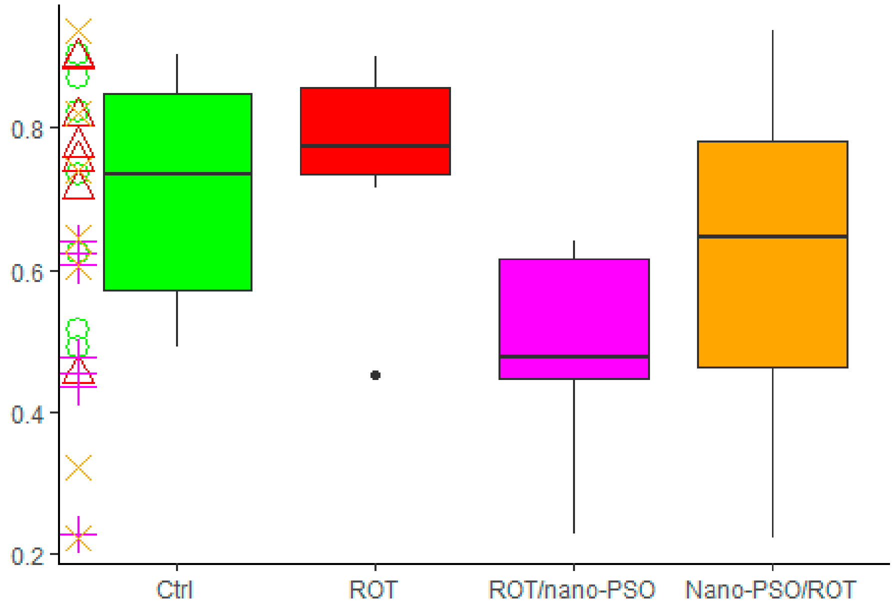

Figura 29. Expresión relativa de genes (GLUT3) en Caudate nucleus

Kruskal-Wallis, $\chi^2(3) = 11$, $p = 0.012$, $n = 28$

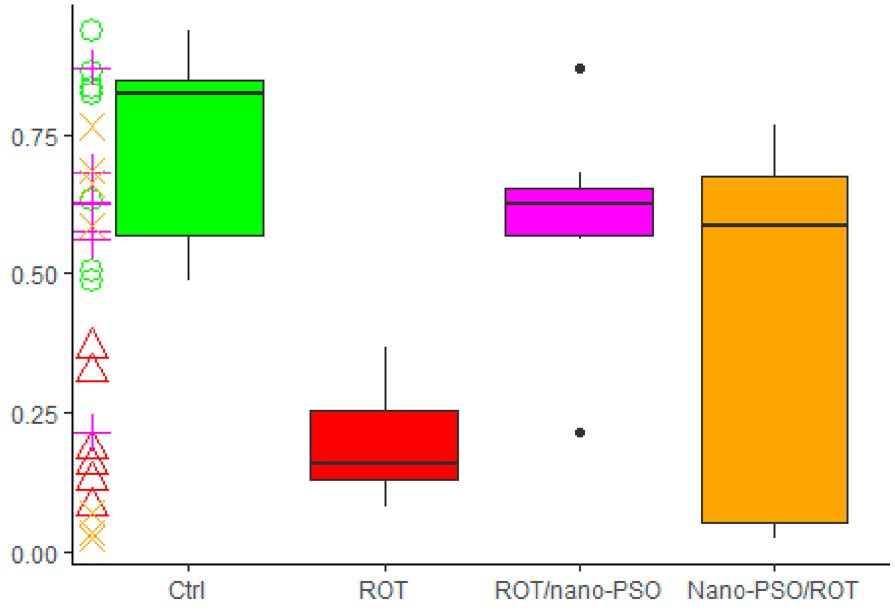

Figura 30. Expresión relativa de genes (GLUT4) en Caudate nucleus

Kruskal-Wallis, $\chi^2(3) = 7.09$, $p = 0.069$, $n = 28$

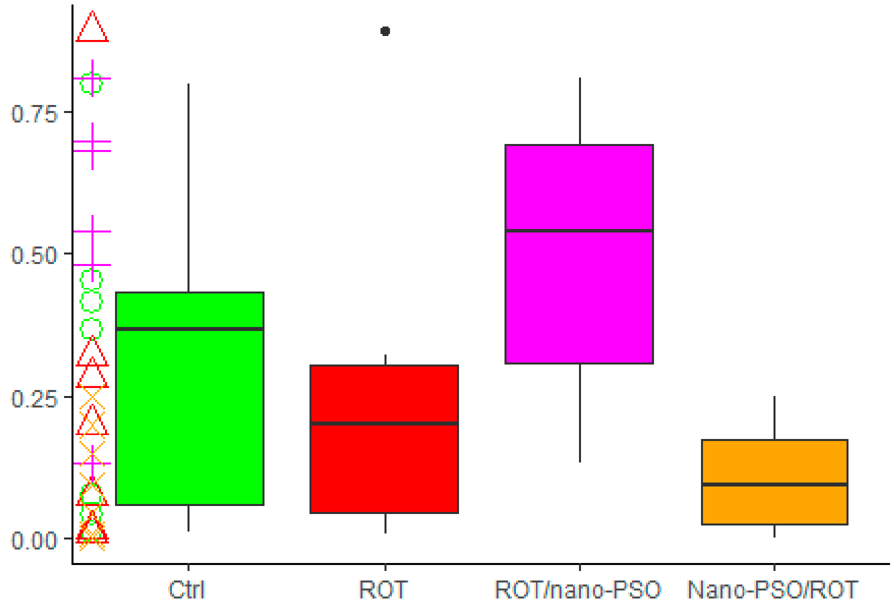

Supplement: Supplementary file 1 [file ijms-25-12635-s001.zip › Statistics Technical Report.pdf]
