# Supplementary figures and images for: Non-Categorical Analyses Identify Rotenone-Induced ‘Parkinsonian’ Rats Benefiting from Nano-Emulsified Punicic Acid (Nano-PSO) in a Phenotypically Diverse Population: Implications for Translational Neurodegenerative Therapies
Source: Int J Mol Sci. 2024 Nov 25;25(23):12635. doi: 10.3390/ijms252312635 (PMC11640963; doi:10.3390/ijms252312635)

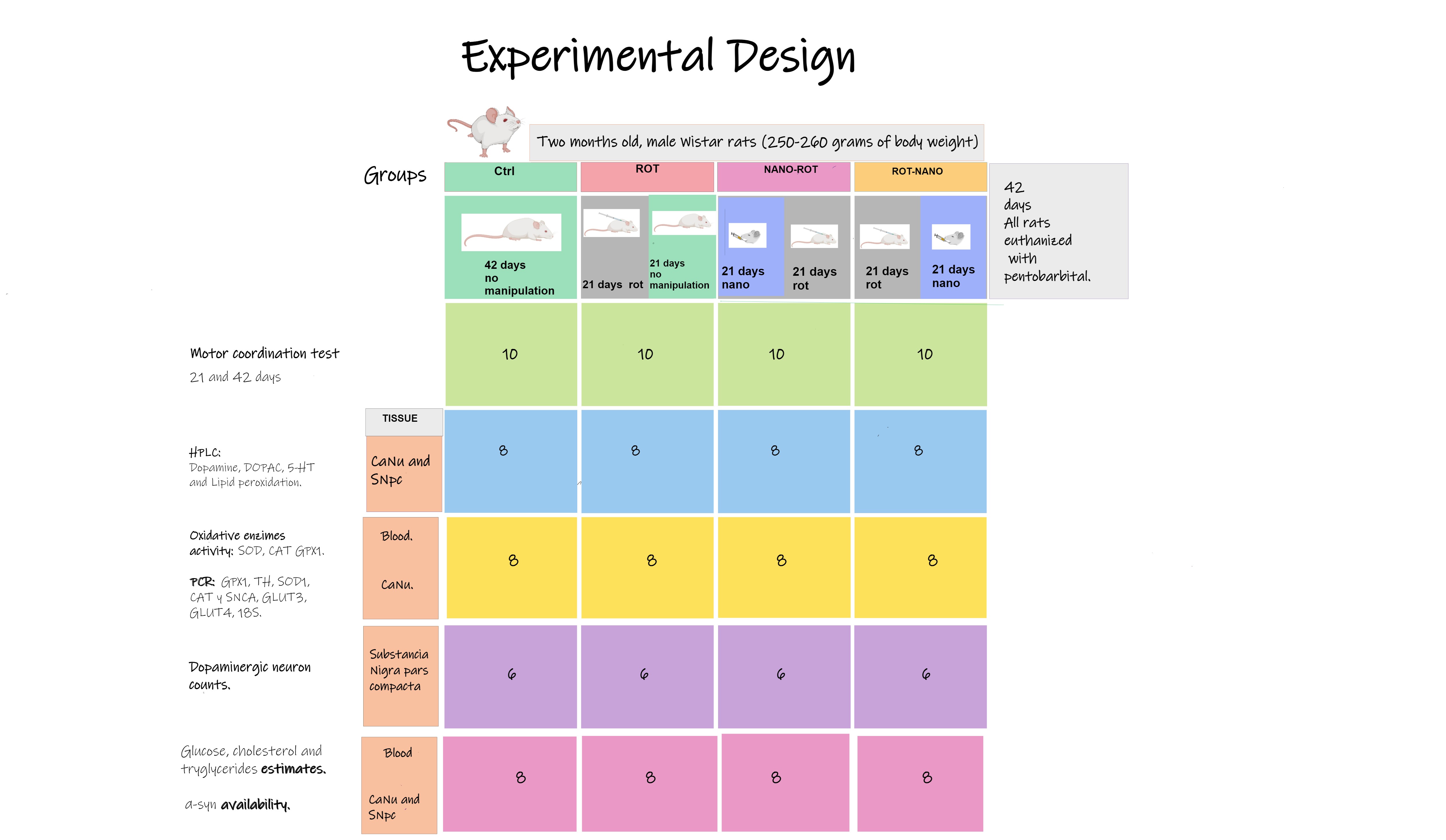

Supplement: Supplementary file 1 [file ijms-25-12635-s001.zip › Experimental design.jpg]
